# Mechanistic Investigations of a Hydrogen-Evolving Cobalt Diimine-Dioxime Complex in an Oxygen Environment: Roles of Secondary Coordination Sphere, Bro̷nsted Acid, and Axial Ligand

**DOI:** 10.1021/acs.inorgchem.4c03301

**Published:** 2025-02-25

**Authors:** Yu-Syuan Tsai, Yu-Wei Chen, Charasee Laddika Dayawansa, Hsuan Chang, Wen-Ching Chen, Jiun-Shian Shen, Tiow-Gan Ong, Glenn P. A. Yap, Vincent C.-C. Wang

**Affiliations:** † Department of Chemistry, National Sun Yat-sen University, Kaohsiung, Taiwan 80424, R.O.C.; ‡ Institute of Chemistry, Academia Sinica, Taipei, Taiwan 11529, R.O.C.; § Department of Chemistry and Biochemistry, University of Delaware, Newark, Delaware 19716, United States; ∥ Green Hydrogen Research Center, National Sun Yat-sen University, Kaohsiung, Taiwan 80424, R.O.C.

## Abstract

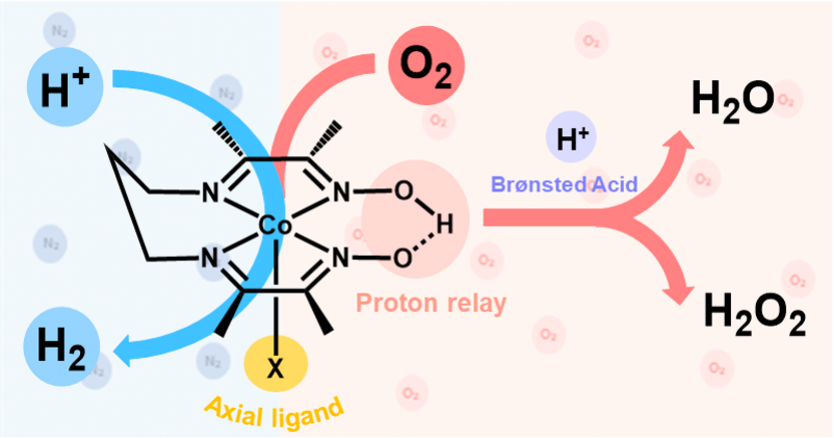

The concept of a secondary coordination sphere (SCS) has been widely adopted in designing molecular electrocatalysts to promote energy-conversion reactions, such as the hydrogen-evolution reaction (HER) or the reduction of carbon dioxide. The role of SCS in the oxygen-tolerant properties of molecular electrocatalysts is less explored. An HER electrocatalyst, the cobalt diimine-dioxime complex, is one of the metal complexes designed by the concept of SCS to facilitate HER and retain its reactivity in an oxygen environment. Nevertheless, the mechanism underlying its oxygen tolerance remains unclear. This study revealed that in the presence of molecular oxygen, the oxygen reduction reaction (ORR) predominates over the hydrogen evolution reaction (HER) for this cobalt complex. Further investigations suggest that intramolecular proton transfer through SCS and intermolecular proton transfer from exogenous proton sources mutually dictate the product selectivity of ORR between H_2_O_2_ and H_2_O, thereby determining the stability of the complex under HER. In addition, the choice of labile ligands has emerged as a useful factor in enhancing oxygen tolerance. These findings provide valuable design principles for developing oxygen-tolerant molecular catalysts and shed light on the reactivity and product selectivity controlled by the interplay of proton transfer routes.

## Abstract 2

The mechanism of the hydrogen evolution reaction (HER) catalyzed by cobalt diimine dioxime in the presence of molecular oxygen was examined. It was observed that the oxygen reduction reaction (ORR) is favored over the HER in the presence of molecular oxygen. The product distribution between H_2_O and H_2_O_2_ varies with the p*K*_a_ values of the Bro̷nsted acids, highlighting that the proton relay site is crucial in determining product selectivity.

## Introduction

In recent years, the development of cheap first-row transition metal complexes for fuel-forming reactions, such as hydrogen-evolution reaction (HER) and CO_2_-reducing reaction (CRR) catalysts, has been widely studied.^1,2^ Traditionally, the key design principles of molecular electrocatalysts evolve from structural mimics of the active sites inspired by biocatalysts, such as hydrogenases, carbon monoxide dehydrogenase, and porphyrin-containing enzymes.^3−5^ In recent years, research focus has shifted to the roles of the secondary coordination sphere or even the outer coordination sphere.^4^ The common strategy builds on the incorporation of the amine or hydroxyl group at the secondary coordination sphere (SCS) near an active site of an electrocatalyst. This group functions as a proton relay site or interacts with the intermediate species through electrostatic interaction.^3,6^ Such effects have shown drastically improved HER and CRR activity. Nevertheless, most of those molecular complexes plague the instability in the presence of molecular oxygen, which impedes practical applications.^7−9^

The primary factor for this instability arises from the higher tendency of O_2_ reduction reaction (ORR) to generate reactive oxygen species (ROS), such as H_2_O_2_ or O_2_^·–^, than HER and CRR due to a more positive reduction potential as shown below.^10^ The high reactivity of ROS could inactivate or even damage electrocatalysts.^9,11,12^ Furthermore, from a practical point of view, a small fraction of the O_2_ generated from the anode side in a water-splitting cell can leak through a membrane separator into the cathodic compartment, which can be detrimental to oxygen-sensitive electrocatalysts. The ROS can oxidatively degrade organic ligands or polymer membranes.^13^ So far, only a few molecular HER or CRR electrocatalysts are reported to promote fuel-forming reactions in aerobic conditions.^7,8,12,14−17^ Some of them have successfully applied the concept of SCS to increase their oxygen tolerance but the detailed mechanism and roles of SCS are still less explored.^7^ For example, very recent studies have demonstrated that the acidity of both the SCS and exogenous proton sources can impact the electrocatalytic performance of SCS-modified iron-porphyrin complexes on CO_2_ reduction.^18,19^

Eq (1):^10^
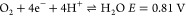
Eq (2):^10^
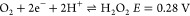
Eq (3):^20^
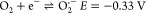
Eq (4):
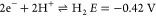
Eq (5):^21^
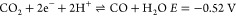

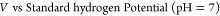


**Scheme 1:**
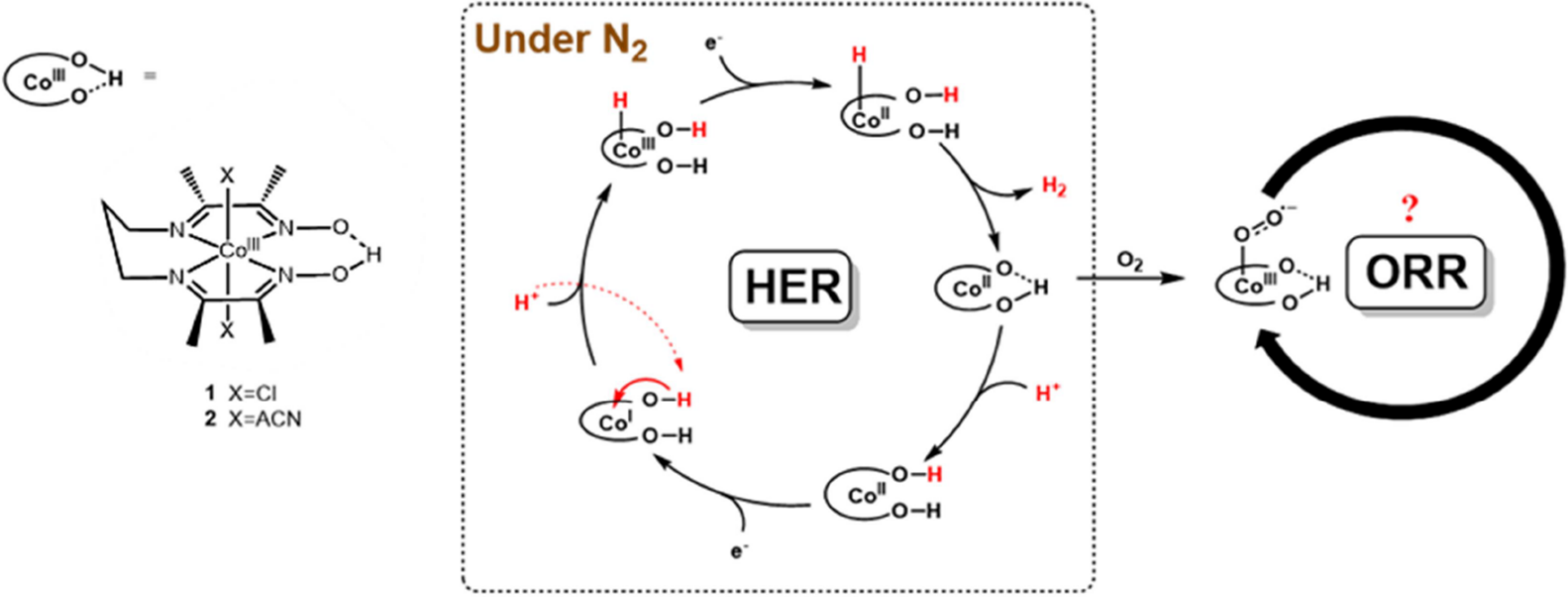
Structures of Two Cobalt Diimine-Dioxime Complexes Used for This Study and the Reaction Mechanism for HER Mediated by the Cobalt complex^27^, Nevertheless, it remains unclear how a cobalt diimine-dioxime complex performs HER in the presence of molecular oxygen.

The cobalt diimine-dioxime complex, as shown in Scheme 1, is one of the prototypical HER complexes that utilized the concept of SCS to promote HER reaction with moderate overpotential requirement (∼0.2 V).^22−24^ Moreover, it represents one of few examples that possess the properties of oxygen-tolerant HER electrocatalysts.^16^ Therefore, the wide incorporation of this complex into fuel-forming devices and applications, such as a fuel-forming electrolyzer and dye-sensitized photoelectrochemical cell has been demonstrated.^25,26^ It is well acknowledged that the mechanism of HER catalyzed by the cobalt metal center is between Co(I) and Co(III) with the oxime moiety acting in a proton-relaying role to facilitate HER as shown in Scheme 1. It is interesting to understand whether the function of the proton-relaying site can promote oxygen reduction reaction (ORR), thereby impacting oxygen tolerance. Previously, the Artero group directly grafted the poorly water-soluble dichlorido diimine-dioxime cobalt(III),^16^**1** onto an electrode and demonstrated that it can promote HER in the presence of molecular oxygen. They further observed that complex **1** catalyzes ORR, producing water and hydrogen peroxide in a 3:1 ratio at pH 4.5. To investigate the electrocatalytic mechanism of the ORR catalyzed by complex **1** and its influence on HER in an oxygen-rich environment, we examined the electrocatalytic behavior of both the ORR and HER in acetonitrile. Acetonitrile as an organic solvent minimizes complications from proton sources in aqueous solution, allowing for a clearer assessment of the independent effects of proton sources on both HER and ORR. In addition, the roles of two essential elements for fuel-forming reactions, Bro̷nsted acids, and labile ligands, were also further examined to understand their impact on oxygen tolerance. Finally, the general design principles for oxygen-tolerant molecular fuel-forming electrocatalysts learned from those results are discussed.

## Results

### Hydrogen Evolution Reaction Mediated by 1 in the Presence of Molecular Oxygen

**Figure 1:**
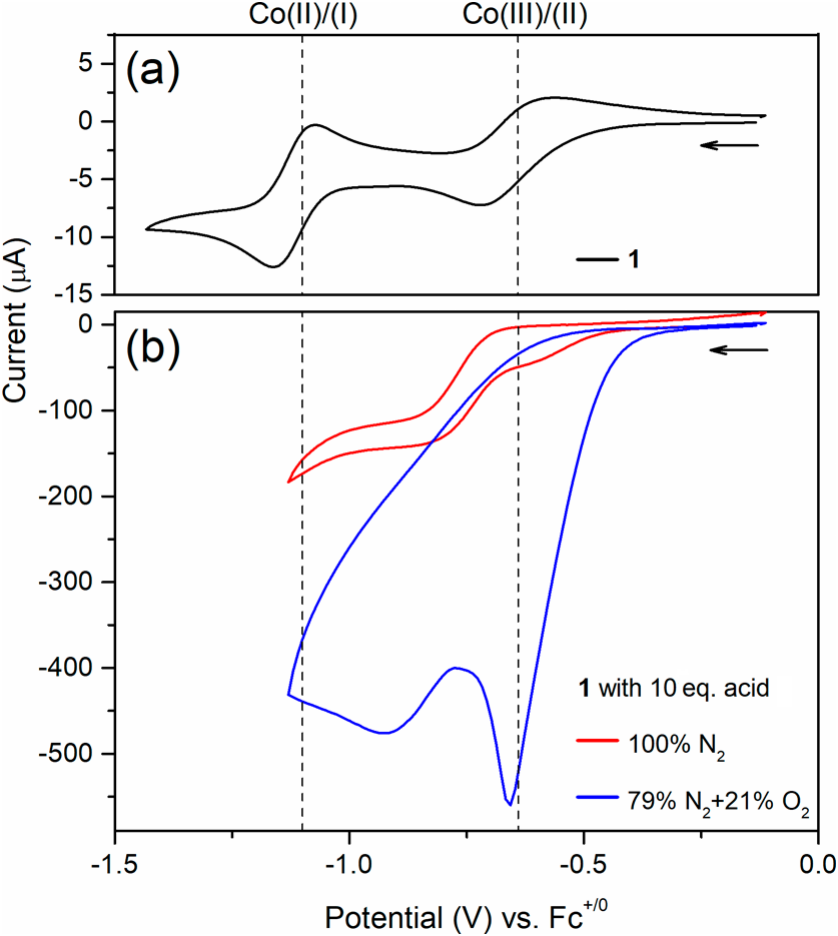
Cyclic voltammograms of **1** (0.5 mM) in ACN under different conditions. (a) The voltammogram obtained under 100% N_2_ with the scan rate of 0.1 V/s. (b) The voltammograms were collected in the presence of 10 equiv of *p*-cyanoanilinium tetrafluoroborate with 3 V/s under two different gas compositions, respectively. The red trace: 100% nitrogen. The blue trace: 79% nitrogen and 21% oxygen. To calculate the TOF, the peak value *i*_p_ used for eq 6 was corrected by subtracting 3 μA from the background capacitance current.

Figure 1a reveals the typical voltammograms of **1** in acetonitrile (ACN) in the presence of N_2_ where two redox couple peaks correspond to a quasi-reversible peak for a Co(III)/(II) couple around −0.64 V and a reversible peak for a Co(II)/(I) couple around −1.11 V vs Fc^+/0^ respectively (here all reported potentials are versus ferrocenium/ferrocene (Fc^+/0^)). All reduction potential values obtained here are similar to the values reported from the same or similar cobalt complexes reported before.^22,23^

When adding 10 equiv of *p*-cyanoanilinium tetrafluoroborate as a proton source, the canonical S-shape of the electrocatalytic voltammograms was observed at 3 V/s as shown in Figure 1b (red trace). The limiting current, ca. 143 μA did not further increase as the scan rate increased (Figure S1), which indicated the pure kinetic region has been achieved.^28^ Therefore, according to eq 6, the turnover frequency (TOF) for HER mediated by **1** in the presence of *p*-cyanoanilinium is 84 s^–1^.^29^ Further details for determining the TOF value are available in the SI. This value is slightly smaller than the TOF value of 180 s^–1^ reported from the dibromido cobalt diimine-dioxime complex.^23^
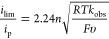
6where *i*_lim_ is the limiting current in the presence of substrate; *i*_p_: the peak current in the absence of substrate; *n*: the number of electrons involved with the reaction; *R*: ideal gas constant, *T*: temperature (Kelvin), *F*: Faraday constant; υ: scan rate (V/s). *k*_obs_: pseudo-first-order rate constant (i.e., TOF, s^–1^).

After adding O_2_ to the headspace with a gas ratio of 21% O_2_ and 79% N_2_, the electrocatalytic current further increased approximately three times as shown in the blue trace of Figure 1b. The onset potential shifted positively from approximately −0.45 to −0.25 V before the redox peak of a Co(III)/(II) couple. These results indicated that the formation of the Co(II) redox state begins with ORR. To understand the nature of HER in the presence of O_2_, a gas chromatograph (GC), and a rotating ring disk electrode (RRDE) system were used to examine product distribution.

**Figure 2:**
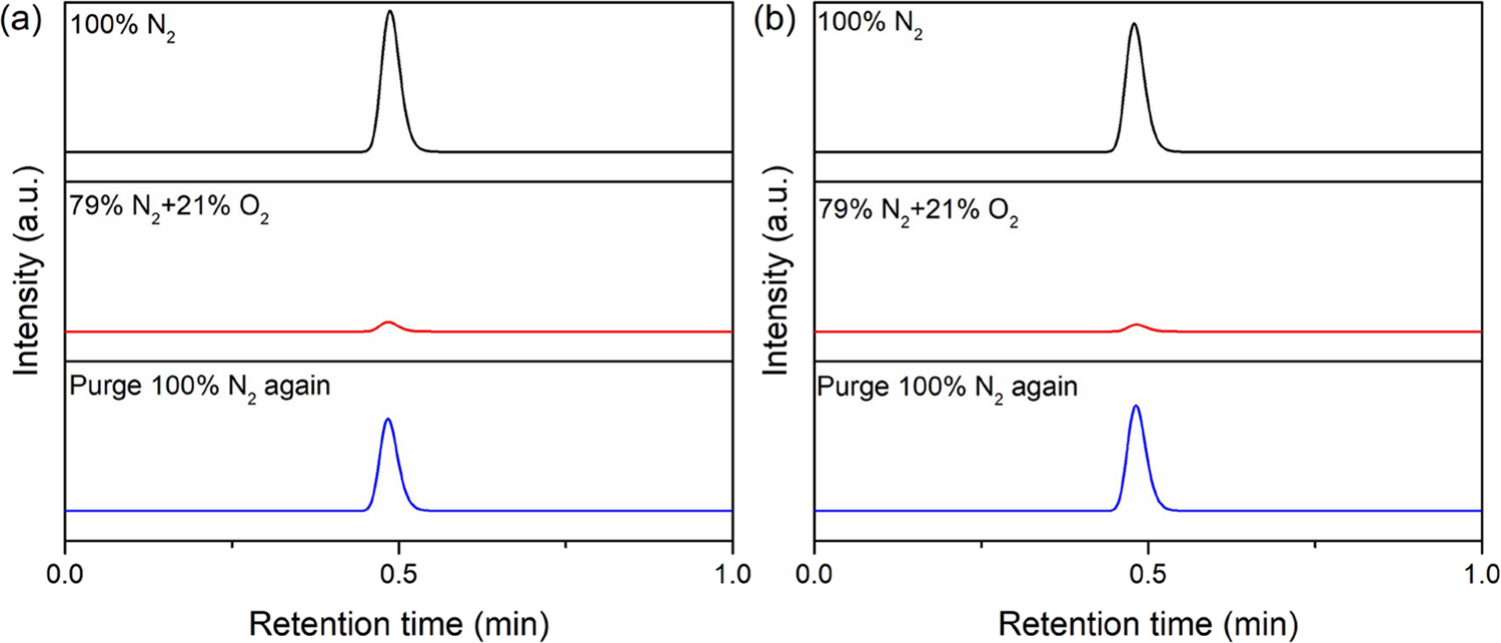
GC results for bulk electrolysis of **1** (0.5 mM) with acid (50 mM) in acetonitrile under various gas compositions. The electrolyzing potential was applied at −1.2 V vs Fc^+/0^ for 9 min. In the presence of (a) *p*-cyanoanilinium and (b) *p*-methoxyanilinium, respectively.

First, the electrolyzing potential, −1.2 V (which is approximately 0.1 V more positive before the reduction of oxygen to superoxide occurs as shown in Table S1) was poised to probe product distribution under different headspace compositions in the presence of *p*-cyanoanilinium (p*K*_a_ = 7.0).^30^ As expected, under 100% N_2_ headspace, only hydrogen gas was detected by GC over 9 min of electrolysis as shown in Figure 2a, which corresponds to 89% ± 5% Faradaic efficiency (FE). Unexpectedly, only a negligible amount of hydrogen gas was observed after the gas compositions were changed to 21% O_2_ and 79% N_2_. The reintroduction of 100% N_2_ into the electrochemical cell leads to the formation of H_2_ albeit with lower FE, 76% ± 12%. The rinse test showed no increase in electrocatalytic current in the presence of acid alone, suggesting that heterogeneous catalysts were not formed on the electrode surface (see Figure S4). Similar conditions for electrochemical studies also revealed no formation of cobalt nanoparticles on the glassy carbon electrode surface by the X-ray photoelectron spectroscopy examination after electrolysis.^31^ In addition, no noticeable difference in FE was observed if the electrode was polished between different gas compositions (Figure S6) To further examine the role of acid, a much weaker acid, *p*-methoxyanilinium (p*K*_a_ = 11.9)^30^ was used. The same trends were observed, as shown in Figure 2b. The FE for HER in the presence of *p*-methoxyanilinium is 103 ± 26% but only a trace amount of H_2_ was detected when purging with 21% O_2_ and 79% N_2_ headspace. A lower FE, 64 ± 23%) was observed when reintroducing N_2_ into the system. It is worth noting that only trace amounts of hydrogen gas (less than 5%) were observed across all measurements of both acids in the presence of 21% of O_2_ and 79% of N_2_. These observations are in contrast with HER accompanied by ORR observed in aqueous solution.^16^ This difference is likely due to the higher solubility of O_2_ in ACN (8.1 vs 1.3 mM in water).^32,33^ When introducing a lower amount of O_2_ (7%) for electrocatalysis in the presence of *p*-cyanoanilinium, the faradaic efficiency for HER increased to 16.8 ± 5% (Figure S7).

To further explore the cyclability of HER recovery, additional N_2_/O_2_ cycling tests in the presence of *p*-cyanoanilinium were conducted, revealing a decline in Faradaic efficiency for HER as the number of cycles increased (Figure S8). The long-term test (30 min electrolysis for each gas composition) also showed that FE drops from 99% ± 1% to 88% ± 4%. A larger decrease in electrocatalytic current was observed compared to the current over 9 min electrolysis (Figure S2 vs Figure S9b). These are probably due to the instability of **1** in the presence of acid in the long-term electrolysis^34^ or inactivation of **1** by H_2_O_2_ formed (vide infra). The further rinse test suggested that a small amount of electrodeposited catalyst may form during prolonged electrolysis, but its contribution to the electrocatalytic current is minimal (Figure S10).

**Figure 3:**
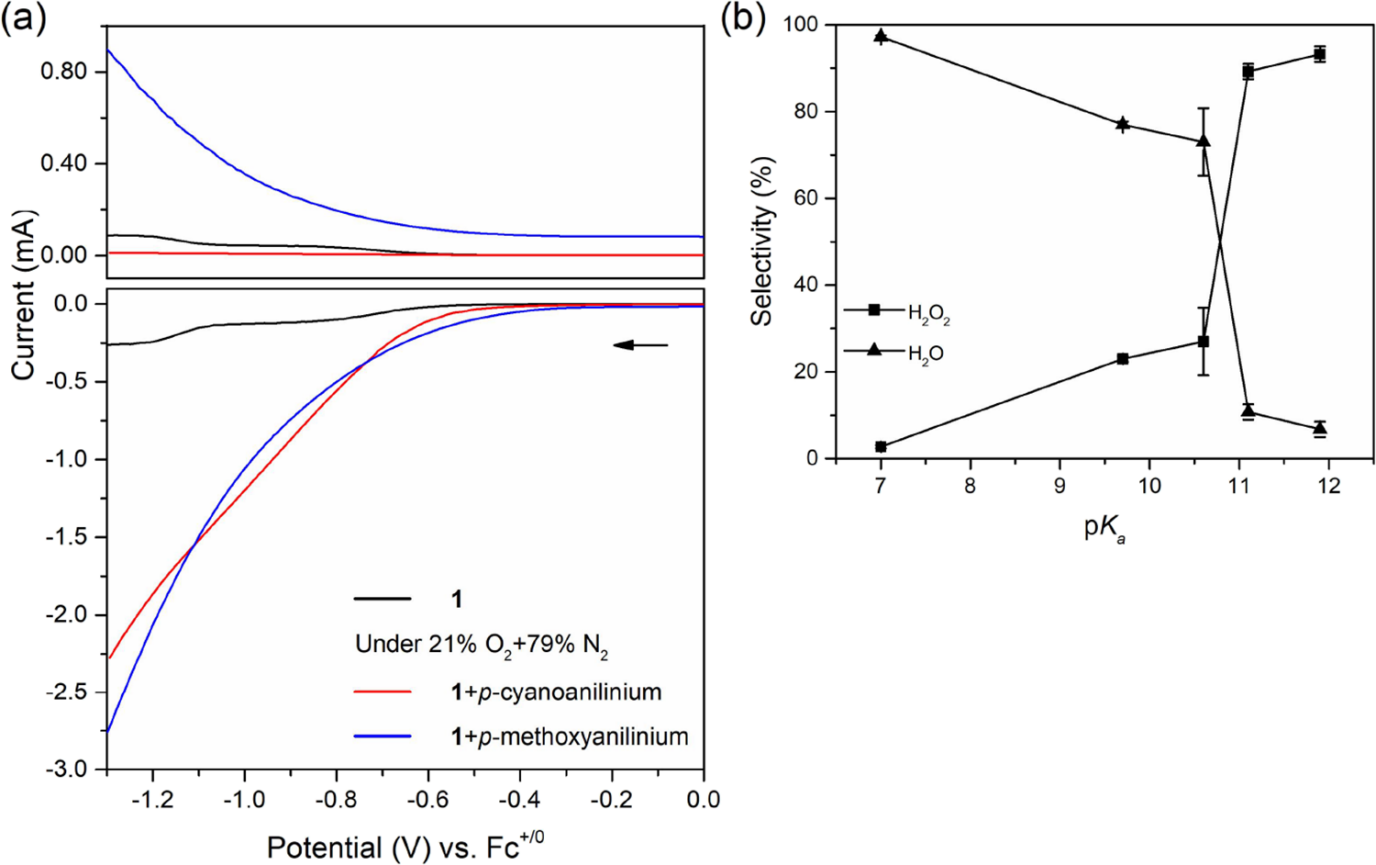
(a) RRDE voltammograms of **1** under different conditions. The upper and lower voltammograms were collected from the ring and disk electrode, respectively. The black trace: **1** (0.5 mM) in the absence of acid under 100% N_2_. The red and blue trace: **1** in the presence of 50 mM *p*-cyanoanilinium (red trace) or *p*-methoxyanilinium (blue trace) under 21% O_2_ and 79% N_2_ in acetonitrile. Scan rate: 0.01 V/s. (b) The product distribution of ORR in the presence of 100 equiv of different acids at −1.2 V under the gas ratio of 21% O_2_ and 79% N_2_.

To further probe product distribution between H_2_O and H_2_O_2_ from the ORR reaction in the presence of different gas compositions and acids, an RRDE system was employed. As shown in the linear sweep voltammetry of Figure 3a, the potential of the disk electrode was varied from 0 to −1.3 V with a scan rate of 0.01 V/s in the presence of different Bro̷nsted acids and gas compositions. The number of electrons involved with ORR (i.e., the formation of H_2_O and H_2_O_2_ respectively as shown in Table S1) is calculated by eq 7:^35^
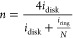
7where *i*_disk_ and *i*_ring_ are the currents measured from the disk electrode and ring electrode, respectively. *N* is the collection efficiency for the RRDE system, which was 0.37 in our system. When hydrogen peroxide is the dominant product, the *n* value is expected to be close to 2. As opposed, the *n* value is close to 4 when water is the main product. The ratio of the formation of H_2_O_2_ and H_2_O from the ORR can be further estimated by the following relations:
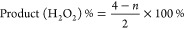
8
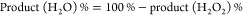
9

Interestingly, we observed different product distributions under 21% O_2_ and 79% N_2_ when adding different p*K*_a_ acids as summarized in Figure 3b. In the presence of a strong acid, *p*-cyanoanilinium, the *n* value, 3.7, was obtained at the poised potential, −1.2 V. This value suggests that the main products from ORR were water. In comparison, hydrogen peroxide as the dominant ORR product was observed in the presence of a weak acid, *p*-methoxyanilinium. The product selectivity was not apparently affected by varying the concentration of O_2_ from 7% O_2_ to 100% O_2_ between two acids (Figure S12). These results indicate that the p*K*_a_ value of the acid plays a crucial role in modulating product selectivity. In addition, aniline and its derivatives are known to undergo electropolymerization under oxidizing potential.^36^ Further control experiments showed that such interference is negligible at the ring electrode (Figure S11). A further wide p*K*_a_ range of acids from *p*-chloroanilinium (p*K*_a_ = 9.7), anilinium (p*K*_a_ = 10.6), *p*-*tert*-butylanilinium (p*K*_a_ = 11.1)^30^ were used to examine the role of acidity. As shown in Figure 3b, the dominant product is H_2_O when the presence of acid p*K*_a_ is lower than around 10.8. If the p*K*_a_ value of the presence of acids is higher than this value, the product distribution switches to H_2_O_2_. Moreover, as shown in Figure S12, a more acidic proton source increases the likelihood of water forming as a product. This p*K*_a_-dependent behavior of product selectivity implies that the protonation process of **1** must play a key role in affecting product distribution from ORR. As reported before, the p*K*_a_ value for the oxime group from **1** is between 10.7 and 12.7 when the cobalt center is Co(II).^22,37^ This range is close to the switched p*K*_a_ value observed here. Therefore, we suspect that the oxime moiety can act as the relay center for the ORR via intramolecular proton transfer as HER, thereby facilitating the formation of water versus hydrogen peroxide.

To further explore the effect of electrode potential on product selectivity, we evaluated the product distribution among different acids at an overpotential of 0.23 V relative to the equilibrium potential of the HER for each acid. This overpotential was selected because the HER mediated by **1** requires approximately 0.2 V overpotential. A similar trend for product selectivity among acids was also observed (Figure S13). A combination of these RRDE findings and GC results revealed that in the presence of O_2_, ORR takes over HER as the dominant reaction and the p*K*_a_ value of acid impacts the ORR product distribution.

### Mechanistic Studies of Product Selectivity of ORR Mediated by 1

**Figure 4:**
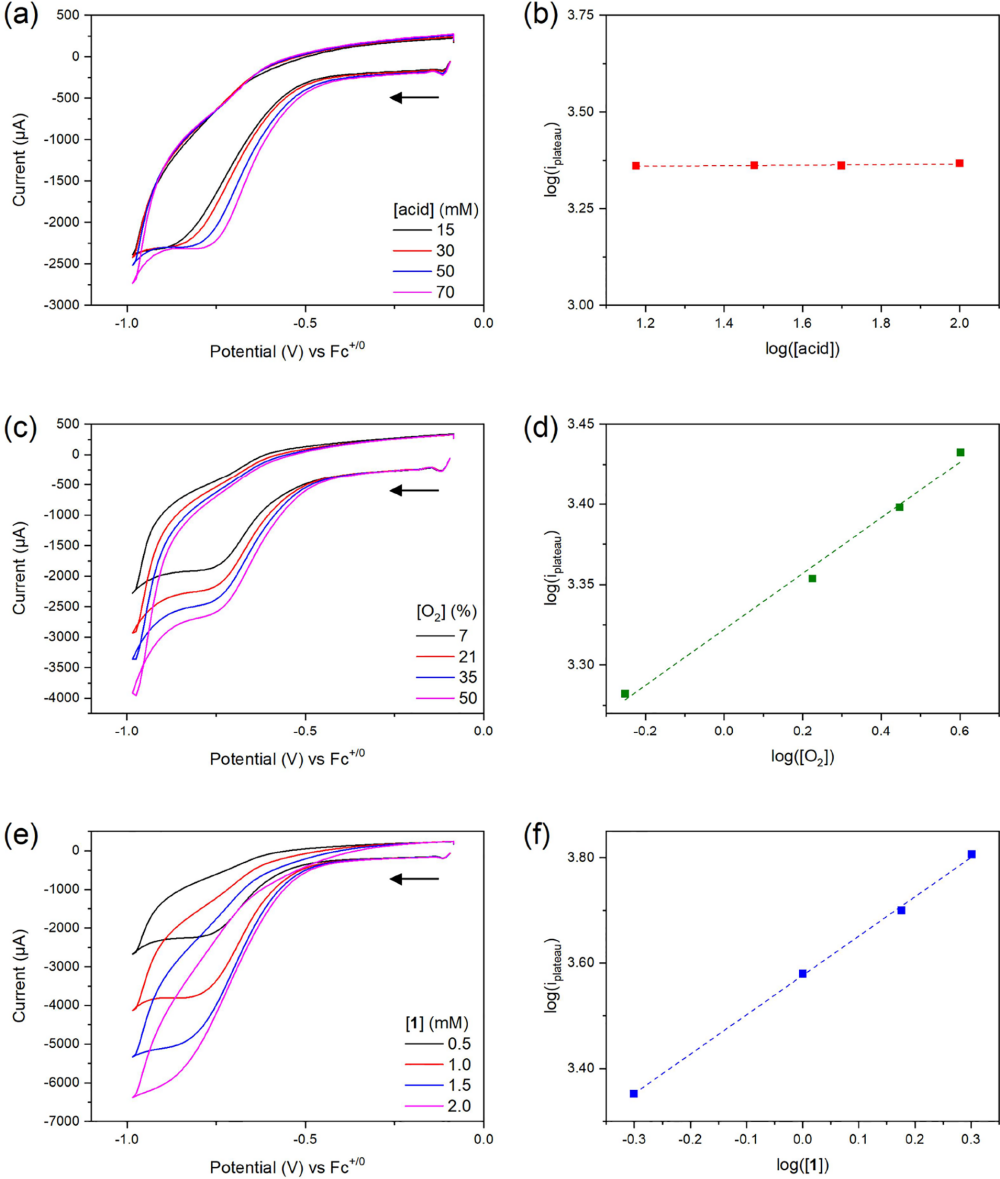
Rate law studies of ORR mechanism in the presence of *p*-cyanoanilinium (a) cyclic voltammograms of electrocatalysis by **1** (0.5 mM) in the presence of different concentrations of acid at υ = 100 V/s under 21% O_2_ and 79% N_2_. (b) The logarithm of the plateau current is plotted against the logarithm of acid concentration. (c) Different ratios of dioxygen from 7% O_2_ to 50% O_2_ balancing with N_2_. [**1**] = 0.5 mM, υ = 100 V/s, [acid] = 70 mM. (d) The logarithm of the plateau current is plotted against the logarithm of dioxygen concentration. (e) Different concentrations of **1** under 21% O_2_ and 79% N_2_. υ = 100 V/s, [acid] = 70 mM. (f) The logarithm of the plateau current is plotted against the logarithm of catalyst concentration.

The mechanism governing product selectivity in ORR mediated by **1** was further investigated by electrokinetic experiments to understand the rate law concerning the concentration of acid, molecular oxygen, and catalysts, respectively. In Figure 1b, the ORR voltammograms recorded at a scan rate of 3 V/s revealed a peak shape, indicating the occurrence of side phenomena, likely due to substrate depletion. To minimize these effects and facilitate analysis of the electrocatalytic ORR behavior promoted by **1**, a high scan rate was applied to achieve an S-shaped voltammogram.^28^ The various scan rates of collected voltammograms in the different concentrations of acids can be found in Figure S21. The potential independence of the plateau current was achieved as shown in Figure 4a when the scan rate was employed at 100 V/s, which permits further conduct of electrokinetic analysis. The plateau current for the ORR was not influenced by varying acid concentrations of *p*-cyanoanilinium under 21% O_2_ and 79% N_2_. In comparison, when the concentration of oxygen or **1** increased as shown in Figure 4c,e in the presence of 140 equiv of the acid, the plateau current increased accordingly. These results indicate the zero-order reaction with proton concentration and the first-order reaction with respect to the concentration of oxygen and molecular catalysts, respectively. These observations suggest that the formation of H_2_O from the ORR mediated by **1** is controlled by the intramolecular proton transfer through the oxime group of **1** rather than exogenous acid. Since the electrocatalytic limiting current in the presence of *p*-cyanoanilinium did not achieve scan-rate independence in our electrochemical system, this indicates that the overall rate is diffusion-limited. Therefore, the only lowest limit of TOF for ORR is estimated to be 8963 ± 33 s^–1^ according to eq 6. This lowest estimated value is at least 100 times faster than the TOF of HER in the same acid. This explains why only a trace amount of H_2_ was observed under oxygen conditions in the GC measurements. In contrast, in the presence of *p*-methoxyanilinium, the plateau current increased as the concentration of the acid increased, as shown in Figure S22. This observation suggests that the intermolecular proton transfer step is the dominant step for the formation of H_2_O_2_ in the presence of a weak acid.

Finally, the impact of the products from ORR, water, and hydrogen peroxide, on complex **1** was examined using UV–vis spectroscopy. Upon mixing H_2_O_2_ for an hour, changes were observed in the UV–vis absorption spectra of complex **1**. However, no changes occurred when only water was present (Figure S23). These results indicate that H_2_O_2_ could react with complex **1**, potentially leading to partial inactivation or damage of the complex. This may also explain why a slightly lower FE value for the HER was observed from bulk electrolysis after replacing the O_2_ with N_2_ in the presence of *p*-methoxyanilinium.

### Role of the Axial Ligands

For certain weak acids investigated here, such as the presence of *p*-methoxyanilinium, the onset potential for the ORR mediated by **1** is expected to be close to the reduction potential of the oxidized O_2_/O_2_^·–^. Therefore, in practice, it may not have a high driving force to facilitate ORR to remove O_2_ during HER due to the restraint imposed by the reduction potential of Co(III)/Co(II) from **1**. In principle, the reduction potential of this redox couple can be tuned by axial ligand.^38^ A more labile ligand is expected to lead to a more positive reduction potential for Co(III)/Co(II), thereby invoking ORR in a more positive potential and offering a higher driving force.^39^ Along this line, the cobalt diimine-dioxime complex with two labile acetonitrile, **2** as the axial ligands have been successfully prepared, and the crystal structure of **2** is shown in Figure S24a. The voltammograms of **2** in the absence of acid in Figure S24b revealed an anodically shifted redox couple peak for Co(III)/Co(II) species, compared to **1**. For the reduction potential of Co(II)/Co(I) species, two complexes reveal an identical value, suggesting the same coordination environment of the complex after the formation of Co(II) species. After introducing 21% O_2_ and 79% N_2_ in the presence of *p*-cyanoanilinium, the onset potential for ORR mediated by **2** is much more positive compared to **1** as shown in Figure S25. Further examinations of ORR product distribution mediated by **2** (Figures S14 and S15) among five acids used earlier remained the same trends of product selectivity observed from **1**. The main product is H_2_O when the presence of acid p*K*_a_ is lower than 10.8. These results suggest that ORR mediated by **2** can take place in a more positive potential compared to **1** and hence give a high driving force to remove residual O_2_ in solution during HER.

## Discussion

**Scheme 2:**
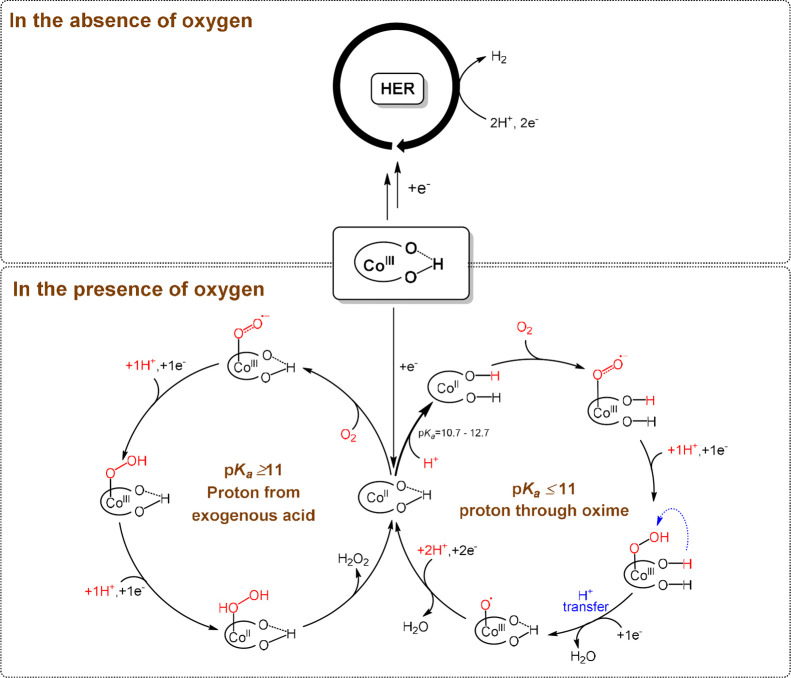
HER Mechanism of the Cobalt Diimine-Dioxime Complex in the Presence of Molecular Oxygen The p*K*_a_ range of the oxime group in Scheme 2 is obtained from ref (37).

Inspired by natural enzymes, incorporating a functional site onto SCS has been a popular approach in designing molecular electrocatalysts to facilitate energy-conversion reactions. For example, it is well-established that the oxime moiety from the cobalt diimine-dioxime complex plays a proton-relaying role in facilitating HER.^22−24^ It is intriguing whether the oxime moiety can also play a similar role in promoting the ORR, thereby being a more oxygen-tolerant electrocatalyst. As shown in Figure 1, the ORR proceeds following the reduction of the cobalt center to Co(II). The change in product distribution between H_2_O and H_2_O_2_ was observed when the p*K*_a_ value of added acid was higher than 10.9. This observation aligns with the reported p*K*_a_ value range of the oxime group between 10.7 and 12.7 when the oxidation state of the Co center is II as shown in Scheme 2.^22,37^ Protonation of the oxime group has been shown to be a crucial step in facilitating HER, as reported in previous studies.^27,37,40^ Therefore, we suspected that the protonated oxime group might also promote the ORR reaction and modulate the selectivity of ORR.

To control the product selectivity between H_2_O_2_ and H_2_O, it is known that the O–O bond-breaking step is the crucial step.^10,41^ The curve shape of the foot of wave analysis (Figures S17–S20) excludes the possibility of the chemical step (either O_2_ binding, proton transfer, or a combination of both steps) associated with the first electron transfer step for ORR as the potential rate-limiting step as shown in Scheme 2.^31,42,43^ Furthermore, the electrokinetic analysis for ORR revealed the zero-order reaction to *p*-cyanoanilinium and the first-order reaction to *p*-methoxyanilinium, suggesting an intramolecular proton transfer occurring when a strong acid is employed, likely through the oxime group. These findings suggest that the O–O bond-breaking step is probably the rate-limiting step during ORR with protonated oxime enhancing O–O bond cleavage via intramolecular proton transfer, thus favoring H_2_O formation and affecting oxygen tolerance in molecular catalysts. Based on these findings and previous studies of HER mediated by the cobalt diimine-dioxime complex, the proposed mechanism for HER in the presence of molecular oxygen is illustrated in Scheme 2.

Comparing the TOF between ORR and HER mediated by **1**, the TOF of ORR is at least 100 times faster than that of HER, and the high TOF of ORR facilitates residual O_2_ in solution to generate benign H_2_O product avoiding the formation of ROS directly from the electrode surface during HER. From the overpotential consideration of ORR mediated by **1**, the value is high compared to other cobalt complexes that mediate ORR.^10^ This is due to a more negative reduction potential of the Co(III)/(II) state. For example, the potential difference between the reduction potential of Co(III)/(II) and the equilibrium potential of ORR in the presence of *p*-cyanoanilinium is ca. 1.3 V. Nevertheless, this large value actually benefits complex **1** to facilitate ORR due to its high driving force since energy efficiency for ORR is not the main concern for the oxygen-tolerant HER electrocatalyst. To reduce this high overpotential for the ORR, the labile ligand as exemplified by **2** can be further manipulated to tune overpotential. Finally, for a metal complex to be oxygen-tolerant, the ligand itself shall not be susceptible to molecular oxygen or ROS. For example, phosphine-type ligands, such as Ni-bis(diphosphine) derivatives for their outstanding HER performance, tend to be oxygen-sensitive due to their oxyphilicity.^11^

To further understand the practical applications of HER mediated by the cobalt complex under ambient conditions, we compared our findings to those reported by the Artero group.^16^ Despite the same cobalt complex, the experimental conditions differ significantly, particularly in the solvent system (water vs acetonitrile) and complex form (homogeneous vs heterogeneous system). In the Artero group’s system, the product distribution for ORR at pH 4.5 identified water as the predominant product. Nevertheless, their electrochemical data suggested concurrent HER and ORR activity, which is opposed to our observations of a negligible HER under air-saturated conditions. This discrepancy likely arises from the higher solubility of O_2_ in acetonitrile compared to water. In our system, reducing the oxygen concentration to 7% O_2_ and 93% inert gas increased the Faradaic efficiency for HER to 16.8%. These findings indicate that under oxygen-rich conditions the higher turnover number (TON) for ORR enables the cobalt complex to initially dominate over HER. As the oxygen concentration decreases in solution due to ORR, HER gradually becomes the dominant reaction, revealing the oxygen-tolerant behavior.

## Conclusions

Here, we investigate how the role of the oxime moiety as a proton relay site impacts the oxygen-tolerant behavior of an HER electrocatalyst. The thermodynamic relationship between the Bro̷nsted acid and this site plays a crucial role in the product distribution of the ORR, consequently influencing the efficacy of oxygen-tolerant molecular catalysts. In a broader context, incorporating a proton relay site at the SCS can be regarded as an additional reaction pathway, supplementing the original reaction pathway through exogenous proton transfer routes. Therefore, understanding thermodynamic parameters to manipulate proton transfer routes by varying proton sources offers an opportunity to tailor molecular catalysts for oxygen tolerance and p*K*_a_-dependent product distribution in fuel-forming reactions.

## Experimental Section

### General Procedures and Chemicals

Unless specifically mentioned, all solvents and chemicals were used as received without further purification. Nitrogen (99.999%), Dioxygen (99.99%), pentane (Duksan, >99.9%), acetone (Duksan, 99.7%), diisopropyl ether (Acros, 99%), dichloromethane (Macron, 99.8%), methanol (Macron, 99.8%), and ethanol (J.T. Baker, 99.5%) were used directly. Acetonitrile (J.T. Baker, >99.9%) and diethyl ether (Echo, 95%) were pretreated with 3 Å molecular sieves overnight before being introduced to the solvent purification systems (Vigor Gas Purification Technologies). All electrolytes, tetrabutylammonium hexafluorophosphate (NOVA Materials, 99%), were recrystallized before use. The solvent underwent degassing through at least five freeze–pump–thaw cycles before taking into the nitrogen-filled glovebox (Vigor) and then they were stored with 3 Å molecular sieves.

For the preparation of Bro̷nsted acids, the following chemicals were employed as received: Aniline (Sigma-Aldrich, 99.5%), *p*-nitroaniline (Thermo Scientific, 99%), *p*-aminobenzonitrile (Thermo Scientific, 98%), *p*-chloroaniline (Thermo Scientific, 98%), *p*-*tert*-butylaniline (Acros Organics, 99%), *p*-anisidine (Alfa Aesar, 99%), and tetrafluoroboric acid diethyl ether complex (Alfa Aesar, 50–55%).

For the synthesis of complexes, the following chemicals were employed as received: diacetyl monoxime (TCI, 98%), 1,3-diaminopropane (Acros, 99%), cobalt(II) chloride (Alfa Aesar, >99.9%), silver nitrate (Alfa Aesar, > 99.9%), perchloric acid (SHOWA, 70%).

### Synthesis of the Ligand, Diimine-Dioxime

The procedure was initiated with a dry two-neck round-bottom flask containing a stirring bar, equipped with a Graham condenser and a Dean–Stark apparatus filled with a 3 Å molecular sieve. The whole system was baked briefly by a heat gun and placed under a vacuum. The solid, diacetyl monoxime (20.0 mmol), was introduced into the flask under a nitrogen atmosphere, and the system was evacuated for 15 min to remove water from the solid. Subsequently, 80 mL of diisopropyl ether was used to dissolve the solid, and 1,3-diaminopropane (10.0 mmol) was added to the flask under a nitrogen atmosphere. The reaction mixture was then heated to 70 °C under nitrogen, with the solvent continuously removed from the Dean–Stark apparatus until the appearance of white solid precipitates. The white solid was washed with dried diisopropyl ether at least three times to eliminate any residual reactant, followed by vacuum drying. Finally, the dried solid was recrystallized using acetonitrile. (yield: 1.70 g, 70%), ^1^H NMR (300 MHz, d_6_-DMSO) δ 11.45 (s, 2H), 3.50 (t, 4H, ^3^*J*_H–H_ = 6.6 Hz), 2.02 (s, 6H), 2.03–1.97 (m, 2H), 1.96 (s, 6H) ppm.

### Synthesis of Cobalt Diimine-Dioxime Complex

#### CoCl_2_-Diimine-Dioxime (1)

1.0 mmol of the diimine-dioxime ligand was placed in a 100 mL round-bottom flask with a stirring bar and dissolved in 20 mL of dried acetone. Then, cobalt(II) chloride, previously dissolved in 20 mL of dried acetone, was added to the flask, and the mixture was allowed to stir overnight under ambient air. The color change of the solution was observed from blue to dark green. Filter the resulting solution using a suction filter to remove solid impurities. The malachite green solid precipitated after the concentration of the solution via a rotary evaporator. Wash the solid three times with diethyl ether to remove any remaining reactants and then dry it under vacuum. Finally, the solid powder was dissolved in dichloromethane (DCM) for recrystallization, resulting in the formation of malachite crystals. (Yield: 0.26 g, 70%), ^1^H NMR (300 MHz, d_6_-DMSO) δ 19.35 (s, 1H), 4.05 (t, ^3^*J*_H–H_ = 4.3 Hz), 2.67 (s, 6H), 2.53 (s, 6H), 2.46–2.41 (m, 2H) ppm. ^13^C NMR (75 Hz, d-CDCl_3_) δ 174.1, 155.0, 50.1, 27.5, 17.7, 13.9 ppm. EA: C: 35.93% (35.99%), N, 15.07% (15.18%), H: 5.31% (5.19%).

#### Co-Diimine-Dioxime-(H_2_O)_2_-(ClO_4_)_2_

Following the procedures reported by the Petersgroup,^44^ 1.0 mmol of **1** was suspended in 10 mL of H_2_O. Subsequently, 1.9 mmol of AgNO_3_ was introduced into the solution as a solid, and the mixture was stirred in darkness for 2 h. The resulting solid impurities were removed using a filter crucible, followed by three washes with water. The filtrate was then concentrated to 5 mL under reduced pressure distillation, after which 10 mL of perchloric acid was added, and the solution was refrigerated. Brown crystals precipitated after 2 days and were stored in the fridge at 4 °C. Finally, the crystal product was rinsed with diethyl ether and dried under vacuum (yield: 0.32 g, 60%). ^1^H NMR (300 MHz, d_3_-ACN) δ 18.78 (*s*, 1H), 4.24 (t, ^3^*J*_H–H_ = 4.8 Hz), 2.88 (s, 6H), 2.79 (s, 6H), 2.42–2.35 (m, 2H) ppm.

#### Co-Diimine-Dioxime-(ACN)_2_-(ClO_4_)_2_ (2)

A small quantity of dry acetonitrile was used to dissolve the Co-diimine-dioxime-(H_2_O)_2_-(ClO_4_)_2_ solid, and the solution was stored in darkness for 2 days. Subsequently, the orange solid precipitated upon the dropwise addition of diethyl ether to the solution. The solvent was then evaporated, and the solid was further dried under vacuum. Finally, the crystal product was rinsed with diethyl ether and dried under vacuum (yield: 0.134 g, 81%). ^1^H NMR (300 MHz, d_3_-ACN) δ 18.44 (*s*, 1H), 4.16 (t, ^3^*J*_H–H_ = 4.6 Hz), 2.79 (s, 6H), 2.69 (s, 6H), 2.41–2.35 (m, 2H) ppm. ^13^C NMR (75 Hz, d_3_-ACN) δ 183.4, 161.6, 133.3, 51.4, 28.0, 19.4, 14.7, 5.2 ppm. The elemental analysis results can be found on Page S27.

#### Synthesis and Characterization of Acids

The procedures for synthesizing acids were based on the methods reported by the Dempsey group.^30^ All acids were prepared under a nitrogen atmosphere using the Schlenk line technique and conducted inside a glovebox. A Schlenk flask with a stirring bar was dried in an oven and then cooled under a vacuum before use. For solid aniline derivatives, 1.0 g of the compound was placed into the flask and subjected to a 10 min vacuum treatment to desiccate the solid. For liquid derivatives, the vacuum treatment was omitted.

Next, 5 mL of dried diethyl ether was added to dissolve the solids. Upon complete dissolution, a solution containing 0.95 mol equiv of tetrafluoroboric acid diethyl ether complex was added dropwise within an ice bath, inducing the precipitation of the tetrafluoroborate salt. After a 10 min reaction, the Schlenk line was used to evaporate all solvent and dry the acid solid. Subsequent procedures were conducted in a glovebox.

The solid was rinsed with 5 mL of diethyl ether at least three times to remove residual starting materials, with the ether subsequently removed via a vacuum. For acid recrystallization, the solid was first dissolved in acetonitrile and then added dropwise to a Schlenk flask containing 20 mL of diethyl ether. The flask was placed in a refrigerator at −35 °C overnight, leading to the crystallization of the acid. Following solvent evaporation, the crystals were washed with pentane and ether and then vacuum-dried.

##### *p*-Cyanoanilinium

Yield: 0.96 g, 58%. ^1^H NMR (300 MHz, d_3_-ACN) δ 8.37 (br, 3H), 7.91 (d, ^3^*J*_H–H_ = 8.6 Hz, 2H), 7.61 (d, ^3^*J*_H–H_ = 8.6 Hz, 2H) ppm.

##### *p*-Chloroanilinium

Yield: 0.88 g, 57%. ^1^H NMR (300 MHz, d_3_-ACN) δ 8.18 (br, 3H), 7.57 (d, ^3^*J*_H–H_ = 8.4 Hz, 2H), 7.42 (d, ^3^*J*_H–H_ = 8.4 Hz, 2H) ppm.

##### Anilinium

Yield: 3.90 g, 70%. ^1^H NMR (300 MHz, d_3_-ACN) δ 8.17 (br, 3H), 7.56–7.54 (m, 3H), 7.46–7.43 (m, 2H) ppm.

##### *p*-*tert*-Butylanilinium

Yield: 0.88 g, 58%. ^1^H NMR (300 MHz, d_3_-ACN) δ 8.01 (br, 3H), 7.59 (d, ^3^*J*_H–H_ = 8.5 Hz, 2H), 7.35 (d, ^3^*J*_H–H_ = 8.5 Hz, 2H), 1.34 (s, 9H) ppm.

##### *p*-Methoxyanilinium

Yield: 0.88 g, 54%. ^1^H NMR (300 MHz, d_3_-ACN) δ 8.00 (br, 3H), 7.34 (d, ^3^*J*_H–H_ = 8.6 Hz, 2H), 7.06 (d, ^3^*J*_H–H_ = 8.6 Hz, 2H), 3.84 (s, 3H) ppm.

#### Stationary Electrochemical Experiments

The electrochemical measurements were acquired using a Metrohm Autolab—PGSTAT302N system. For the stationary system, a glassy carbon electrode (3 mm diameter, CH instrument) was used as a working electrode, and a platinum wire was used as a counter electrode. The reference electrode was prepared via a silver wire immersed in acetonitrile containing 0.01 M silver nitrate and 0.1 M TBAPF_6_. The IR correction method was implemented based on the approach described in the literature.^45^ Before the high-speed scan rate measurements were performed, the solvent resistance was measured using electrochemical impedance spectroscopy (EIS) (conducted by a Metrohm potentiostat equipped with the FRA32 M module). The value of solution resistance obtained from EIS was used to perform positive feedback measurement to determine the uncompensated resistance value, *R*_u_. This value is used for the IR compensation settings during measurements.

#### Bulk Electrolysis

Bulk electrolysis was carried out in an H-cell divided into two compartments by a glass frit. Each compartment contained 10 mL of dry acetonitrile with 0.1 M TBAPF_6_. Complex **1** (0.5 mM) and an acid (50 mM) were added to both sides of the H-cell. Two glassy carbon plates with an electrode area submerged in the solution, 1.5 × 1.0 cm^2^, were employed as the working and counter electrodes respectively. The reference electrode was a silver wire immersed in acetonitrile containing 0.01 M silver nitrate and 0.1 M TBAPF_6_. The electrolysis potential was set at −1.2 V (vs Fc^+/0^) with continuous stirring of the solution. After electrolysis, the concentration of H_2_ was measured using a Shimadzu Nexis GC-2010 gas chromatograph equipped with a Micropacked ST column (2.0 m × 1.0 mm ID). Helium (99.9999% purity) was used as the carrier gas at a flow rate of 300 kPa. A barrier discharge ionization detector was employed for product analysis. Following bulk electrolysis, a 300 μL sample of the cell’s headspace gas was collected using a Hamilton gastight syringe. The same experimental conditions were repeated at least twice.

#### Rotating Ring-Disk Electrode

For rotation-ring-disk-electrode (RRDE) measurements, the Pine Research AFMSRCE system was utilized and operated in dry acetonitrile. The glassy carbon was used as the disk electrode, with platinum as the ring electrode. The reference electrode was a silver wire immersed in acetonitrile containing 0.01 M silver nitrate and 0.1 M TBAPF_6_. During all RRDE voltammetry experiments, the potential of the disk electrode was swept from 0 to −1.3 V at a scan rate of 10 mV/s. The ring electrode was maintained at 0.45 V to measure the formation of hydrogen peroxide generated by the catalysts. The rotation rates were employed at 1500 rpm. The acid concentration is 50 mM in all electrochemical experiments conducted here. The same experimental conditions were repeated at least twice.

All electrochemical measurements under various gas ratios were prepared using two mass flow controllers (Sierra-Smart Track 100). The gas mixture was first passed through a gas bubbler filled with dry ACN before reaching the electrochemical cell. Each gas composition was purged for 15 min before conducting electrochemical measurements.

## Supporting Information Available

Detailed characterization data for complex **1**, **2**, and acids; complementary electrochemical data and electrokinetic analysis (PDF)

## References

[ref1] Dalle K. E.; Warnan J.; Leung J. J.; Reuillard B.; Karmel I. S.; Reisner E. Electro- and Solar-Driven Fuel Synthesis with First Row Transition Metal Complexes. Chem. Rev. 2019, 119, 2752–2875. 10.1021/acs.chemrev.8b00392. 30767519 PMC6396143

[ref2] Cobb S. J.; Rodríguez-Jiménez S.; Reisner E. Connecting Biological and Synthetic Approaches for Electrocatalytic CO_2_ Reduction. Angew. Chem., Int. Ed. 2024, 63, e202310547 10.1002/anie.202310547. PMC11497245 37983571

[ref3] Drover M. W. A guide to secondary coordination sphere editing. Chem. Soc. Rev. 2022, 51, 1861–1880. 10.1039/D2CS00022A. 35188514

[ref4] Wiedner E. S.; Appel A. M.; Raugei S.; Shaw W. J.; Bullock R. M. Molecular Catalysts with Diphosphine Ligands Containing Pendant Amines. Chem. Rev. 2022, 122, 12427–12474. 10.1021/acs.chemrev.1c01001. 35640056

[ref5] Bhunia S.; Ghatak A.; Dey A. Second Sphere Effects on Oxygen Reduction and Peroxide Activation by Mononuclear Iron Porphyrins and Related Systems. Chem. Rev. 2022, 122, 12370–12426. 10.1021/acs.chemrev.1c01021. 35404575

[ref6] Savéant J.-M. Proton Relays in Molecular Catalysis of Electrochemical Reactions: Origin and Limitations of the Boosting Effect. Angew. Chem., Int. Ed. 2019, 58, 2125–2128. 10.1002/anie.201812375. 30548762

[ref7] Ahmed M. E.; Dey S.; Darensbourg M. Y.; Dey A. Oxygen-Tolerant H_2_ Production by [FeFe]-H_2_ase Active Site Mimics Aided by Second Sphere Proton Shuttle. J. Am. Chem. Soc. 2018, 140, 12457–12468. 10.1021/jacs.8b05983. 30180564

[ref8] Mondal B.; Sen P.; Rana A.; Saha D.; Das P.; Dey A. Reduction of CO_2_ to CO by an Iron Porphyrin Catalyst in the Presence of Oxygen. ACS Catal. 2019, 9, 3895–3899. 10.1021/acscatal.9b00529.

[ref9] Wang V. C. C.; Esmieu C.; Redman H. J. A.; Joel W.; Berggren G.; Hammarström L. The reactivity of molecular oxygen and reactive oxygen species with [FeFe] hydrogenase biomimetics: reversibility and the role of the second coordination sphere. Dalton Trans. 2020, 49, 858–865. 10.1039/C9DT04618F. 31854399

[ref10] Pegis M. L.; Wise C. F.; Martin D. J.; Mayer J. M. Oxygen Reduction by Homogeneous Molecular Catalysts and Electrocatalysts. Chem. Rev. 2018, 118, 2340–2391. 10.1021/acs.chemrev.7b00542. 29406708

[ref11] Yang J. Y.; Bullock R. M.; Dougherty W. G.; Kassel W. S.; Twamley B.; DuBois D. L.; Rakowski DuBois M. Reduction of oxygen catalyzed by nickel diphosphine complexes with positioned pendant amines. Dalton Trans. 2010, 39, 3001–3010. 10.1039/b921245k. 20221533

[ref12] Wakerley D. W.; Gross M. A.; Reisner E. Proton reduction by molecular catalysts in water under demanding atmospheres. Chem. Commun. 2014, 50, 15995–15998. 10.1039/C4CC06159D. 25407336

[ref13] Borup R.; Meyers J.; Pivovar B.; Kim Y. S.; Mukundan R.; Garland N.; Myers D.; Wilson M.; Garzon F.; Wood D.; Zelenay P.; More K.; Stroh K.; Zawodzinski T.; Boncella J.; McGrath J. E.; Inaba M.; Miyatake K.; Hori M.; Ota K.; Ogumi Z.; Miyata S.; Nishikata A.; Siroma Z.; Uchimoto Y.; Yasuda K.; Kimijima K.-I.; Iwashita N. Scientific Aspects of Polymer Electrolyte Fuel Cell Durability and Degradation. Chem. Rev. 2007, 107, 3904–3951. 10.1021/cr050182l. 17850115

[ref14] Kleingardner J. G.; Kandemir B.; Bren K. L. Hydrogen Evolution from Neutral Water under Aerobic Conditions Catalyzed by Cobalt Microperoxidase-11. J. Am. Chem. Soc. 2014, 136, 4–7. 10.1021/ja406818h. 24351231

[ref15] Mondal B.; Sengupta K.; Rana A.; Mahammed A.; Botoshansky M.; Dey S. G.; Gross Z.; Dey A. Cobalt Corrole Catalyst for Efficient Hydrogen Evolution Reaction from H_2_O under Ambient Conditions: Reactivity, Spectroscopy, and Density Functional Theory Calculations. Inorg. Chem. 2013, 52, 3381–3387. 10.1021/ic4000473. 23445187

[ref16] Kaeffer N.; Morozan A.; Artero V. Oxygen Tolerance of a Molecular Engineered Cathode for Hydrogen Evolution Based on a Cobalt Diimine–Dioxime Catalyst. J. Phys. Chem. B 2015, 119, 13707–13713. 10.1021/acs.jpcb.5b03136. 25993343

[ref17] Lakadamyali F.; Kato M.; Muresan N. M.; Reisner E. Selective Reduction of Aqueous Protons to Hydrogen with a Synthetic Cobaloxime Catalyst in the Presence of Atmospheric Oxygen. Angew. Chem., Int. Ed. 2012, 51, 9381–9384. 10.1002/anie.201204180. 22915369

[ref18] Teindl K.; Patrick B. O.; Nichols E. M. Linear Free Energy Relationships and Transition State Analysis of CO_2_ Reduction Catalysts Bearing Second Coordination Spheres with Tunable Acidity. J. Am. Chem. Soc. 2023, 145, 17176–17186. 10.1021/jacs.3c03919. 37499125

[ref19] Sonea A.; Crudo N. R.; Warren J. J. Understanding the Interplay of the Bro̷nsted Acidity of Catalyst Ancillary Groups and the Solution Components in Iron-porphyrin-Mediated Carbon Dioxide Reduction. J. Am. Chem. Soc. 2024, 146, 3721–3731. 10.1021/jacs.3c10127. 38307036

[ref20] Krumova K.; Cosa G. Overview of Reactive Oxygen Species. In Singlet Oxygen: Applications in Biosciences and Nanosciences; Nonell S.; Flors C.; Nonell S.; Flors C., Eds.; The Royal Society of Chemistry, 2016.

[ref21] Li M.; Wang H.; Luo W.; Sherrell P. C.; Chen J.; Yang J. Heterogeneous Single-Atom Catalysts for Electrochemical CO2 Reduction Reaction. Adv. Mater. 2020, 32, 2001848 10.1002/adma.202001848. 32644259

[ref22] Jacques P.-A.; Artero V.; Pécaut J.; Fontecave M. Cobalt and nickel diimine-dioxime complexes as molecular electrocatalysts for hydrogen evolution with low overvoltages. Proc. Natl. Acad. Sci. U.S.A. 2009, 106, 20627–20632. 10.1073/pnas.0907775106. 19948953 PMC2791621

[ref23] Dongyue S.; Aparna K. H.; Jacques P.; Sharon H.-S.; Cyrille C.; Artero V. Hydrogen Evolution Mediated by Cobalt Diimine-Dioxime Complexes: Insights into the Role of the Ligand Acid/Base Functionalities. ChemElectroChem 2021, 8, 2671–2679. 10.1002/celc.202100413.

[ref24] Kaeffer N.; Chavarot-Kerlidou M.; Artero V. Hydrogen Evolution Catalyzed by Cobalt Diimine–Dioxime Complexes. Acc. Chem. Res. 2015, 48, 1286–1295. 10.1021/acs.accounts.5b00058. 25941953 PMC4491805

[ref25] Andreiadis E. S.; Jacques P.-A.; Tran P. D.; Leyris A.; Chavarot-Kerlidou M.; Jousselme B.; Matheron M.; Pécaut J.; Palacin S.; Fontecave M.; Artero V. Molecular engineering of a cobalt-based electrocatalytic nanomaterial for H_2_ evolution under fully aqueous conditions. Nat. Chem. 2013, 5, 48–53. 10.1038/nchem.1481. 23247177

[ref26] Kaeffer N.; Massin J.; Lebrun C.; Renault O.; Chavarot-Kerlidou M.; Artero V. Covalent Design for Dye-Sensitized H_2_-Evolving Photocathodes Based on a Cobalt Diimine–Dioxime Catalyst. J. Am. Chem. Soc. 2016, 138, 12308–12311. 10.1021/jacs.6b05865. 27595317 PMC5490783

[ref27] Sun D.; Karippara Harshan A.; Pécaut J.; Hammes-Schiffer S.; Costentin C.; Artero V. Hydrogen Evolution Mediated by Cobalt Diimine-Dioxime Complexes: Insights into the Role of the Ligand Acid/Base Functionalities. ChemElectroChem. 2021, 8, 2671–2679. 10.1002/celc.202100413.

[ref28] Savéant J.-M.; Costentin C. Molecular Catalysis of Electrochemical Reactions. In Elements of Molecular and Biomolecular Electrochemistry, 2nd edition; John Wiley & Sons, 2019; pp 285–381.

[ref29] Costentin C.; Drouet S.; Robert M.; Savéant J.-M. Turnover Numbers, Turnover Frequencies, and Overpotential in Molecular Catalysis of Electrochemical Reactions. Cyclic Voltammetry and Preparative-Scale Electrolysis. J. Am. Chem. Soc. 2012, 134, 11235–11242. 10.1021/ja303560c. 22670885

[ref30] McCarthy B. D.; Martin D. J.; Rountree E. S.; Ullman A. C.; Dempsey J. L. Electrochemical Reduction of Bro̷nsted Acids by Glassy Carbon in Acetonitrile—Implications for Electrocatalytic Hydrogen Evolution. Inorg. Chem. 2014, 53, 8350–8361. 10.1021/ic500770k. 25076140

[ref31] Wang V. C. C.; Johnson B. A. Interpreting the Electrocatalytic Voltammetry of Homogeneous Catalysts by the Foot of the Wave Analysis and Its Wider Implications. ACS Catal. 2019, 9, 7109–7123. 10.1021/acscatal.9b00850.

[ref32] Achord J. M.; Hussey C. L. Determination of dissolved oxygen in nonaqueous electrochemical solvents. Anal. Chem. 1980, 52, 601–602. 10.1021/ac50053a061.

[ref33] Xing W.; Yin M.; Lv Q.; Hu Y.; Liu C.; Zhang J. 1 - Oxygen Solubility, Diffusion Coefficient, and Solution Viscosity. In Rotating Electrode Methods and Oxygen Reduction Electrocatalysts; Xing W.; Yin G.; Zhang J., Eds.; Elsevier, 2014; pp 1–31.

[ref34] Kaeffer N.; Morozan A.; Fize J.; Martinez E.; Guetaz L.; Artero V. The Dark Side of Molecular Catalysis: Diimine–Dioxime Cobalt Complexes Are Not the Actual Hydrogen Evolution Electrocatalyst in Acidic Aqueous Solutions. ACS Catal. 2016, 6, 3727–3737. 10.1021/acscatal.6b00378.

[ref35] Zhou R.; Zheng Y.; Jaroniec M.; Qiao S.-Z. Determination of the Electron Transfer Number for the Oxygen Reduction Reaction: From Theory to Experiment. ACS Catal. 2016, 6, 4720–4728. 10.1021/acscatal.6b01581.

[ref36] Gospodinova N.; Terlemezyan L. Conducting polymers prepared by oxidative polymerization: polyaniline. Prog. Polym. Sci. 1998, 23, 1443–1484. 10.1016/S0079-6700(98)00008-2.

[ref37] Solis B. H.; Yu Y.; Hammes-Schiffer S. Effects of Ligand Modification and Protonation on Metal Oxime Hydrogen Evolution Electrocatalysts. Inorg. Chem. 2013, 52, 6994–6999. 10.1021/ic400490y. 23701462

[ref38] Lever A. B. P. Electrochemical parametrization of metal complex redox potentials, using the ruthenium(III)/ruthenium(II) couple to generate a ligand electrochemical series. Inorg. Chem. 1990, 29, 1271–1285. 10.1021/ic00331a030.

[ref39] Costentin C.; Savéant J.-M. Homogeneous Molecular Catalysis of Electrochemical Reactions: Manipulating Intrinsic and Operational Factors for Catalyst Improvement. J. Am. Chem. Soc. 2018, 140, 16669–16675. 10.1021/jacs.8b09154. 30392356

[ref40] Bhattacharjee A.; Andreiadis E. S.; Chavarot-Kerlidou M.; Fontecave M.; Field M. J.; Artero V. A Computational Study of the Mechanism of Hydrogen Evolution by Cobalt(Diimine-Dioxime) Catalysts. Chem.—Eur. J. 2013, 19, 15166–15174. 10.1002/chem.201301860. 24105795

[ref41] Huang X.; Groves J. T. Oxygen Activation and Radical Transformations in Heme Proteins and Metalloporphyrins. Chem. Rev. 2018, 118, 2491–2553. 10.1021/acs.chemrev.7b00373. 29286645 PMC5855008

[ref42] Wang V. C. C. Beyond the Active Site: Mechanistic Investigations of the Role of the Secondary Coordination Sphere and Beyond in Multi-electron Electrocatalytic Reactions. ACS Catal. 2021, 11, 8292–8303. 10.1021/acscatal.0c04770.

[ref43] Passard G.; Dogutan D. K.; Qiu M.; Costentin C.; Nocera D. G. Oxygen Reduction Reaction Promoted by Manganese Porphyrins. ACS Catal. 2018, 8, 8671–8679. 10.1021/acscatal.8b01944.

[ref44] McCrory C. C. L.; Uyeda C.; Peters J. C. Electrocatalytic Hydrogen Evolution in Acidic Water with Molecular Cobalt Tetraazamacrocycles. J. Am. Chem. Soc. 2012, 134, 3164–3170. 10.1021/ja210661k. 22280515

[ref45] Azcarate I.; Costentin C.; Robert M.; Savéant J.-M. Through-Space Charge Interaction Substituent Effects in Molecular Catalysis Leading to the Design of the Most Efficient Catalyst of CO_2_-to-CO Electrochemical Conversion. J. Am. Chem. Soc. 2016, 138, 16639–16644. 10.1021/jacs.6b07014. 27976580

[ref46] Tsai Y.-S.; Chen Y.-W.; Dayawansa C. L.; Chang H.; Chen W.-C.; Shen J.-S. Mechanistic Investigations of a Hydrogen-Evolving Cobalt Diimine-Dioxime Complex in an Oxygen Environment: Roles of Secondary Coordination Sphere, Bro̷nsted Acid, and Axial Ligand. 2024, ChemRxiv. 10.1021/acs.inorgchem.4c03301 PMC11898077 39999341

