# Supplementary material for: Mechanistic Investigations of a Hydrogen-Evolving Cobalt Diimine-Dioxime Complex in an Oxygen Environment: Roles of Secondary Coordination Sphere, Bro̷nsted Acid, and Axial Ligand
Source: Inorg Chem. 2025 Feb 25;64(9):4213–22. doi: 10.1021/acs.inorgchem.4c03301 (PMC11898077; doi:10.1021/acs.inorgchem.4c03301)
Supplement: Supplementary file 1 — ic4c03301_si_001.pdf [file ic4c03301_si_001.pdf]

Supporting Information

Mechanistic Investigations of a Hydrogen-Evolving Cobalt Diimine-Dioxime Complex in an Oxygen Environment: Roles of Secondary Coordination Sphere, Brønsted Acid, and Axial Ligand

Yu-Syuan Tsai[a], Yu-Wei Chen[a], Charasee Laddika Dayawansa[a], Hsuan Chang[a], Wen-Ching Chen[b], Jiun-Shian Shen[b], Tiow-Gan Ong[b], Glenn P. A. Yap[c] and Vincent C.-C. Wang[a, d*]

a. Department of Chemistry, National Sun Yat-sen University, Kaohsiung, Taiwan 80424, R.O.C.

b. Institute of Chemistry, Academia Sinica, Taipei, Taiwan 11529, R.O.C.

c. Department of Chemistry and Biochemistry, University of Delaware, Newark, Delaware 19716, United States.

d. Green Hydrogen Research Center, National Sun Yat-sen University, Kaohsiung, Taiwan 80424, R.O.C.

Email: vincent.wang@mail.nsysu.edu.tw/vincentwang64@yahoo.com

Table of Contents

Electrochemical Experiments..2

Turnover frequency (TOF) calculation 3

Bulk electrolysis...4

The Product Selectivity of ORR...13

Discussion of the pK_a-potential relationship 20

The Foot of the Wave Analysis (FOWA) 21

The Determination of ORR Reaction Rate Law 24

UV-vis Spectroscopy...26

The Role of Axis Ligand 27

Control experiments..30

RRDE Data 31

Supplementary Figures...41

References 57

Table S1. The formal reduction potential of ORR, HER, and superoxide reactions in acetonitrile.[1, 2]

O_2+4e$^-$+4H$^+$⇌2H_2O	E=1.21−0.0592pK_a(HA)	(a)
O_2+2e$^-$+2H$^+$⇌2H_2O_2	E=0.68−0.0592pK_a(HA)	(b)
2e$^-$+$H^+_{2(ACN)}$⇌$H_{2(g)}$	E=−0.028 V	(c)
$O_{2(g)}$+e$^-$⇌$O_2^{\cdot-}$	E=−1.28 V	(d)

Electrochemical Experiments

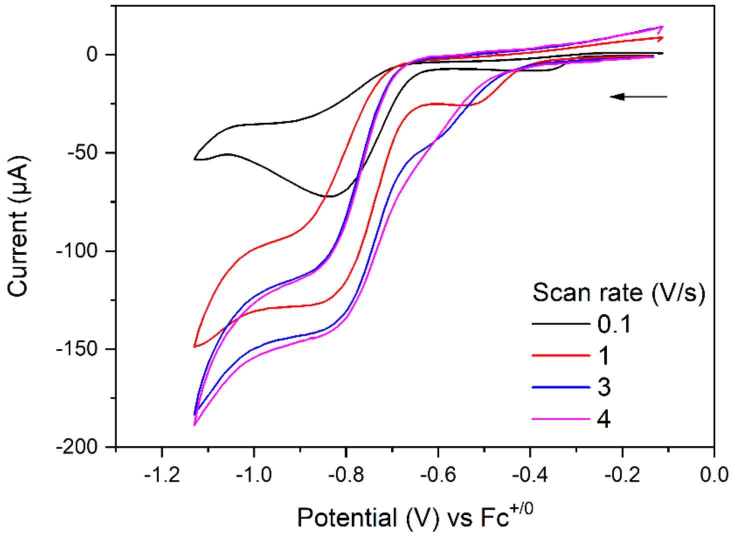

Figure S1. The electrocatalytic voltammograms of **1** (0.5 mM) in the presence of 10 equivalents of *p*-cyanoanilinium tetrafluoroborate under 100% N_2 with different scan rates.

Turnover frequency (TOF) calculation

According to reference 3 and 4, when limiting currents are achieved, the relation between the limiting current and the observed rate constant can be described by the following equation:

$$i_{lim} = nFSC\sqrt{D_{cat} \cdot k_{obs}} \qquad \text{Eq S1}$$

To avoid the calculations of electrode surface area, equation S1 was divided by the Randles-Ševčík equation to yield the following equation:

$$\frac{i_{lim}}{i_p} = \frac{nFSC\sqrt{D_{cat} \cdot k_{obs}}}{0.446FSC\sqrt{\frac{Fv}{RT}D_{cat}}} = 2.24n\sqrt{\frac{RTk_{obs}}{Fv}} \qquad \text{Eq S2}$$

i_{lim}: the limiting current obtained in the presence of substrate. For the HER mediated by cobalt diimine-dioxime, the electrocatalytic voltammogram in Figure S1 shows an S-shaped curve that is independent of scan rate. Therefore, the limiting current can be estimated in the region where the electrocatalytic current remains constant as varying potential.

i_p: the peak current was obtained in the absence of substrate under insert atmosphere. The peak height of the Co(II)/(I) couple (i_p) was determined by calculating the difference between the peak cathodic current and the baseline current preceding the Co(II)/(I) couple.

n: the number of electrons involved with the given reaction. In this case, n is equal to 2. R: ideal gas constant. T: temperature (Kelvin). F: Faraday constant. v: scan rate (V/sec). k_{obs}: pseudo-first-order rate constant (i.e. TOF, s^{-1})

Bulk electrolysis

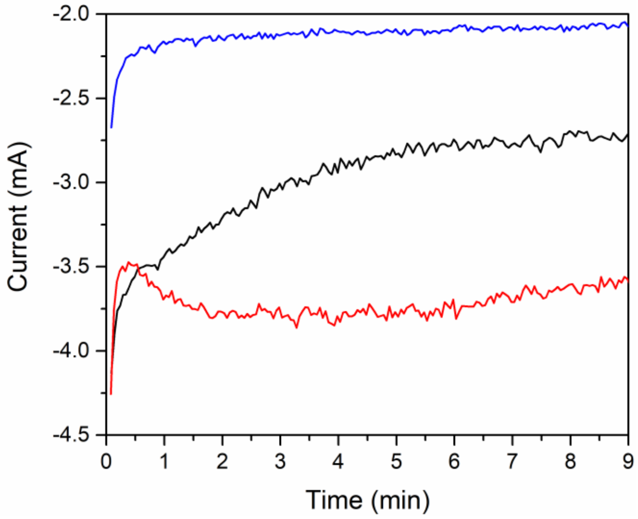

Figure S2. Chronoamperograms recorded for **1** (0.5 mM) with 100 equivalents of *p*-cyanoanilinium under various gas compositions, applying a potential of −1.2 V (vs. $Fc^{+/0}$) for 9 minutes. The black trace: under 100% nitrogen. The red trace: under a gas mixture of 79% nitrogen and 21% oxygen. The blue trace: the system purged with 100% nitrogen again following electrolysis under the 79% nitrogen and 21% oxygen atmosphere.

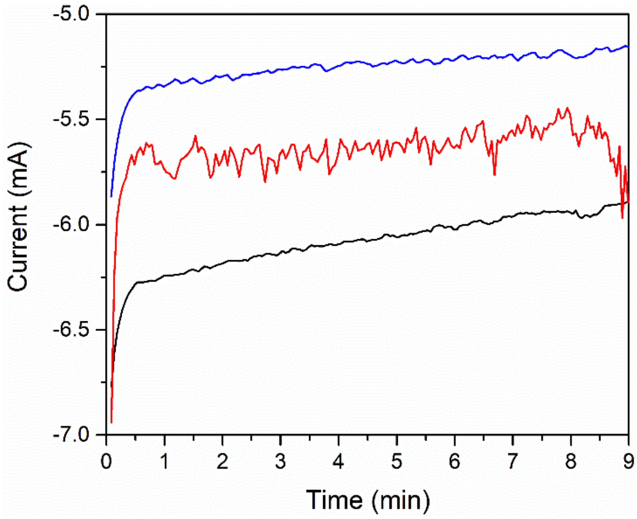

Figure S3. Chronoamperograms recorded for **1** (0.5 mM) with 100 equivalents of *p*-methoxyanilinium under various gas conditions, applying a potential of −1.2 V (vs. $Fc^{+/0}$) for 9 minutes. The black trace: under 100% nitrogen. The red trace: under a gas mixture of 79% nitrogen and 21% oxygen. The blue trace: the system purged with 100% nitrogen again following electrolysis under the 79% nitrogen and 21% oxygen atmosphere.

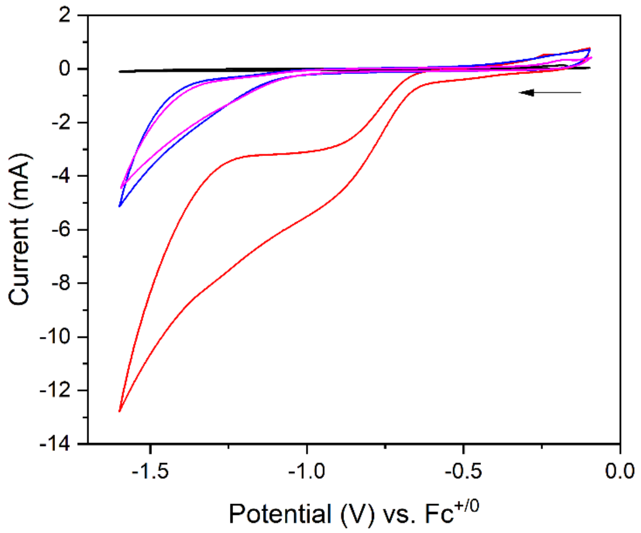

Figure S4. Rinse test experiments after electrolysis in the presence of *p*-cyanoanilinium in acetonitrile under nitrogen. The black line: only ACN solution. The Red line: **1** (0.5 mM) in the presence of 100 equivalents of *p*-cyanoanilinium before electrolysis experiments under N_2. Blue line: after the electrolysis experiment, the glassy carbon electrodes were rinsed with acetonitrile and placed into a fresh solution containing 100 equivalents of *p*-cyanoanilinium without **1** under N_2. The Magenta line: the cyclic voltammetry of *p*-cyanoanilinium (50 mM) alone using a fresh glassy carbon electrode. scan rate:0.1 V/sec.

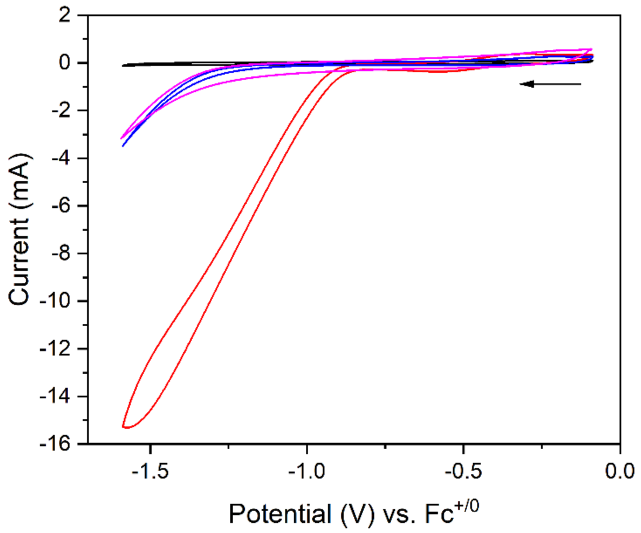

Figure S5. Rinse test experiments after electrolysis in the presence of *p*-methoxyanilinium in acetonitrile under nitrogen. The black line: only ACN solution. The Red line: **1** (0.5 mM) in the presence of 100 equivalents of *p*-methoxyanilinium before electrolysis experiments under N_2. Blue line: after the electrolysis experiment, the glassy carbon electrodes were rinsed with acetonitrile and placed into a fresh solution containing 100 equivalents of *p*-methoxyanilinium without **1** under N_2. The Magenta line: the cyclic voltammetry of *p*-methoxyanilinium (50 mM) using a fresh glassy carbon electrode. scan rate:0.1 V/s.

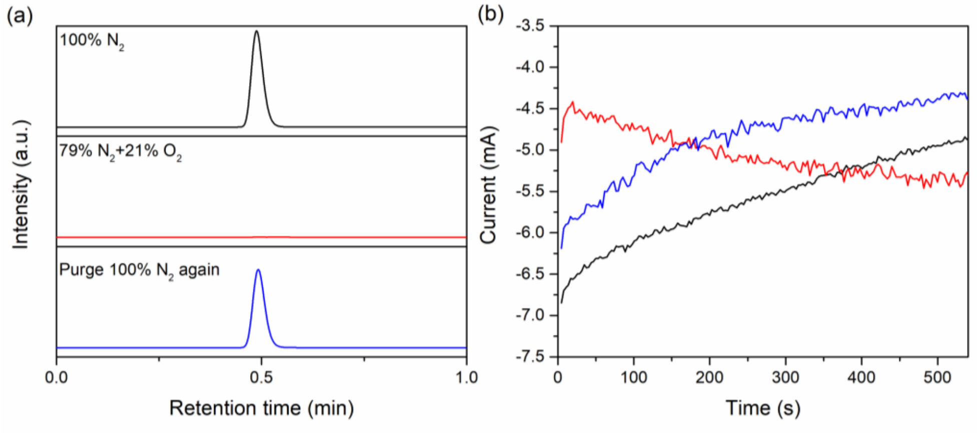

Figure S6. The GC results for bulk electrolysis of **1** (0.5 mM) with *p*-cyanoanilinium (50 mM) in acetonitrile under various gas compositions, and the working electrode was polished after every electrolysis. The electrolyzing potential was applied at −1.2 V vs $Fc^{+/0}$ for 9 minutes. (b) corresponding chronoamperograms recorded. The black trace: under 100% nitrogen. The red trace: under a gas mixture of 79% nitrogen and 21% oxygen. The blue trace: the system purged with 100% nitrogen again following electrolysis under the 79% nitrogen and 21% oxygen atmosphere.

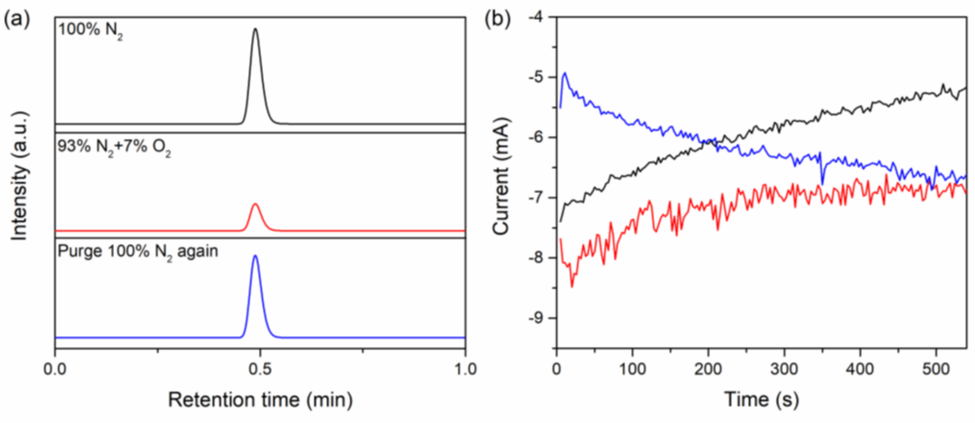

Figure S7. The GC results for bulk electrolysis of **1** (0.5 mM) with *p*-cyanoanilinium (50 mM) in acetonitrile under various gas compositions. The electrolyzing potential was applied at −1.2 V vs $Fc^{+/0}$ for 9 minutes. (b) corresponding chronoamperograms recorded. The black trace: under 100% nitrogen. The red trace: under a gas mixture of 93% nitrogen and 7% oxygen. The blue trace: the system purged with 100% nitrogen again following electrolysis under the 93% nitrogen and 7% oxygen atmosphere.

Cyclability

To assess cyclability, the electrolysis experiment was repeated over multiple N_2/O_2 cycles under the same conditions as the previous bulk electrolysis. The figure below shows the Faradaic efficiency for H_2 formation across different gas environments using *p*-cyanoanilinium as the proton source. Results indicate a slight decline in Faradaic efficiency with each successive cycle.

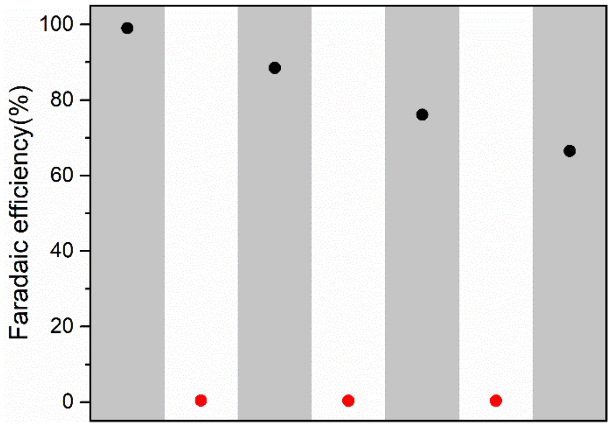

Figure S8. The cyclability test of Faradaic efficiency for the hydrogen evolution reaction catalyzed by **1** (0.5 mM) under different gas compositions, using *p*-cyanoanilinium (50 mM) as the proton source. Grey area: in the presence of 100% N_2. White area: in the presence of 79 % N_2 and 21% O_2.

Long-time electrolysis

To evaluate the HER recovery during prolonged electrolysis, a total 90-minute electrolysis experiment was conducted with 30 minutes for each gas composition. Results demonstrate that, following prolonged electrolysis, a Faradaic efficiency dropped from 99% to 84% after re-purging the system with nitrogen. The rinse test results (Figure S10) indicate that no electrodeposited species contribute to the hydrogen evolution reaction.

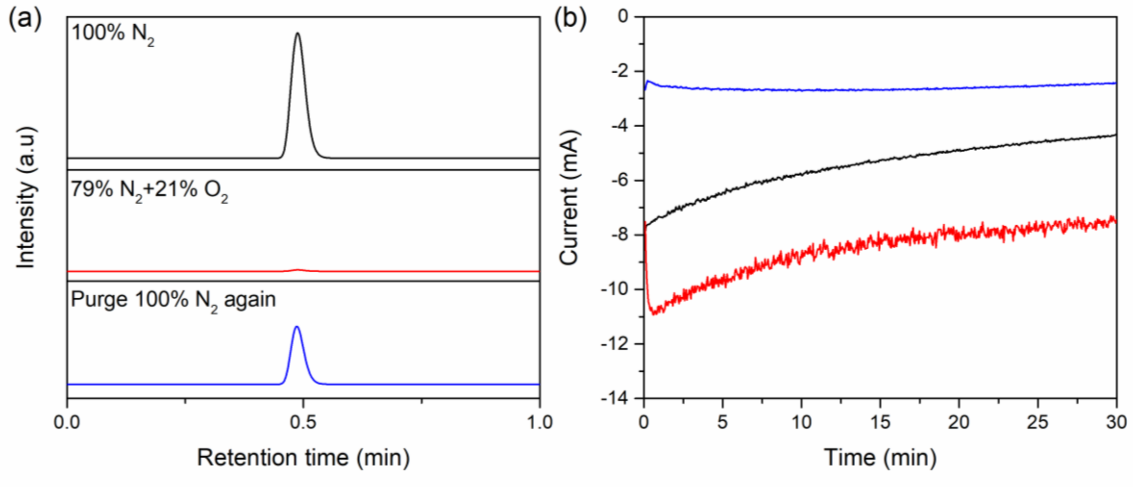

Figure S9. (a) The GC results for bulk electrolysis of **1** (0.5 mM) with *p*-cyanoanilinium (50 mM) in acetonitrile under various gas compositions. The electrolyzing potential was applied at −1.2 V vs $Fc^{+/0}$ for 30 minutes. (b) corresponding chronoamperograms recorded. The black trace: under 100% nitrogen. The red trace: under a gas mixture of 79% nitrogen and 21% oxygen. The blue trace: the system purged with 100% nitrogen again following electrolysis under the 79% nitrogen and 21% oxygen atmosphere.

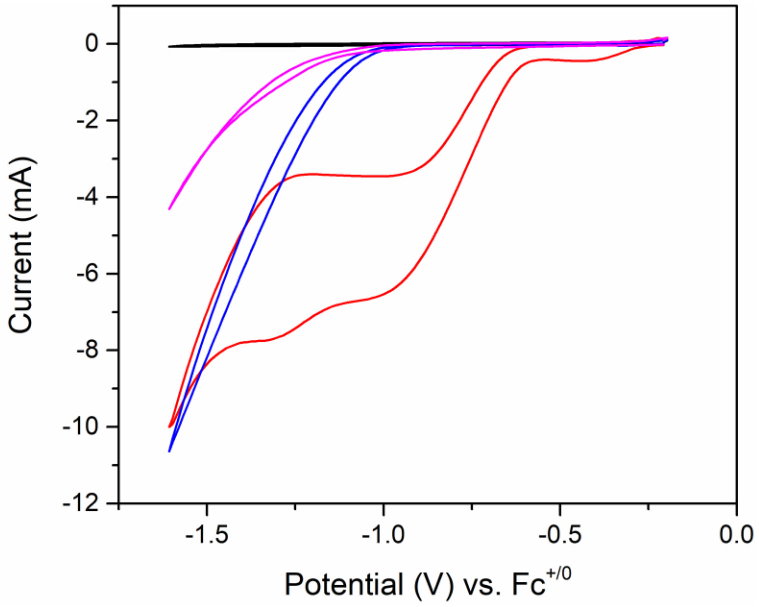

Figure S10. Rinse test experiments after 90-minute electrolysis in the presence of *p*-cyanoanilinium in acetonitrile under nitrogen. The black line: only ACN solution. The Red line: **1** (0.5 mM) in the presence of 100 equivalents of *p*-cyanoanilinium before electrolysis experiments under N_2. Blue line: after the electrolysis experiment, the glassy carbon electrodes were rinsed with acetonitrile and placed into a fresh solution containing 100 equivalents of *p*-cyanoanilinium without **1** under N_2. The Magenta line: the cyclic voltammetry of *p*-cyanoanilinium (50 mM) alone using a fresh glassy carbon electrode. scan rate:0.1 V/sec.

The Product Selectivity of ORR

The RRDE was employed to analyze the production distribution of ORR. The ORR product can appear either as water, resulting from the transfer of four protons and electrons, or hydrogen peroxide, generated through the transfer of two protons and electrons. The product selectivity was probed at two potentials, one is at −1.2 V (vs. $Fc^{+/0}$) which is more positive than the reduction potential of superoxide formation (−1.28 V vs $Fc^{+/0}$). The other potential is at 0.23 V of the overpotential of the standard equilibrium potential of the HER reaction of each Brønsted acid used.

The standard equilibrium potential of the HER reaction of each Brønsted acid can be calculated with the following equation:

$$E_{BH^+} = E^o_{BH^+} - (\frac{2.303RT}{F})pK_a$$

where $E^o_{BH^+}$ is –0.028 V (vs. $Fc^{+/0}$) in ACN.[2]

Table S2. The correlation between product selectivity of ORR reaction and the pK_a of acid.

	pK_a	E_{BH^+} (V vs. $Fc^{+/0}$)	The potential with η = 0.23 V
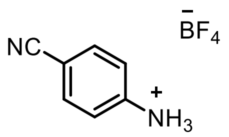	7.0	−0.44	−0.67
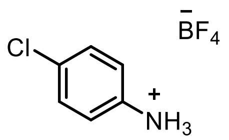	9.7	−0.60	−0.83
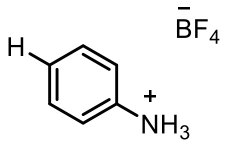	10.6	−0.65	−0.88
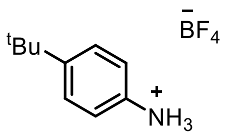	11.1	−0.68	−0.91
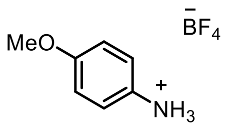	11.9	−0.73	−0.96

Control experiment of electropolymerization of aniline under oxidizing potential

Aniline and its derivatives are known to undergo electropolymerization under oxidizing potentials,[5] such as 0.45 V applied by the ring electrode to detect H_2O_2. To determine whether this process interferes with the measurement of product distribution between H_2O and H_2O_2 at the ring electrode, we performed control experiments using *p*-cyanoanilinium and *p*-methoxyanilinium. As shown in Figure S11, the currents generated by the electropolymerization of these derivatives (blue line) were significantly lower compared to the current observed during the ORR catalyzed by **1** (red line). This confirms that the electropolymerization of anilinium derivatives does not impact the product distribution analysis.

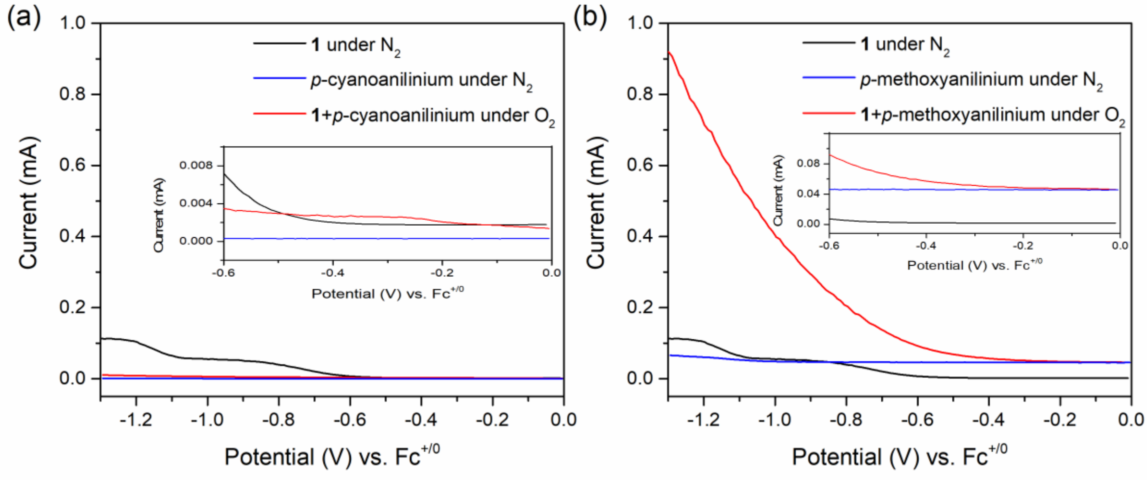

Figure S11, the ring electrode current poised at 0.45 V from different voltammograms scanning between 0 V and -1.3 V in acetonitrile. The black trace: **1** (0.5 mM) in the absence of acid under 100% N_2. The blue trace: 50 mM of *p*-cyanoanilinium (a) and *p*-methoxyanilinium (b) under 100% N_2. The red trace: **1** (0.5 mM) in the presence of respective 50 mM acids under 21% O_2 and 79% N_2. Scan rate: 0.01 V/sec. The inset figure enlarges the area before the ORR takes place.

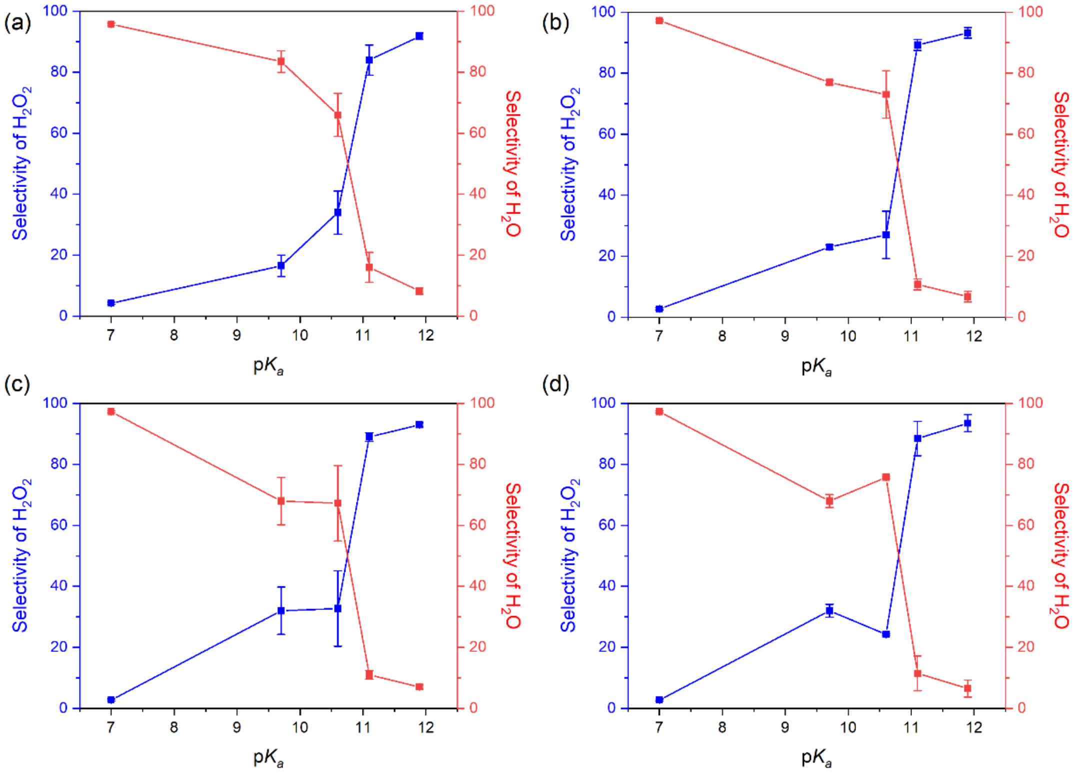

Figure S12. The distribution of product selectivity of ORR electrolyzed at −1.2 V in the presence of **1** (0.5 mM) under different ratios of O_2 and pK_a of acids (50 mM) (see Table S2). The gas composition: (a) 7% O_2 and 93% N_2, (b) 21% O_2 and 79% N_2, (c) 50% O_2 and 50% N_2 and (d) 100% O_2. Rotation rate: 1500 rpm. The corresponding RRDE voltammograms are Figure S27–S31 respectively.

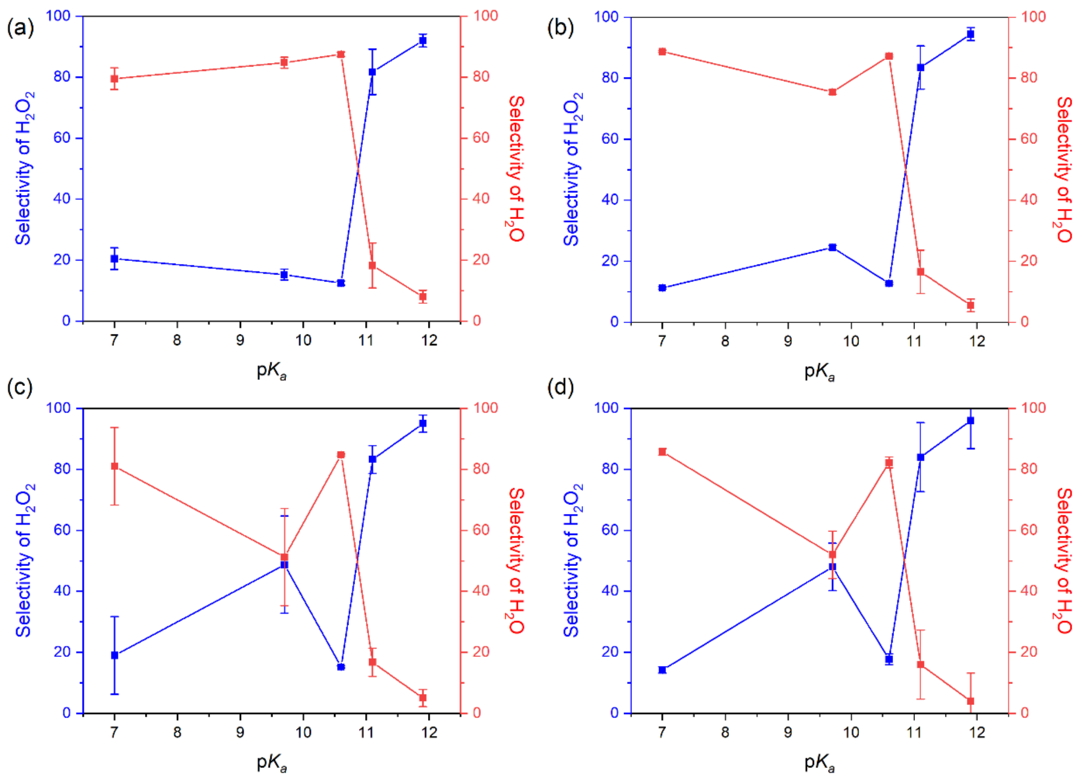

Figure S13. The distribution of product selectivity of ORR electrolyzed at the potential with 0.23 V of overpotential against the equilibrium potential of HER in the presence of **1** (0.5 mM) under different ratios of O_2 and pK_a of acids (50 mM). (see Table S2). The gas composition: (a) 7% O_2 and 93% N_2, (b) 21% O_2 and 79% N_2, (c) 50% O_2 and 50% N_2 and (d) 100% O_2. The concentration of acid in the solution: 50 mM and the rotation rate: 1500 rpm. The corresponding RRDE voltammograms are Figure S27–S31 respectively.

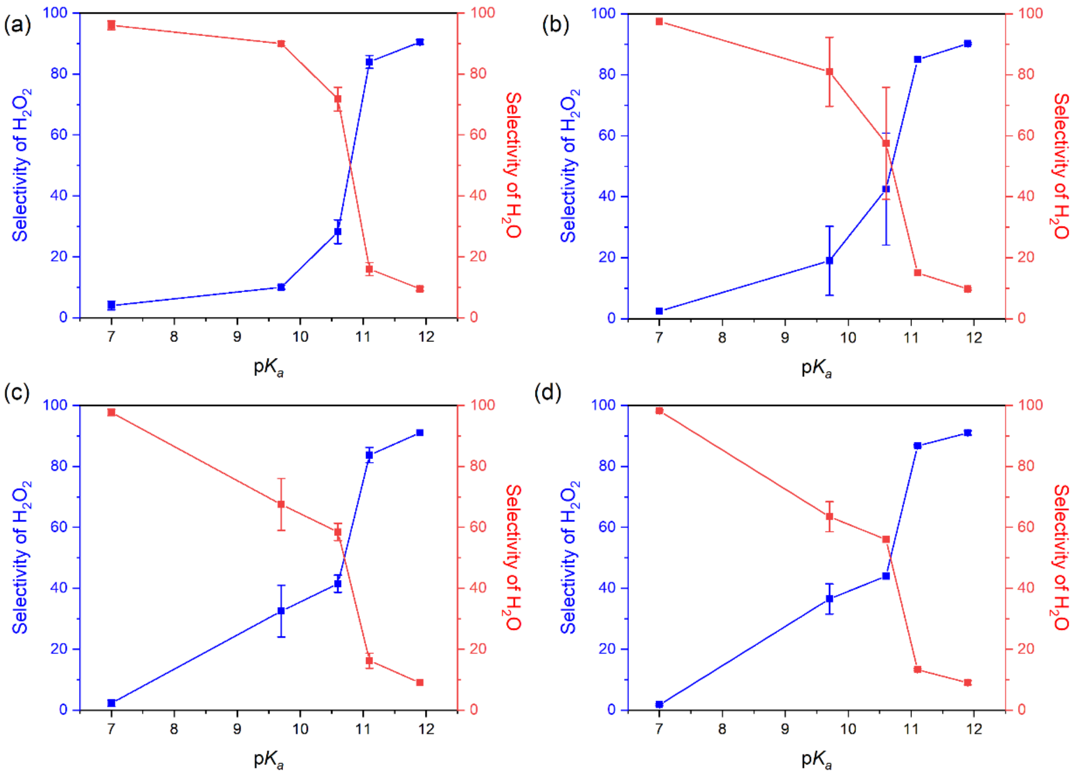

Figure S14. The distribution of product selectivity of ORR electrolyzed at −1.2 V in the presence of **2** (0.5 mM) under different ratios of O_2 and pK_a of acids (50 mM). (see Table S2). The gas composition: (a) 7% O_2 and 93% N_2, (b) 21% O_2 and 79% N_2, (c) 50% O_2 and 50% N_2 and (d) 100% O_2. Rotation rate: 1500 rpm. The corresponding RRDE voltammograms are Figure S32–S36 respectively.

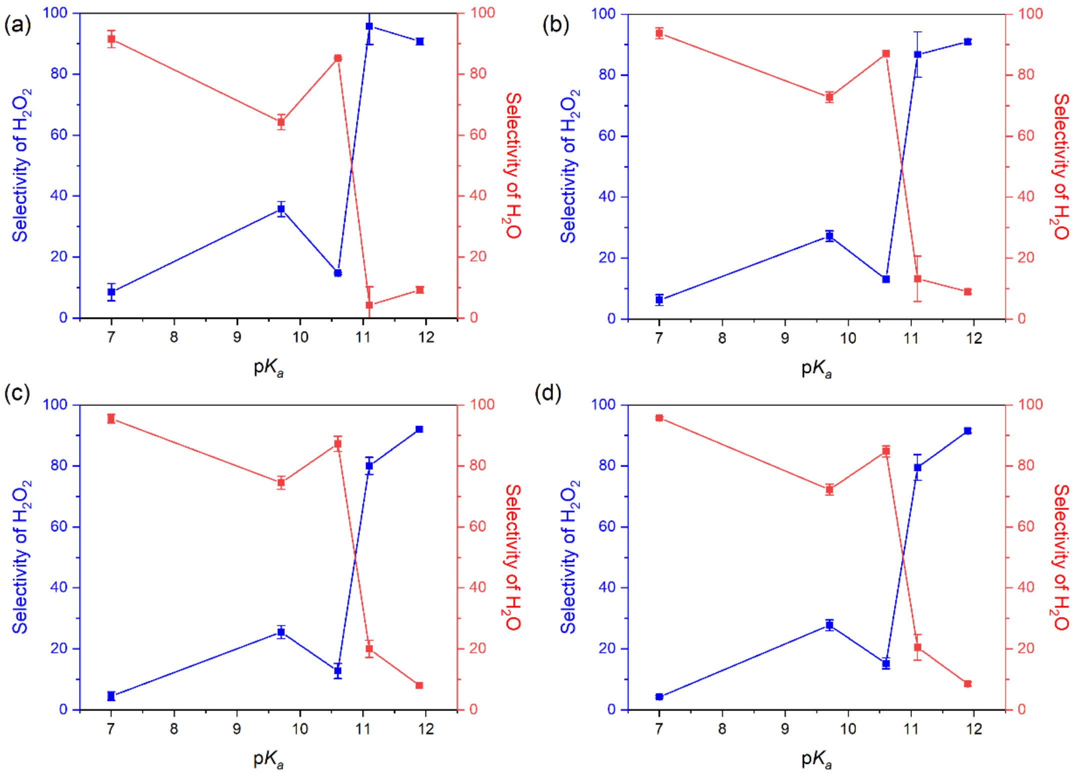

Figure S15. The distribution of product selectivity of ORR electrolyzed at the potential with 0.23 V of overpotential against the equilibrium potential of HER in the presence of **2** (0.5 mM) under different ratios of O_2 and pK_a of acids (50 mM). (see Table S2). The gas composition: (a) 7% O_2 and 93% N_2, (b) 21% O_2 and 79% N_2, (c) 50% O_2 and 50% N_2 and (d) 100% O_2. Rotation rate: 1500 rpm. The corresponding RRDE voltammograms are Figure S32–S36 respectively.

Discussion of the pK_a-potential relationship

The ORR current collected at a disk electrode in the presence of various acids was analyzed and plotted, as shown in Figure S16, to investigate the relationship between acid pK_a and electrode potential. Theoretically, if the ORR were purely thermodynamically controlled, the onset potential of the reaction would be expected to shift by approximately 60 mV per unit increase in the acid's pK_a, reflecting the well-established pK_a-potential relationship. However, no such trend was observed in our system. This deviation is likely due to the simultaneous occurrence of two competing ORR reactions across all the acids (2 < n (average electron transfer number) < 4) examined in this study. Although the product selectivity is strongly influenced by the pK_a of the acids employed, the coexistence of these competing reaction pathways disrupts the expected pK_a-potential correlation.

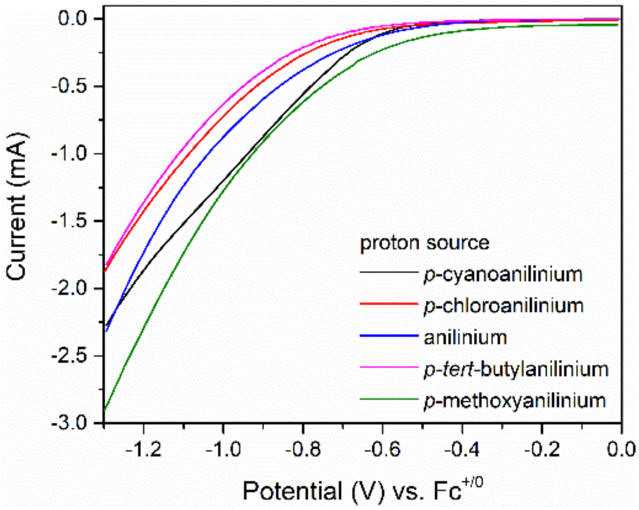

Figure S16. The current collected from the disk electrode of the RRDE system under 21% of O_2 and 79% of N_2 in the presence of different proton sources. Scan rate: 0.01 V/sec. The corresponding RRDE voltammograms are Figure S27–S31 respectively.

The Foot of the Wave Analysis (FOWA)

The foot of the wave analysis was used to identify the rate-determining step in the reaction mechanism based on the shape of the plot derived from the following equation:

$$i = \frac{nFAC^*\sqrt{Dk_{FOWA}}}{1 + exp[\frac{nF}{RT}(E - E_1)]} \qquad \text{Eq S3}$$

where *i*: catalytic current, *n*: number of electrons transferred, *F*: Faraday's constant, *A*: geometric electrode surface area, C^*: concentration of catalysts, *D*: diffusion coefficient. *R*: gas constant. *T*: temperature, *E*: potential applied. E_1: half-peak potential ($E_{1/2}$) of the Co(III)/Co(II) couple.

According to reference 6, 7 and 8, the shape of the FOWA plot obtained from an electrocatalytic multi-electron reaction, such as the ORR here, can provide useful information to diagnose if the first chemical step is a rate-limiting step or not. If the resulting FOWA plot is a curve shape, this suggests the first chemical step is not the rate-limiting step. More detailed discussions can be found in references.[7, 8]

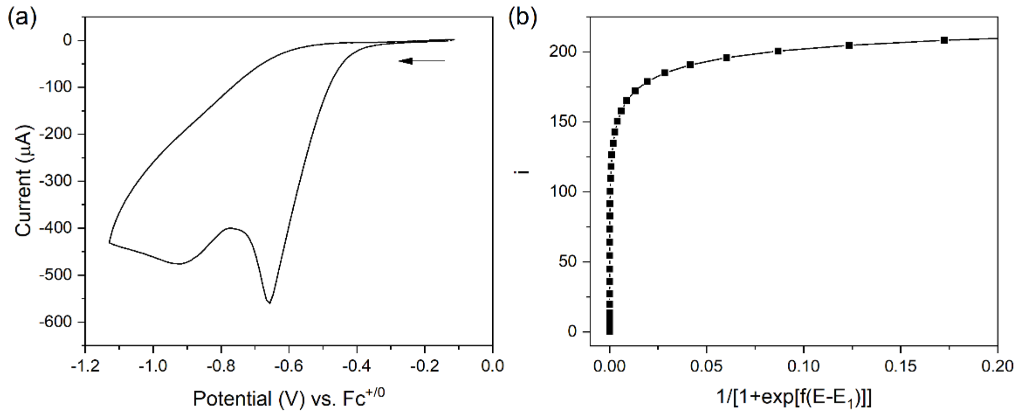

Figure S17. (a) The cyclic voltammogram of **1** (0.5 mM) with *p*-cyanoanilinium (5 mM) in acetonitrile under 21% oxygen and 79% nitrogen and scan rate: 3 V/s. (b) The FOWA plot derived from (a) according to eq S3.

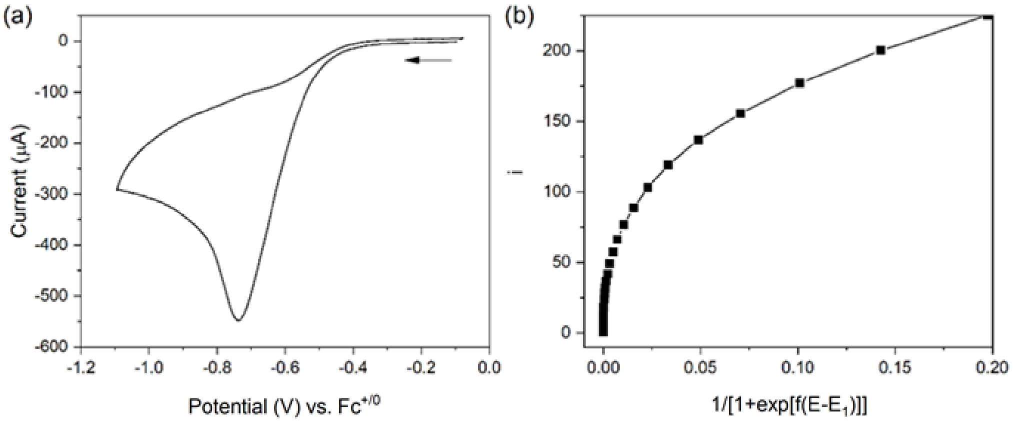

Figure S18. (a) The cyclic voltammogram of **1** (0.5 mM) with *p*-methoxyanilinium (5 mM) in acetonitrile under 21% oxygen and 79% nitrogen and scan rate: 3 V/s. (b) The FOWA plot derived from (a) according to eq S3.

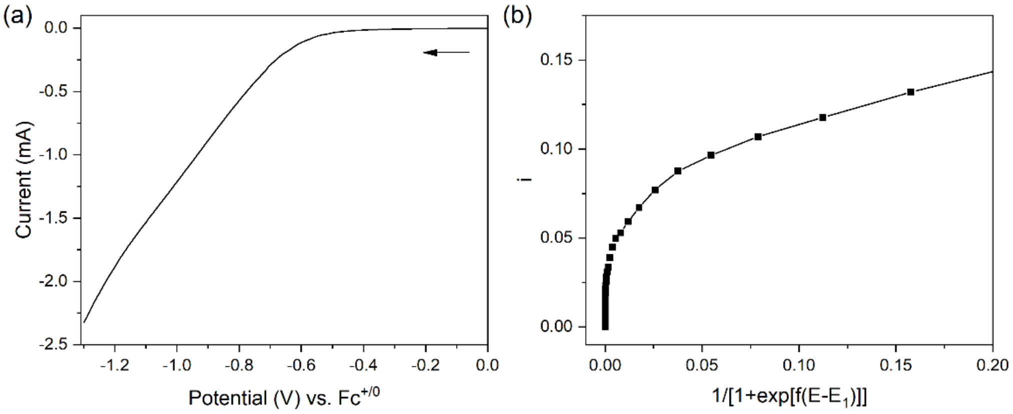

Figure S19. (a) The linear sweep voltammogram of **1** (0.5 mM) with *p*-cyanoanilinium (50 mM) in acetonitrile collected from the RRDE system under 21% oxygen and 79% nitrogen and scan rate: 0.01 V/s. (b) The FOWA plot derived from (a) according to eq S3.

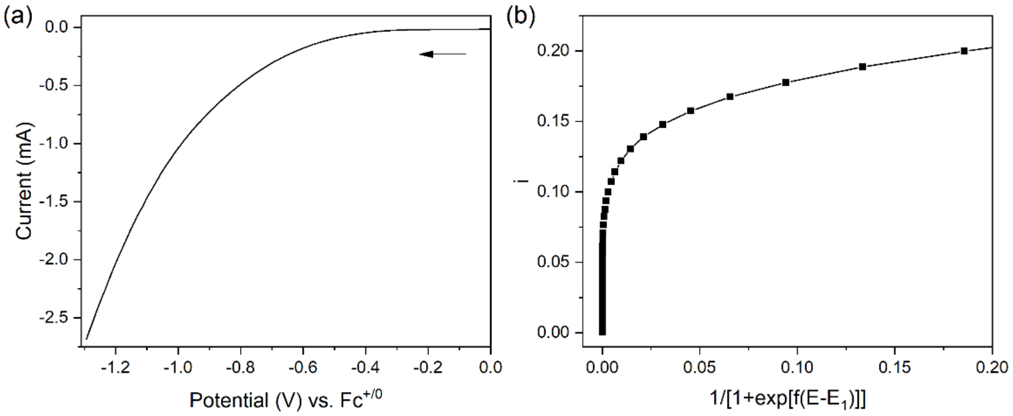

Figure S20. (a) The linear sweep voltammogram of **1** (0.5 mM) with *p*-methoxyanilinium (50 mM) in acetonitrile collected from the RRDE system under 21% oxygen and 79% nitrogen and scan rate: 0.01 V/s. (b) The FOWA plot derived from (a) according to eq S3.

The Determination of ORR Reaction Rate Law

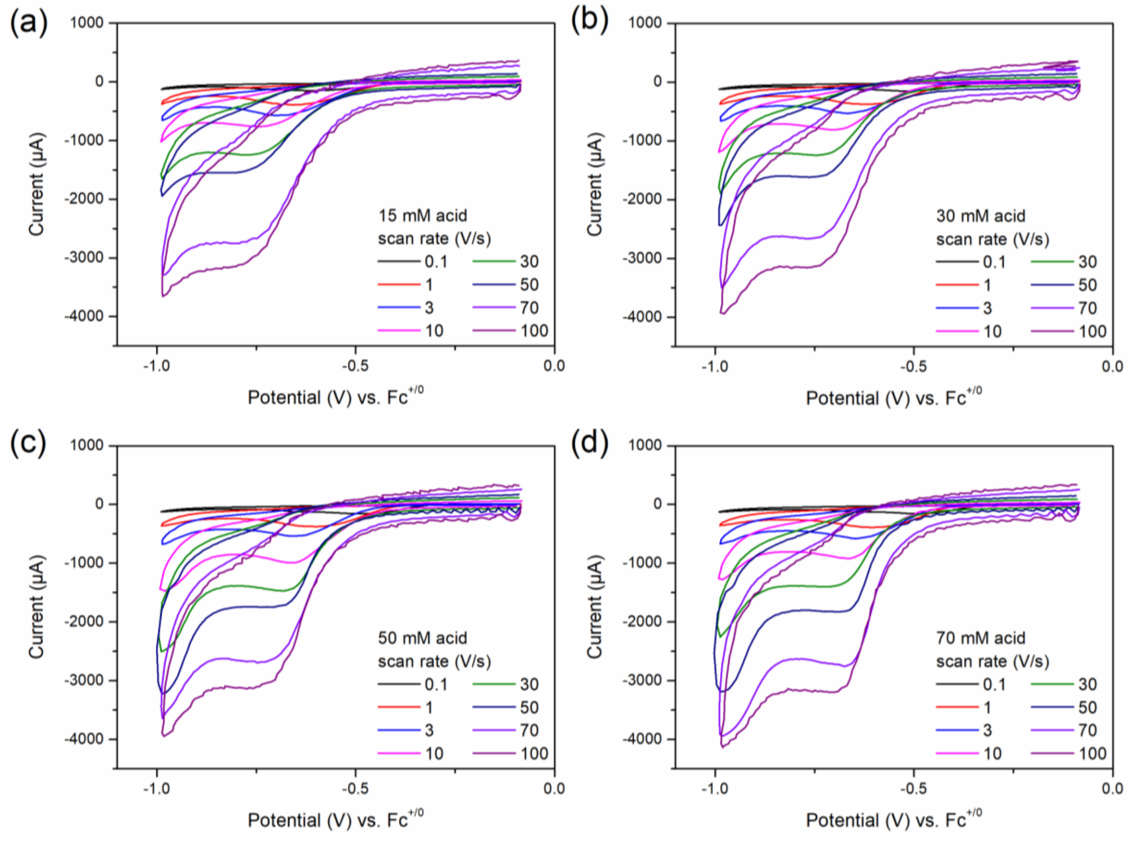

Figure S21. Electrocatalytic voltammograms of complex **1** (0.5 mM) recorded in the presence of varying concentrations of *p*-cyanoanilinium tetrafluoroborate from 15 mM to 70 mM under an atmospheric composition of 21% O_2 and 79% N_2 with different scan rates.

The determination of ORR reaction rate law in the presence of *p*-methoxyanilinium.

To comprehend the rate law of the ORR reaction in the presence of *p*-methoxyanilinium, cyclic voltammograms were collected with varying concentrations of acids and oxygen to probe the correlation between the plateau current and concentration.

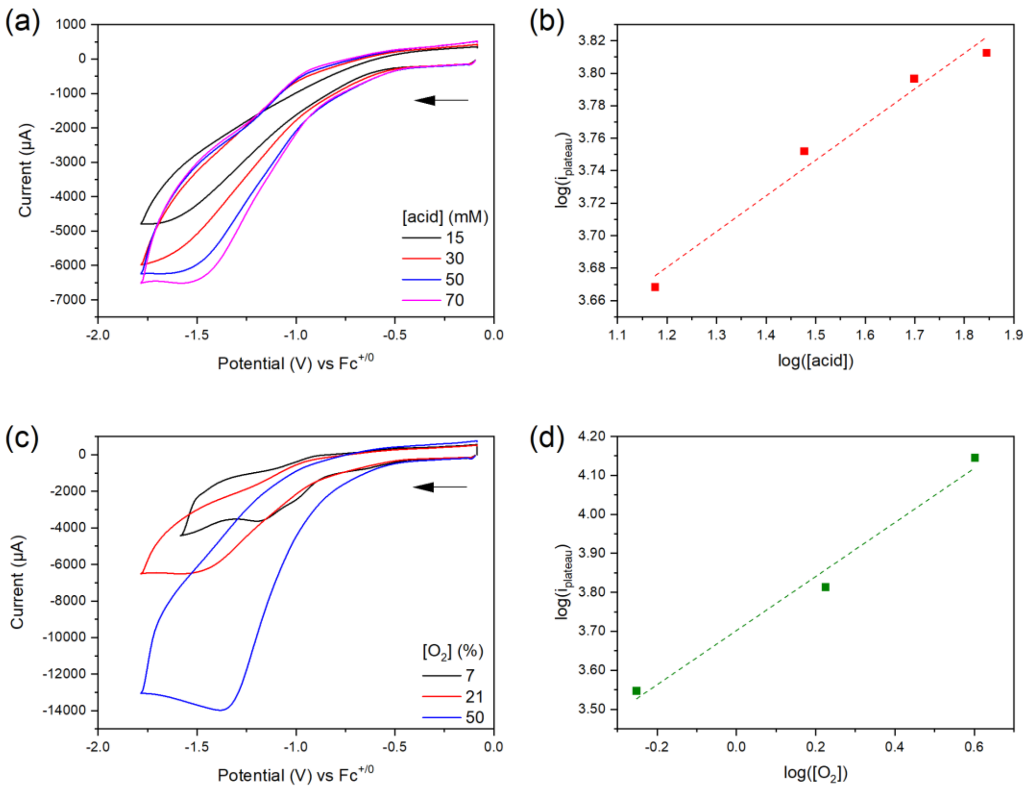

Figure S22. (Left) Cyclic voltammograms of electrocatalysis by **1** in the presence of the *p*-methoxyanilinium, and (Right) the logarithm of the plateau current with the logarithm of concentration. (a) different concentrations of acid and [**1**] = 0.5 mM, υ = 100 V/s and under 21% O_2 and 79% N_2. (c) Different ratios of dioxygen from 7% O_2 to 50% O_2 balancing with N_2. [**1**] = 0.5 mM, υ=100 V/s, [acid] = 70 mM.

UV-vis Spectroscopy

The reaction between product and 1 monitored by UV-vis spectroscopy.

The UV-Vis spectra were acquired using an Agilent Cary 5000 spectrophotometer, conducted in dry acetonitrile. To assess the stability of **1** with the product of ORR, the various concentrations of water and hydrogen peroxide were mixed with **1**. The concentration of **1** was maintained at 0.01 M, with varying concentrations of water or hydrogen peroxide added: 0 (black line), 0.005, 0.01, 0.05, 0.1, 0.25, 0.5, and 1.0 M(red line).

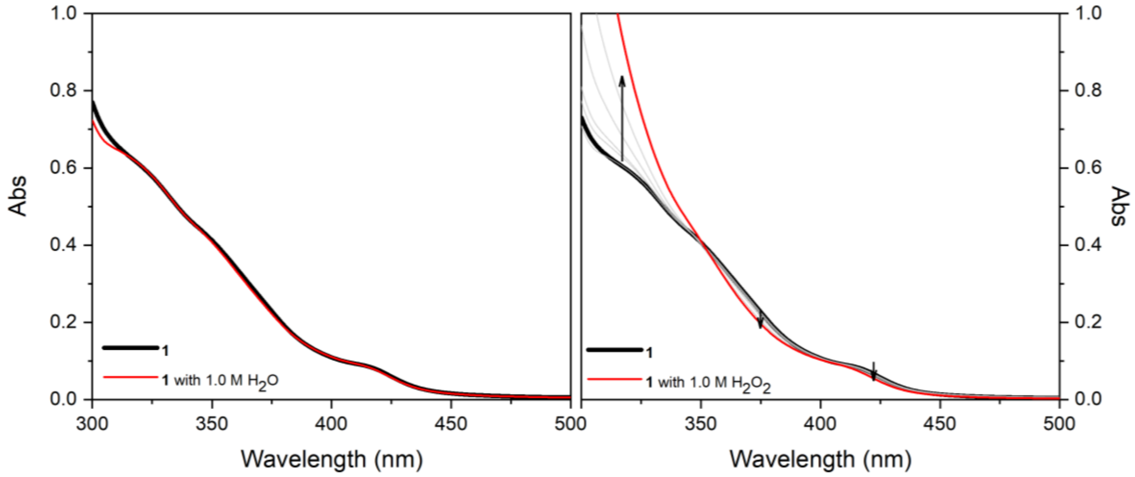

Figure S23. The UV-vis spectra of **1** mixed with different concentrations of (left) water and (right) hydrogen peroxide.

The Role of Axis Ligand

The synthesis of the cobalt diimine-dioxime complex with two labile acetonitrile molecules (**2**) was successfully achieved and identified by NMR (Figure S41, 42), ESI-MS (Figure S43–45). The crystal structure was determined using X-ray crystallography.

Cyclic voltammogram of **2** showed a significantly more positive redox couple peak for the Co(III)/(II) species compared to **1**. Both complexes exhibited nearly identical reduction potentials for the Co(II)/(I) species, indicating the same coordination environment for the complex upon formation of the Co(II) species.

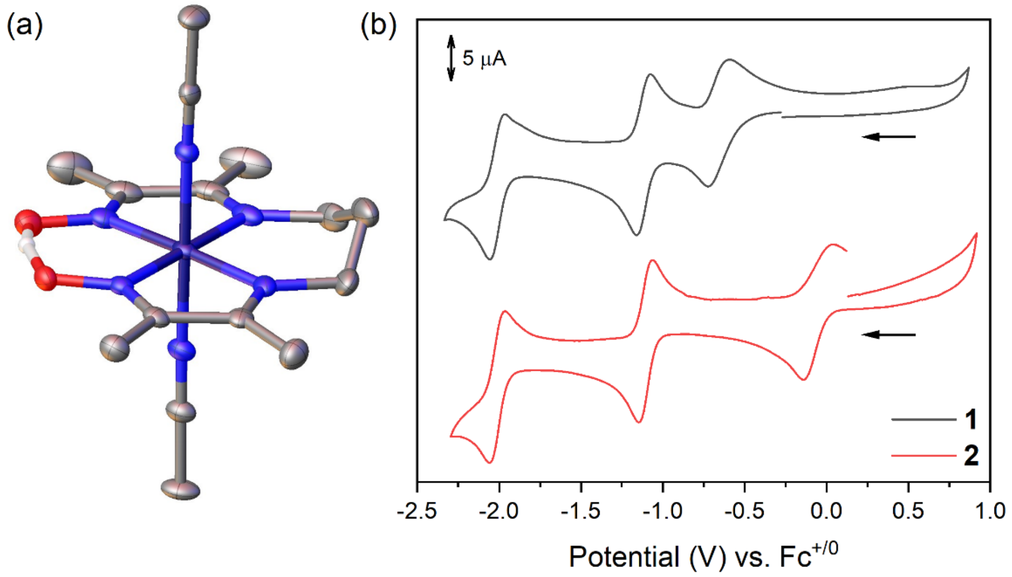

Figure S24. (a) The X-ray crystal structure of **2**. For clarity, hydrogen, perchlorate and acetonitrile (solvent) were omitted. The detail and preparation step of the crystal can be obtained in Figure S46 (b) The cyclic voltammograms of **1** and **2** (0.5 mM) in acetonitrile in the absence of acid under nitrogen. Scan rate: 0.1 V/s.

The elemental analysis result of **2** was shown in the following table (Table S3), which does not fit the predicted chemical formula derived from X-ray crystal structure. The discrepancy between the predicted chemical formula and the experimental formula is probably due to sample preparations. The crystal used in the X-ray crystallography contained ACN as the solvent, favoring the formation of a complex with two ACN axial

ligands. However, before elemental analysis, the samples were under vacuum to remove solvent, leaving the sample mostly solvent-free. During transport, one of the coordinated ACN molecules might have been replaced by an H_2O molecule, leading to a different axial coordination environment. Furthermore, the ESI-MS data (Figure S43) also showed the mass of a complex corresponded to Co-diimine-dioxime-(ACN)(H_2O)-(ClO_4)$_2$ at 178.56 m/z, suggesting that one of the ACN molecules can easily be replaced by H_2O.

	N%	C%	H%
Trail 1	12.28	27.76	4.37
Trail 2	12.86	27.90	4.49
Co-diimine-dioxime -(ACN)$_2$-(ClO_4)$_2$ (**2**)			
Calcd	14.51	31.10	4.35
Co-diimine-dioxime -(ACN)(H_2O) -(ClO_4)$_2$			
Calcd	12.59	28.07	4.35

Table S3. The elemental results of complex **2**.

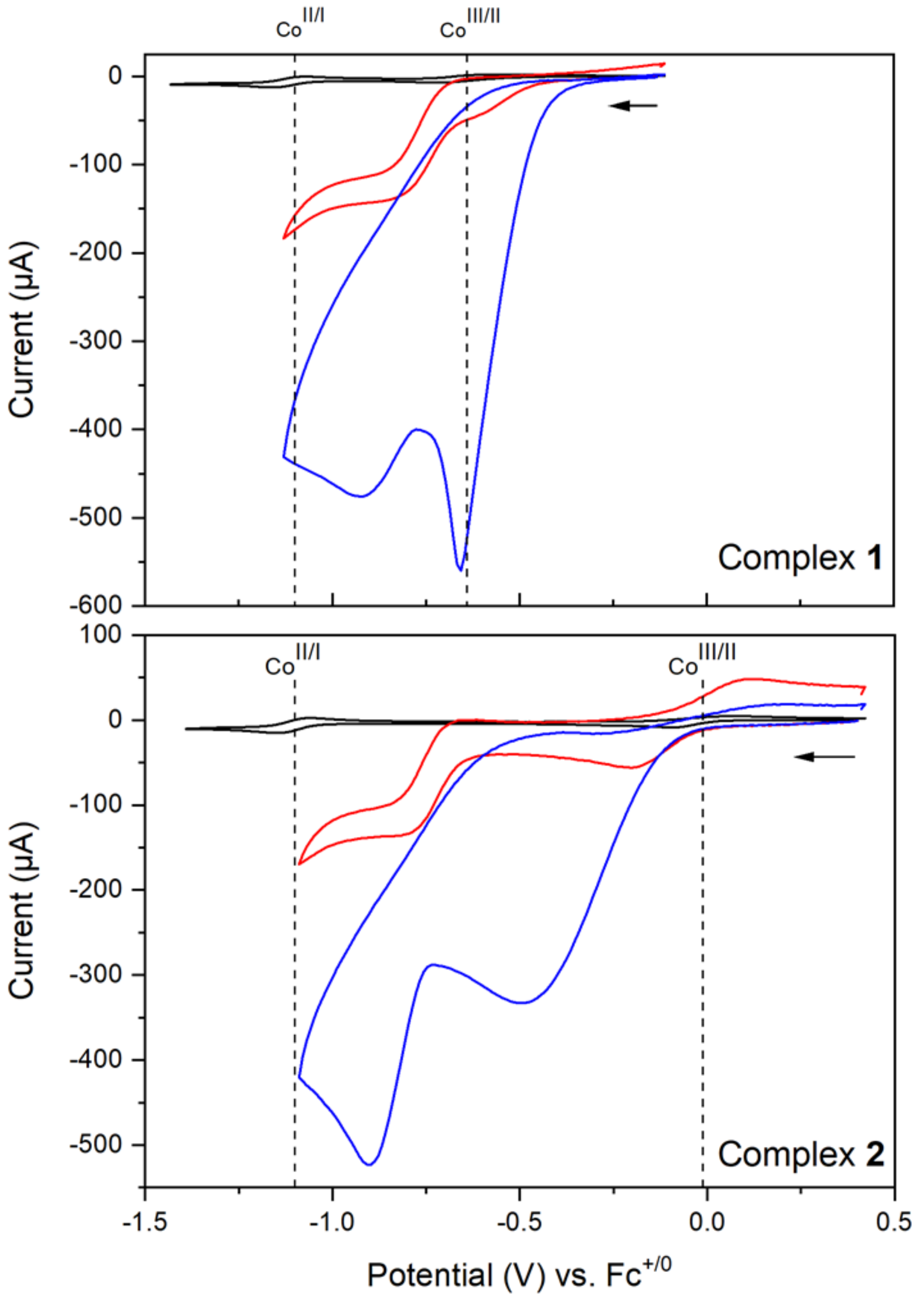

Figure S25. Cyclic voltammograms of (a) **1** (0.5 mM) and (b) **2** (0.5 mM) in ACN under different conditions. The black trace: the voltammogram obtained under 100% N_2 with the scan rate of 0.1 V/s. The red and blue voltammograms were collected in the presence of 10 equivalents of *p*-cyanoanilinium with 3 V/s under two different respective gas compositions respectively. The red trace: 100% nitrogen and the blue trace: 79% N_2 and 21% O_2.

Control experiments

To explore the possible nature of the cross point found in some voltammograms in Figure 4, further control experiments were performed. The cross point in cyclic voltammograms is known to occur when the mechanism is involved in the ECE step.[9] As seen in Figure S26, upon the addition of a proton source (blue trace), the onset potential for $O_2/O_2^{\bullet-}$ shifts anodically, likely due to a reaction between $O_2^{\bullet-}$ and protons, producing reactive species such as $HO_2^{\bullet}$, HO_2^-, or H_2O_2.[10] The region observed for these regions overlapped the region where the cross point were observed. Therefore, during the experiment shown in Figure S26, these O_2-derived intermediates were also generated at more negative potentials and underwent an ECE reaction with the catalysts

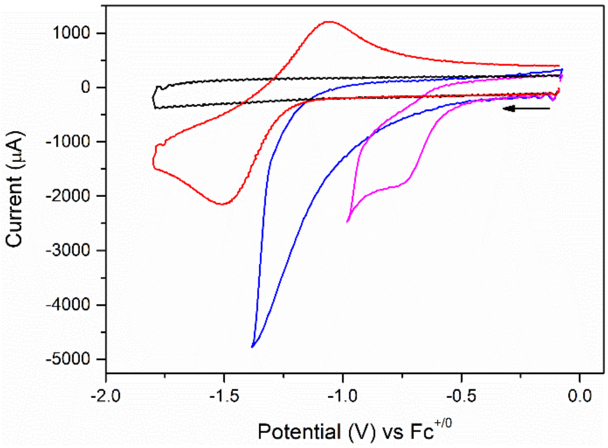

Figure S26. Cyclic voltammograms of ACN under various conditions at a scan rate of 100 V/s. The black trace: only ACN solution under 100% nitrogen. The red trace: only ACN solution under 79% nitrogen and 21% oxygen. The blue trace: the ACN solution with 70 mM of *p*-cyanoanilnium under 79% nitrogen and 21% oxygen. The magenta trace: **1** (0.5 mM) with 70 mM of *p*-cyanoanilnium in ACN under 79% nitrogen and 21% oxygen.

RRDE Data

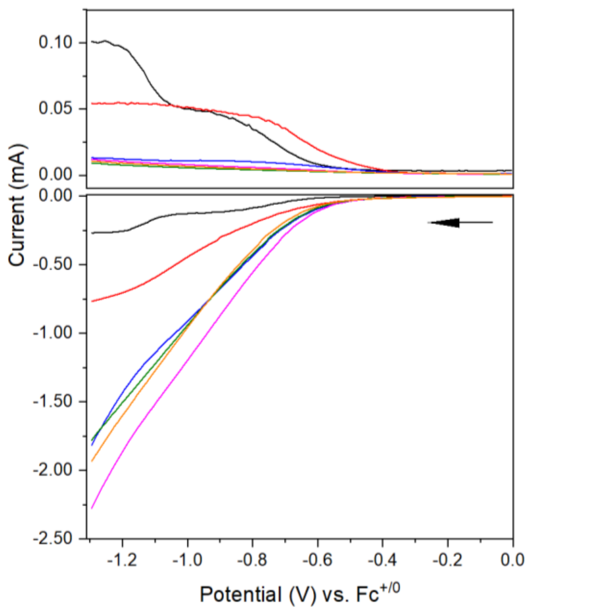

Figure S27. The RRDE of 0.5 mM **1** with 50 mM *p*-cyanoanilinium under different ratios of gas in ACN containing 0.1 M $TBAPF_6$ at 1500 rpm. Scan rate: 0.01 V/s. (black) **1** without acid under nitrogen, **1** with acid under nitrogen (red). $[O_2]$ = 7% (blue), 21% (magenta), 50% (olive) 100% (orange) with the remainder filled with nitrogen.

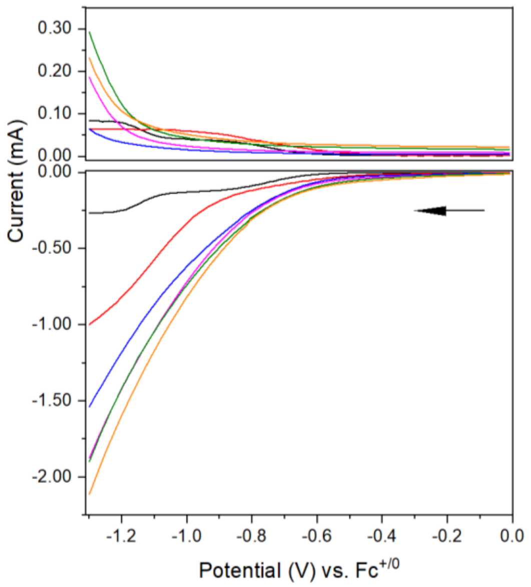

Figure S28. The RRDE of 0.5 mM **1** with 50 mM *p*-chloroanilinium under different ratios of gas in ACN containing 0.1 M $TBAPF_6$ at 1500 rpm. Scan rate: 0.01 V/s. **1** without acid under nitrogen (black), **1** with acid under nitrogen (red). $[O_2]$ = 7% (blue), 21% (magenta), 50% (olive) 100% (orange) with the remainder filled with nitrogen.

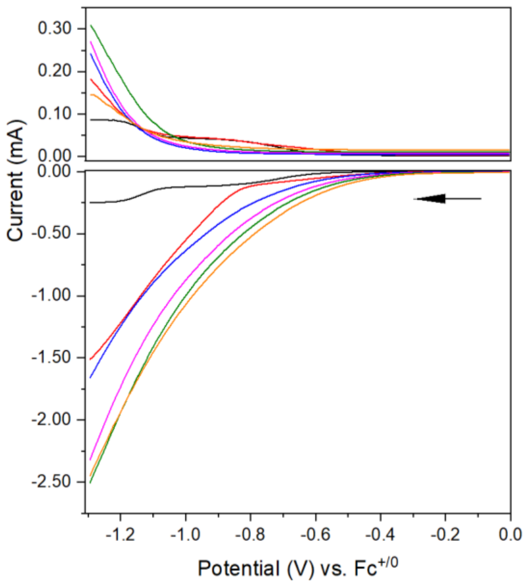

Figure S29. The RRDE of 0.5 mM **1** with 50 mM anilinium under different ratios of gas in ACN containing 0.1 M $TBAPF_6$ at 1500 rpm. Scan rate: 0.01 V/s. **1** without acid under nitrogen (black), **1** with acid under nitrogen (red). $[O_2]$ = 7% (blue), 21% (magenta), 50% (olive) 100% (orange) with the remainder filled with nitrogen.

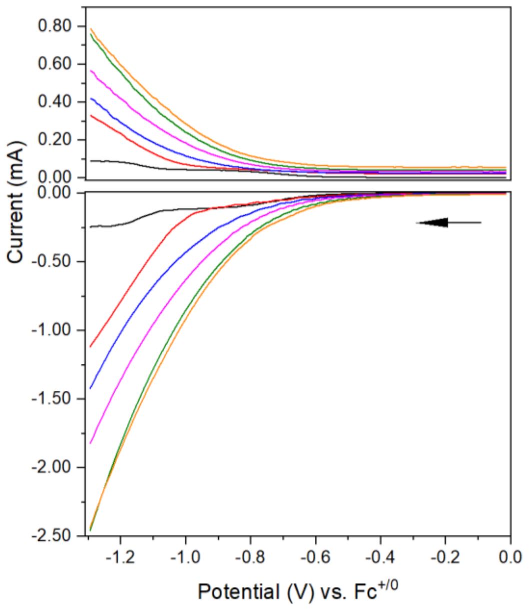

Figure S30. The RRDE of 0.5 mM **1** with 50 mM *p*-*tert*-butylanilinium under different ratios of gas in ACN containing 0.1 M $TBAPF_6$ at 1500 rpm. Scan rate: 0.01 V/s. **1** without acid under nitrogen (black), **1** with acid under nitrogen (red). $[O_2]$ = 7% (blue), 21% (magenta), 50% (olive) 100% (orange) with the remainder filled with nitrogen.

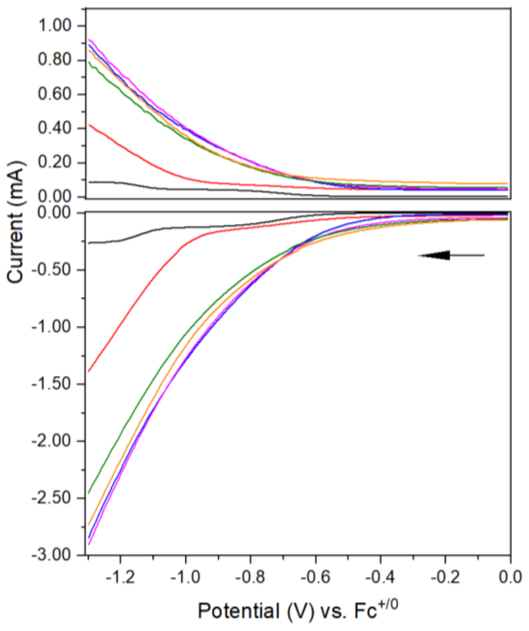

Figure S31. The RRDE of 0.5 mM **1** with 50 mM *p*-methoxyanilinium under different ratios of gas in ACN containing 0.1 M $TBAPF_6$ at 1500 rpm. Scan rate: 0.01 V/s. **1** without acid under nitrogen (black), **1** with acid under nitrogen (red). $[O_2]$ = 7% (blue), 21% (magenta), 50% (olive) 100% (orange) with the remainder filled with nitrogen.

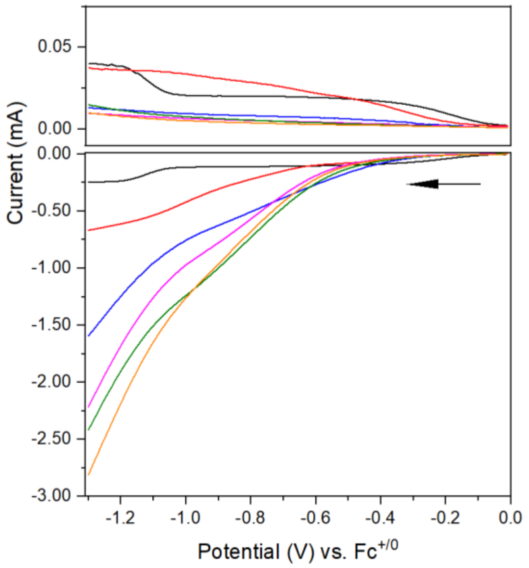

Figure S32. The RRDE of 0.5 mM **2** with 50 mM *p*-cyanoanilinium under different ratios of gas in ACN containing 0.1 M $TBAPF_6$ at 1500 rpm. Scan rate: 0.01 V/s. **2** without acid under nitrogen (black), **2** with acid under nitrogen (red). $[O_2]$ = 7% (blue), 21% (magenta), 50% (olive) 100% (orange) with the remainder filled with nitrogen.

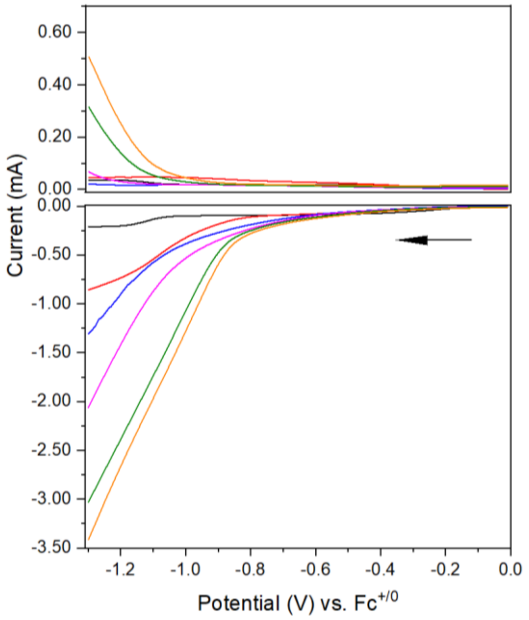

Figure S33. The RRDE of 0.5 mM **2** with 50 mM *p*-chloroanilinium under different ratios of gas in ACN containing 0.1 M $TBAPF_6$ at 1500 rpm. Scan rate: 0.01 V/s. **2** without acid under nitrogen (black), **2** with acid under nitrogen (red). $[O_2]$ = 7% (blue), 21% (magenta), 50% (olive) 100% (orange) with the remainder filled with nitrogen.

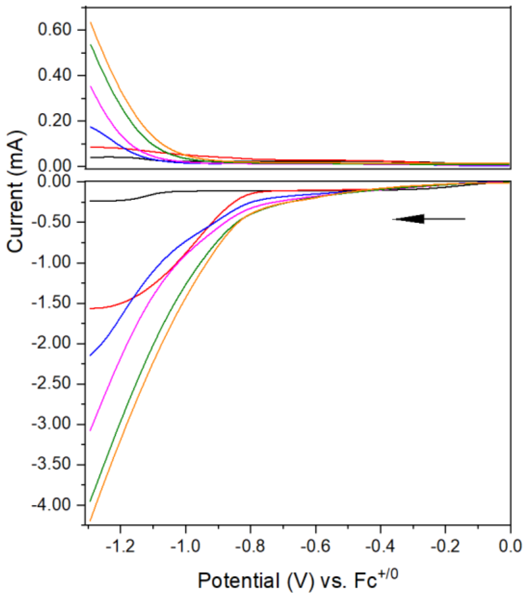

Figure S34. The RRDE of 0.5 mM **2** with 50 mM anilinium under different ratios of gas in ACN containing 0.1 M $TBAPF_6$ at 1500 rpm. Scan rate: 0.01 V/s. **2** without acid under nitrogen (black), **2** with acid under nitrogen (red). $[O_2]$ = 7% (blue), 21% (magenta), 50% (olive) 100% (orange) with the remainder filled with nitrogen.

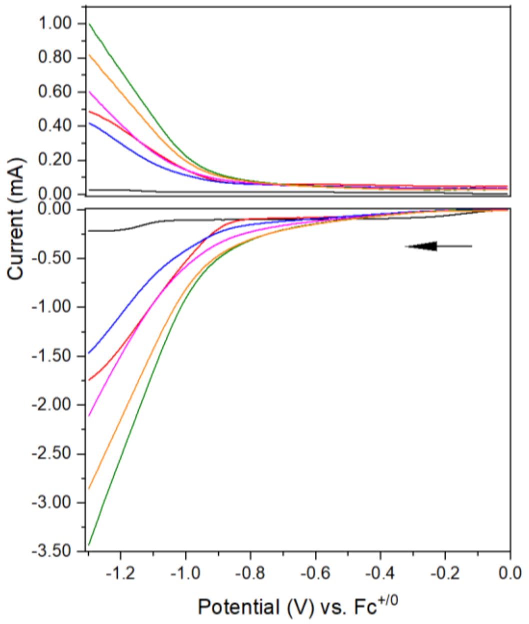

Figure S35. The RRDE of 0.5 mM **2** with 50 mM *p-tert*-butylanilinium under different ratios of gas in ACN containing 0.1 M $TBAPF_6$ at 1500 rpm. Scan rate: 0.01 V/s. **2** without acid under nitrogen (black), **2** with acid under nitrogen (red). $[O_2]$ = 7% (blue), 21% (magenta), 50% (olive) 100% (orange) with the remainder filled with nitrogen.

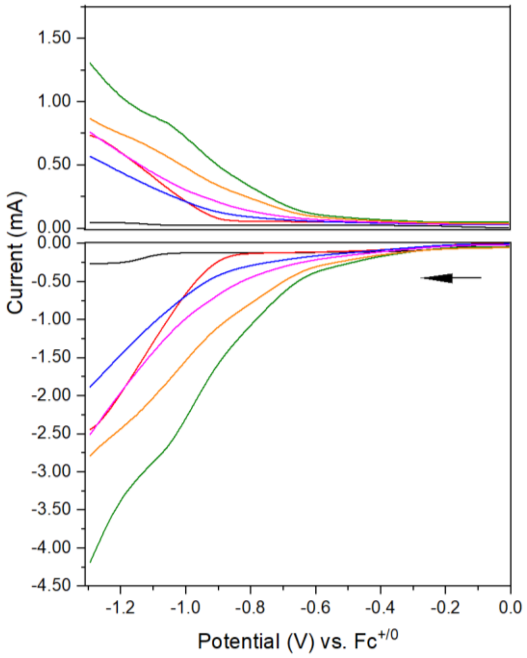

Figure S36. The RRDE of 0.5 mM **2** with 50 mM *p*-methoxyanilinium under different ratios of gas in ACN containing 0.1 M $TBAPF_6$ at 1500 rpm. Scan rate: 0.01 V/s. **2** without acid under nitrogen (black), **2** with acid under nitrogen (red). $[O_2]$ = 7% (blue), 21% (magenta), 50% (olive) 100% (orange) with the remainder filled with nitrogen.

Supplementary Figures

^{1}H-NMR of Diimine-dioxime ligand

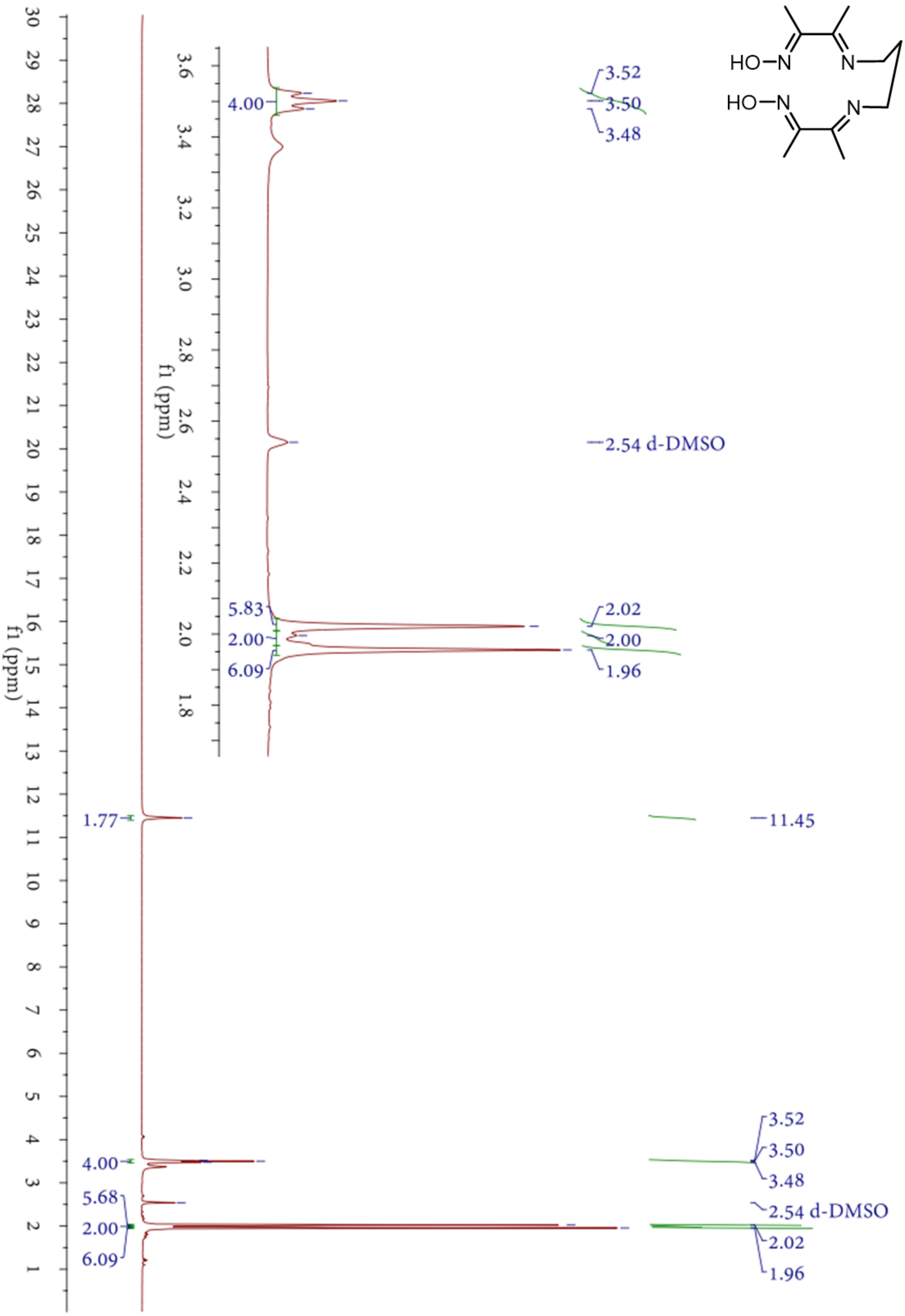

Figure S37. The ^{1}H-NMR of diimine-dioxime ligand.

^{1}H-NMR of complex 1

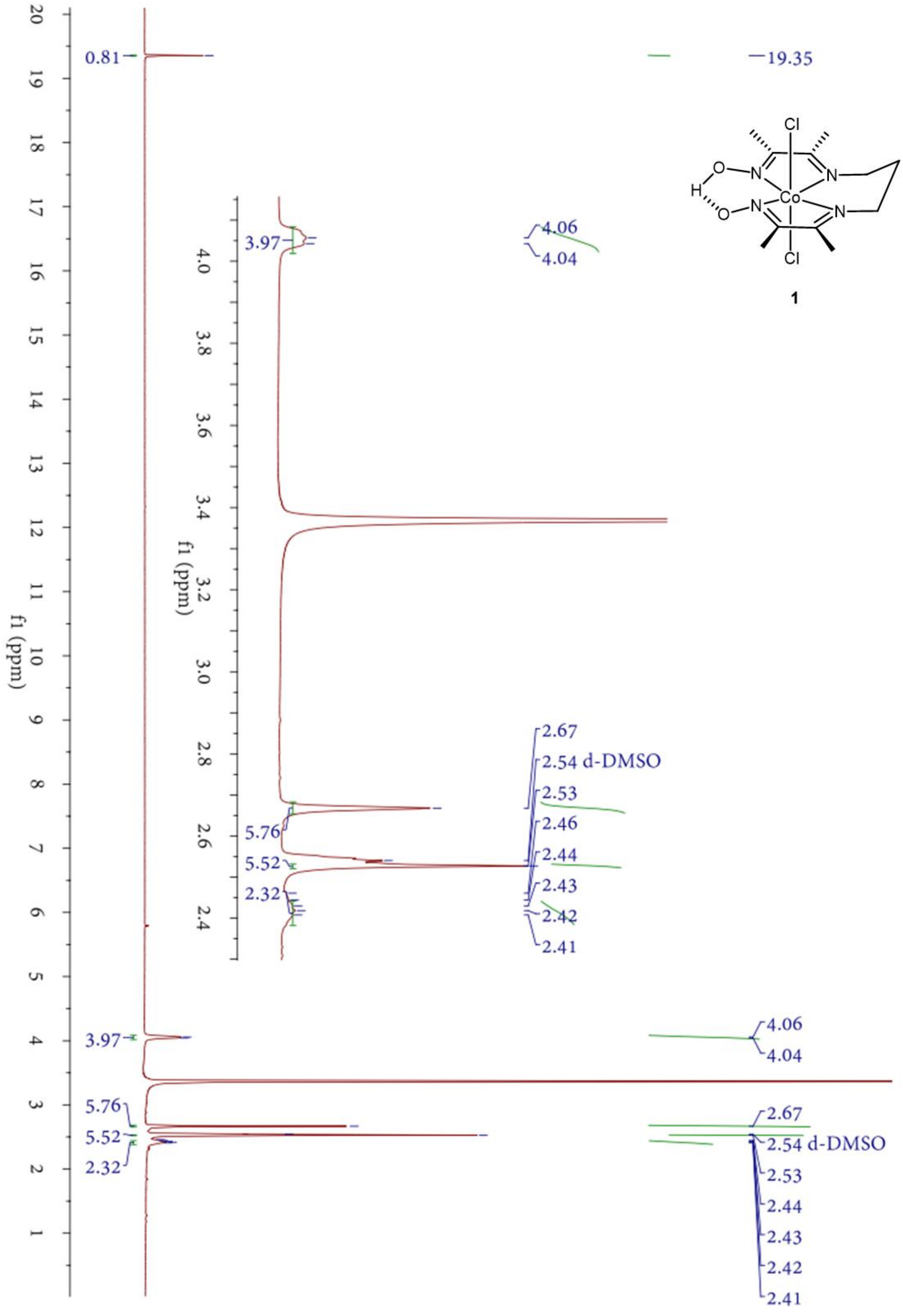

Figure S38. The ^{1}H-NMR of complex **1**.

^{13}C-NMR of complex 1

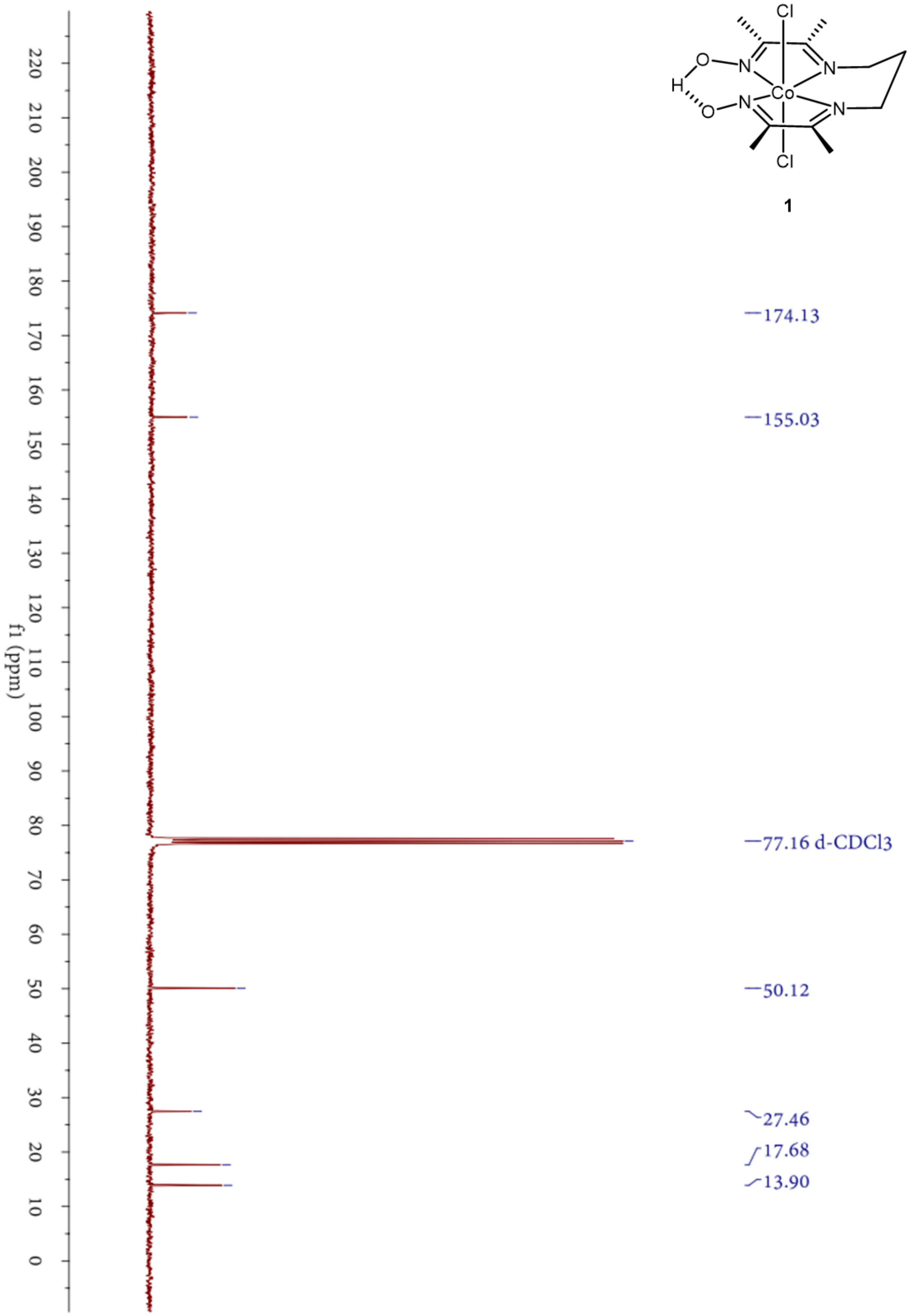

Figure S39. The ^{13}C-NMR of complex **1**.

^{1}H-NMR of $CoDD(H_2O)_2$-$(ClO_4)_2$

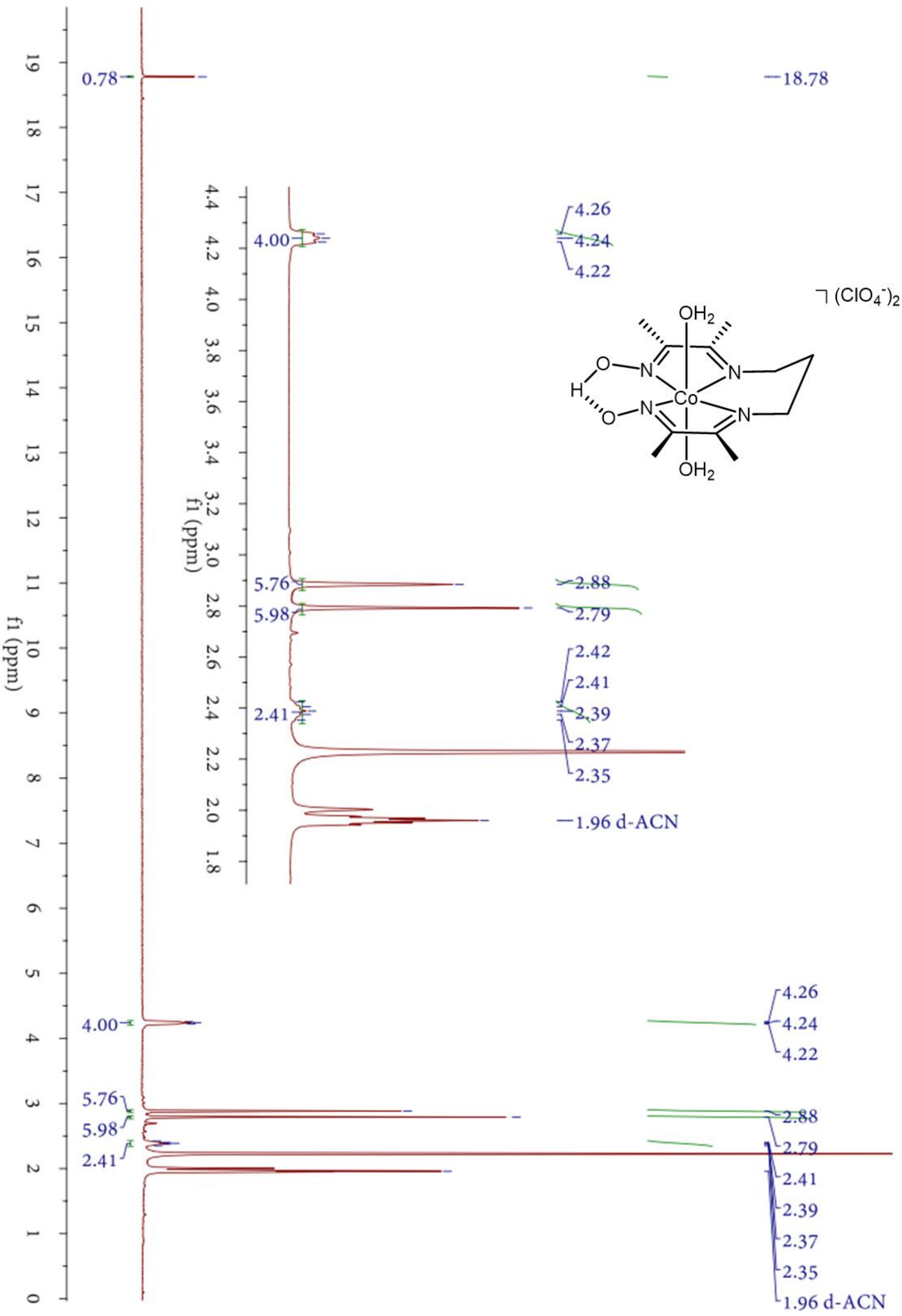

Figure S40. The ^{1}H-NMR of complex **CODD(H_2O)$_2$-(ClO_4)$_2$**.

^{1}H-NMR of complex 2

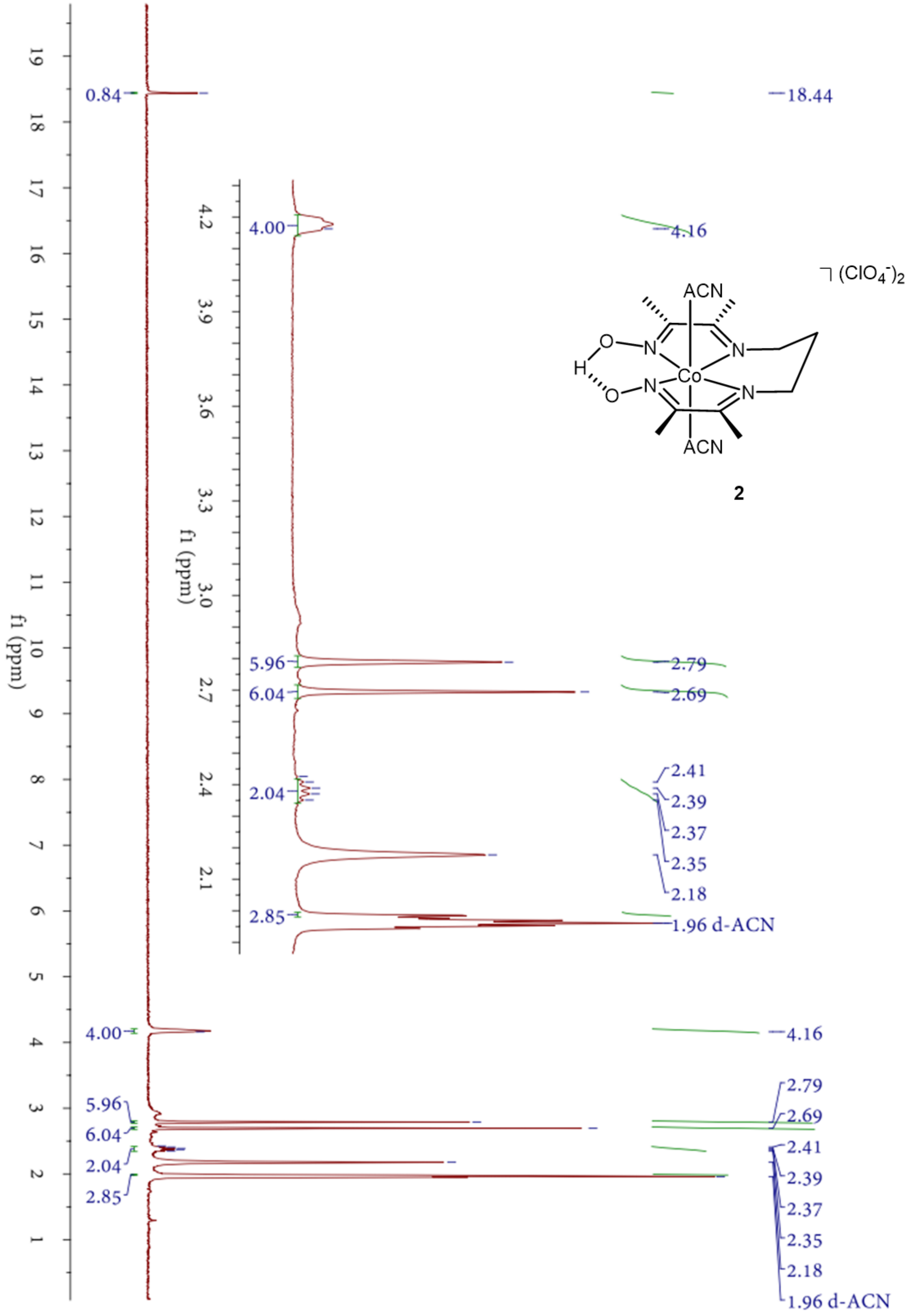

Figure S41. The ^{1}H-NMR of complex **2**.

^{13}C-NMR of complex 2

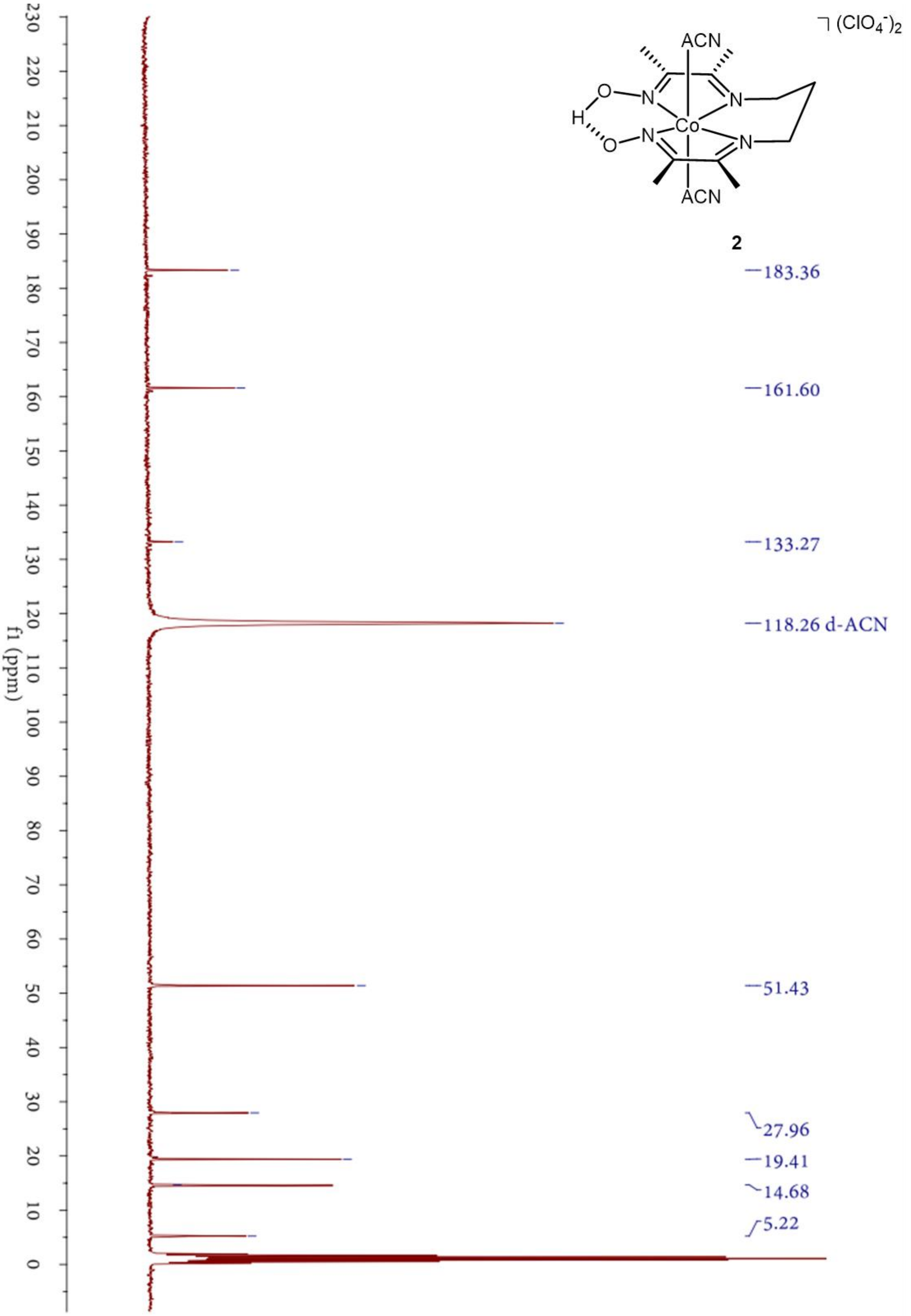

Figure S42. The ^{13}C-NMR of complex **2**.

ESI-MS for Complex 2

Low-resolution ESI-MS of complex 2

m/z calc. $[CoDD\text{-}(ACN)_2]^{2+}$:190.07, found: 190.07

m/z calc. $[CoDD\text{-}(ACN)(H_2O)]^{2+}$:178.56, found: 178.56

m/z calc. $[CoDD\text{-}(ACN)]^{2+}$:169.55, found: 169.55

m/z calc. $[CoDD\text{-}(H_2O)]^{2+}$:158.05, found: 158.05

m/z calc. $[CoDD]^{2+}$:149.04, found: 149.04

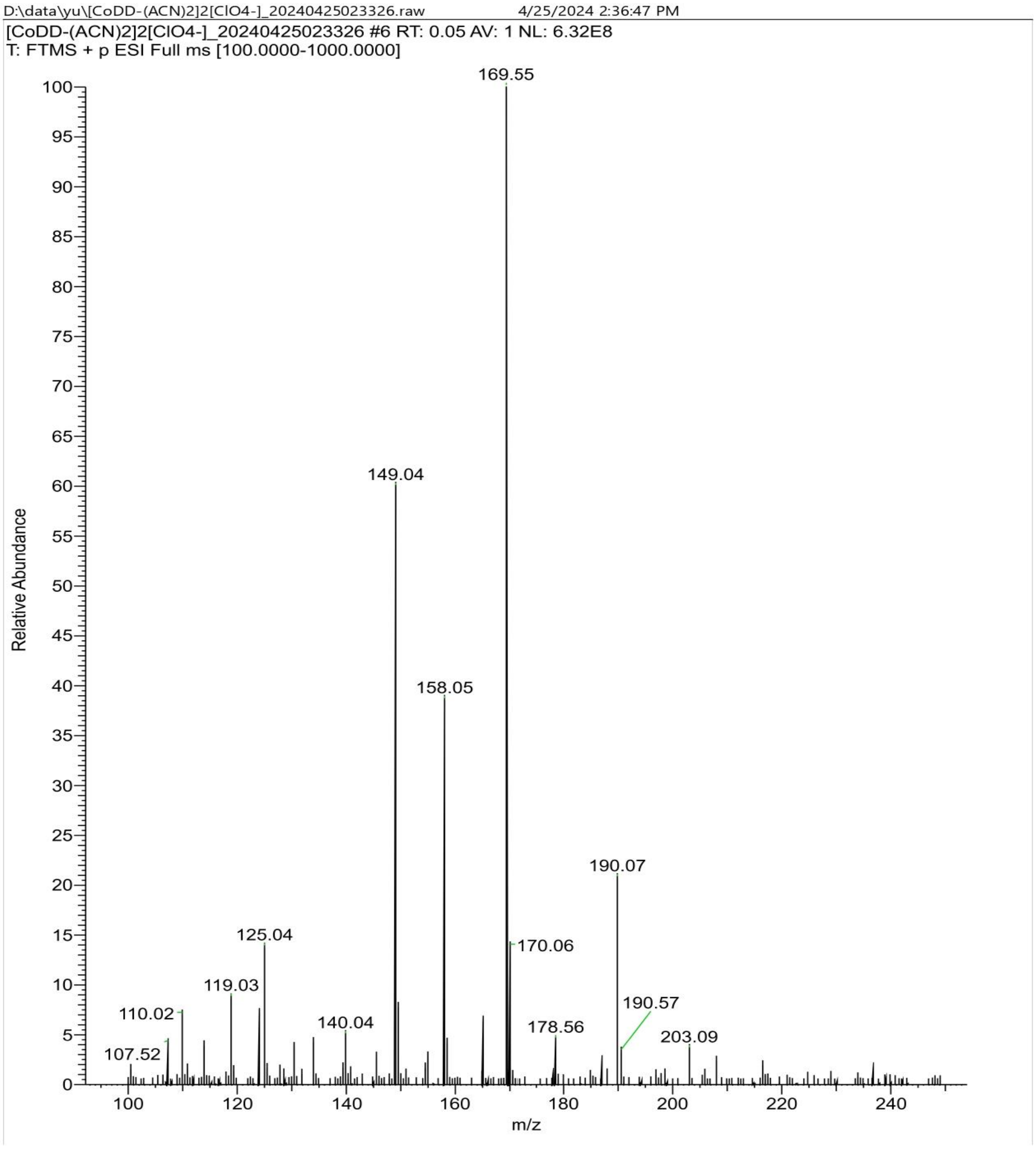

Figure S43. The low-resolution ESI-MS of complex **2**.

High resolution ESI-MS of complex 2

m/z calc. $[CoDD\text{-}(ACN)_2]^{2+}$:190.06800, found: 190.06792

m/z calc. $[CoDD\text{-}(ACN)]^{2+}$:169.55473, found: 169.55469

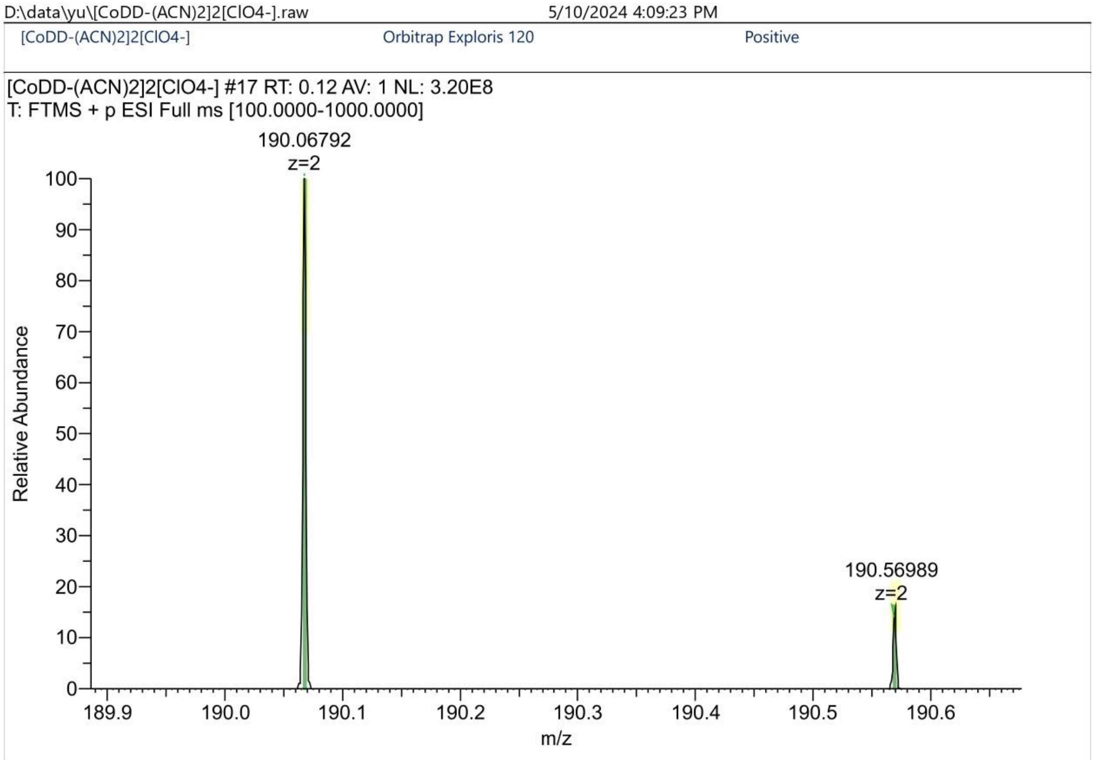

Peak Mass	Display Formula	S Fit	RDB	Delta [ppm]	Theo. mass	Pattern Cov. [%]	MSMS Matched...
190.06792	$C_{15}H_{25}O_2N_6{}^{59}Co$	43.24147833497 28	6.50	-0.44	190.06800	99.9	(Collection)

Figure S44. The high-resolution ESI-MS of complex **2**.

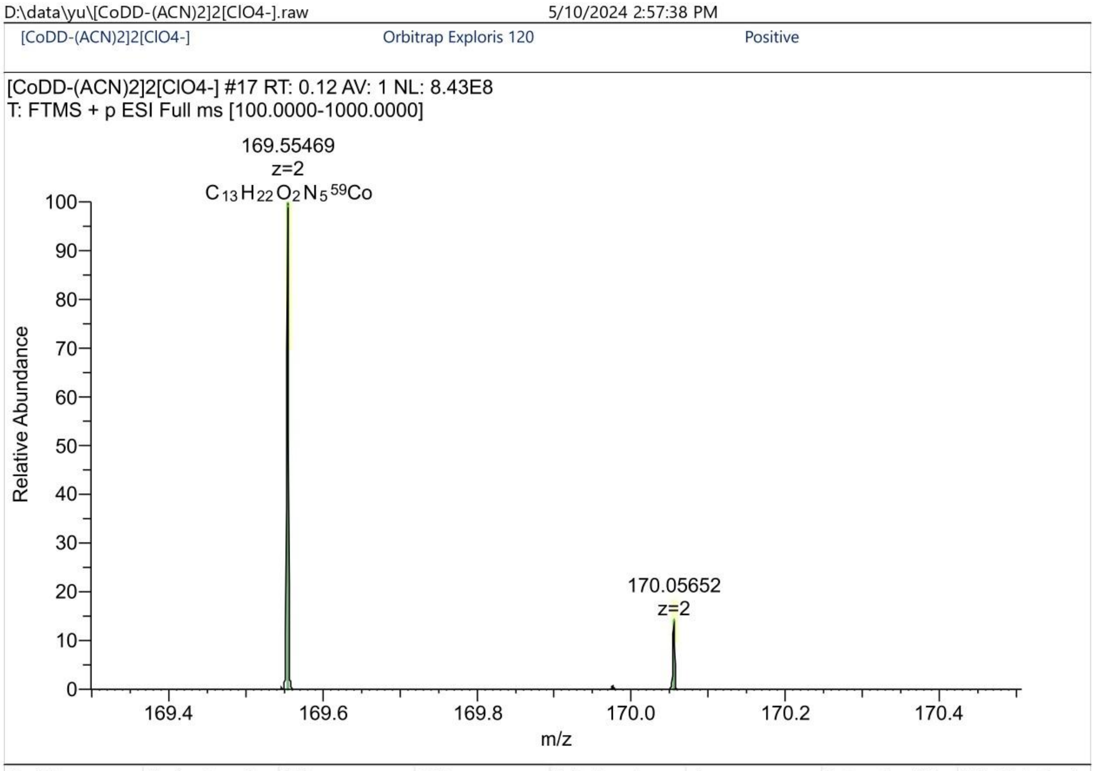

Peak Mass	Display Formula	S Fit	RDB	Delta [ppm]	Theo. mass	Pattern Cov. [%]	MSMS Matched...
169.55469	$C_{13}H_{22}O_2N_5{}^{59}Co$	65.482628125509	5.50	-0.23	169.55473	100	(Collection)

Figure S45. The high-resolution ESI-MS of complex **2**.

X-ray crystallography

Single crystals of CoDD-(ACN)$_2$-(ClO$_4$)$_2$ ($C_{16}H_{26.5}Cl_2CoN_{6.5}O_{10}$) were dissolved in acetonitrile, then layering by Et$_2$O at room temperature. A suitable crystal was selected and on a mylar loop in oil on a Bruker APEX-II CCD diffractometer. The crystal was kept at 100.00 K during data collection. Using Olex2,[11] the structure was solved with the SHELXT[12] structure solution program using Intrinsic Phasing and refined with the XL[13] refinement package using Least Squares minimization.

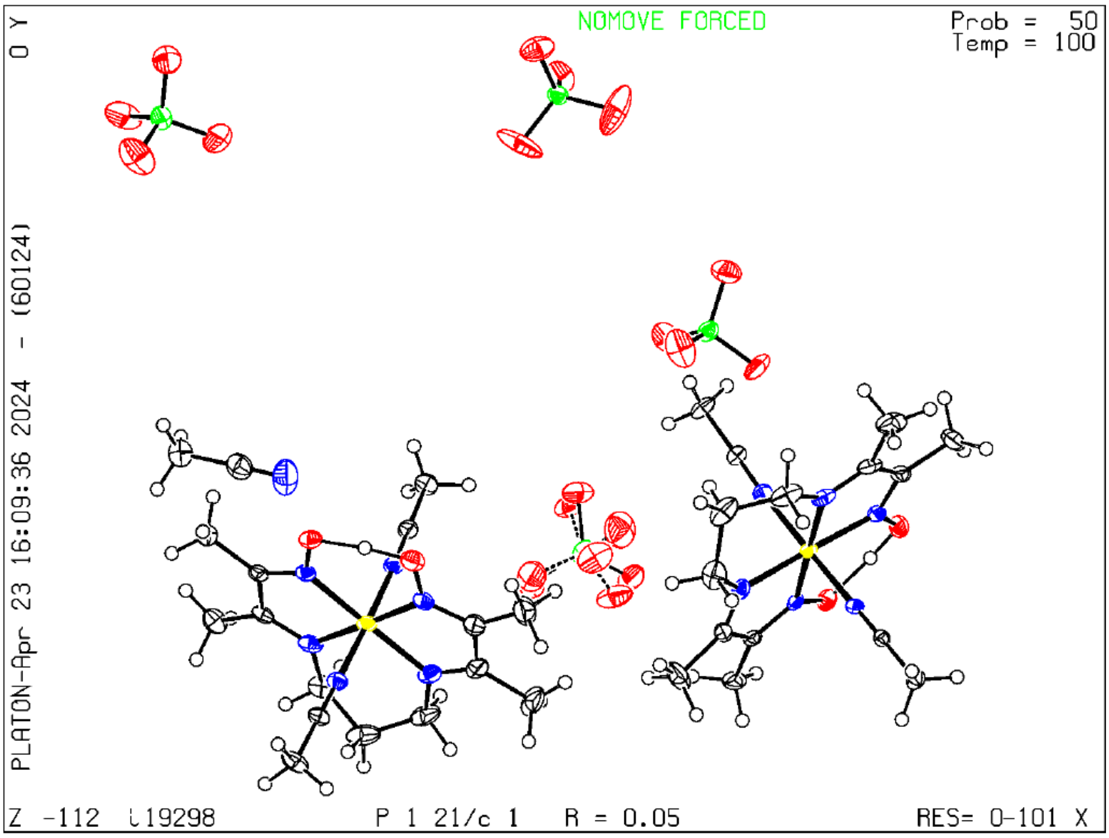

Figure S46. The X-ray crystallography of complex **2**.

Empirical formula	$C_{16}H_{26.5}Cl_2CoN_{6.5}O_{10}$
Formula weight	599.77
Temperature/K	100.00
Crystal system	monoclinic
Space group	$P2_1/c$
a/Å	17.2030(11)
b/Å	7.3738(4)
c/Å	38.4735(19)
α/°	90
β/°	96.337(3)
γ/°	90
Volume/Å^3	4850.6(5)
Z	8
ρ_{calc}g/cm^3	1.643
μ/mm^{-1}	0.993
F(000)	2472.0
Crystal size/mm^3	0.349 × 0.239 × 0.218
Radiation	MoKα (λ = 0.71073)
2Θ range for data collection/°	3.366 to 52.746
Index ranges	$-20 \le h \le 21$, $-9 \le k \le 9$, $-48 \le l \le 48$
Reflections collected	170074
Independent reflections	9919 [R_{int} = 0.1190, R_{sigma} = 0.0481]
Data/restraints/parameters	9919/194/698
Goodness-of-fit on F^2	1.055
Final R indexes [I>=2σ (I)]	R_1 = 0.0476, wR_2 = 0.1147
Final R indexes [all data]	R_1 = 0.0535, wR_2 = 0.1197
Largest diff. peak/hole / e Å^{-3}	0.88/-0.99

Table S4. Summary of X-ray crystallography data for complex

^{1}H-NMR of *p*-cyanoanilinium tetrafluoroborate

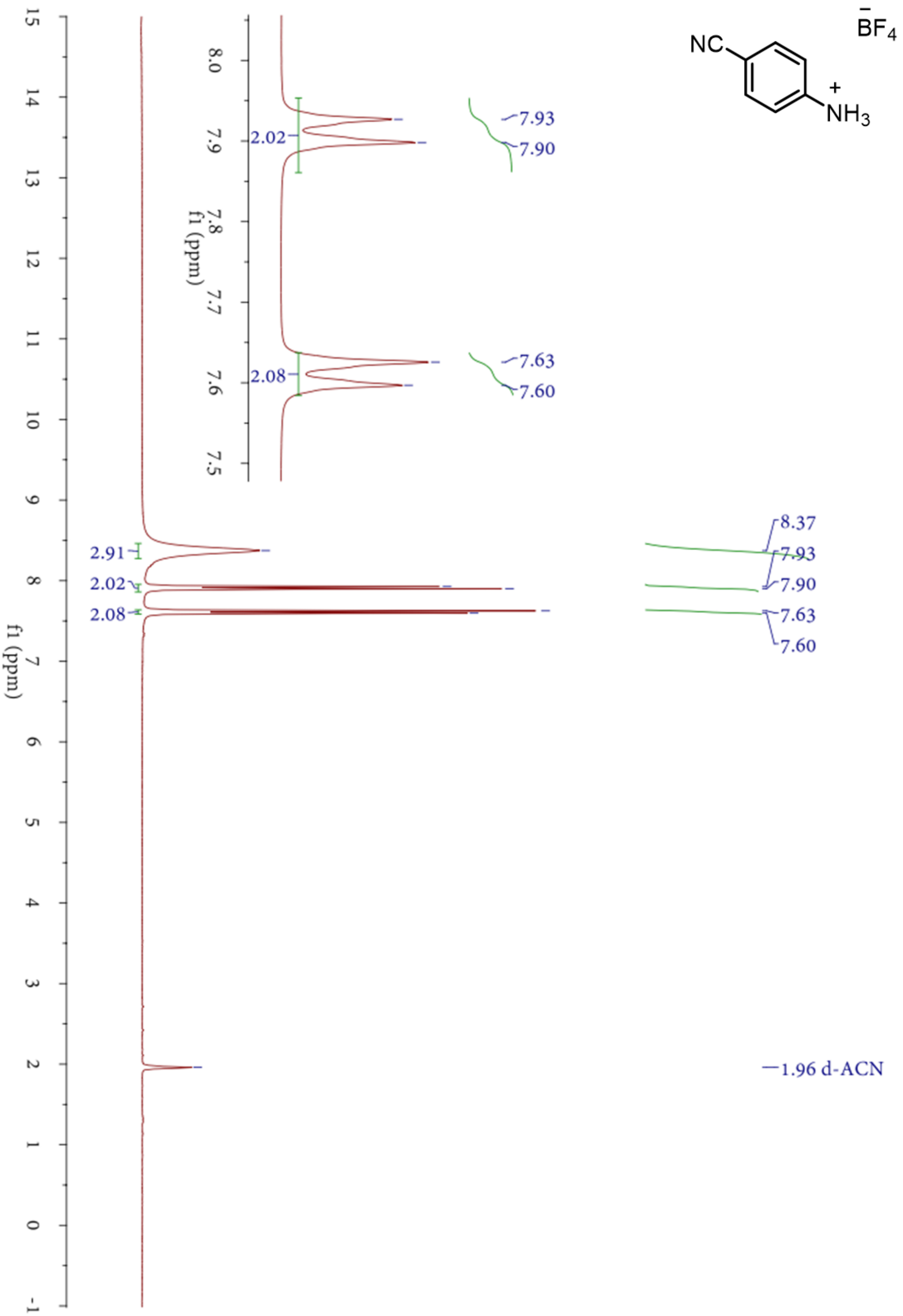

Figure S47. The ^{1}H-NMR of *p*-cyanoanilinium tetrafluoroborate.

^{1}H-NMR of *p*-chloroanilinium tetrafluoroborate.

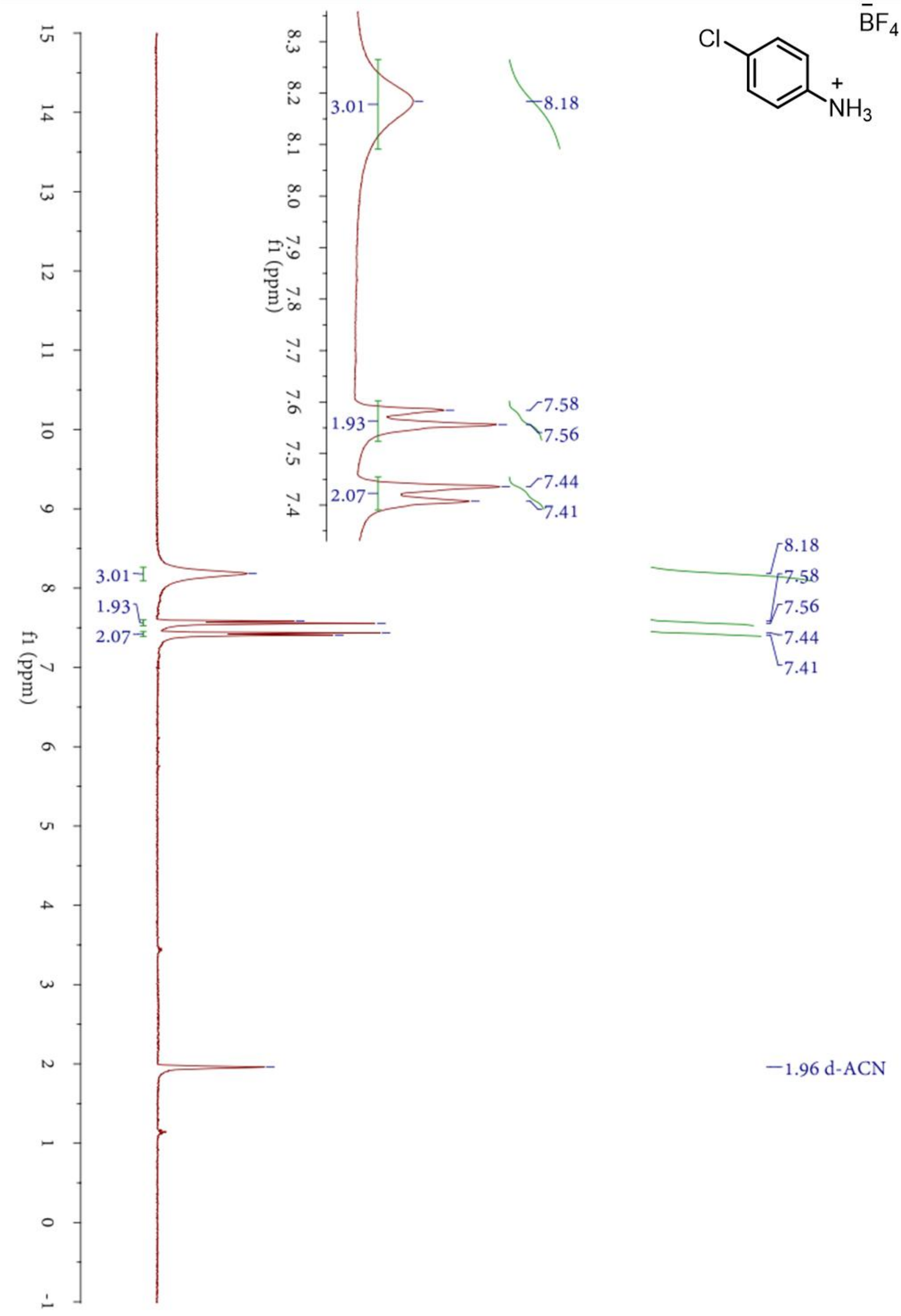

Figure S48. The ^{1}H-NMR of *p*-chloroanilinium tetrafluoroborate.

^{1}H-NMR of anilinium tetrafluoroborate

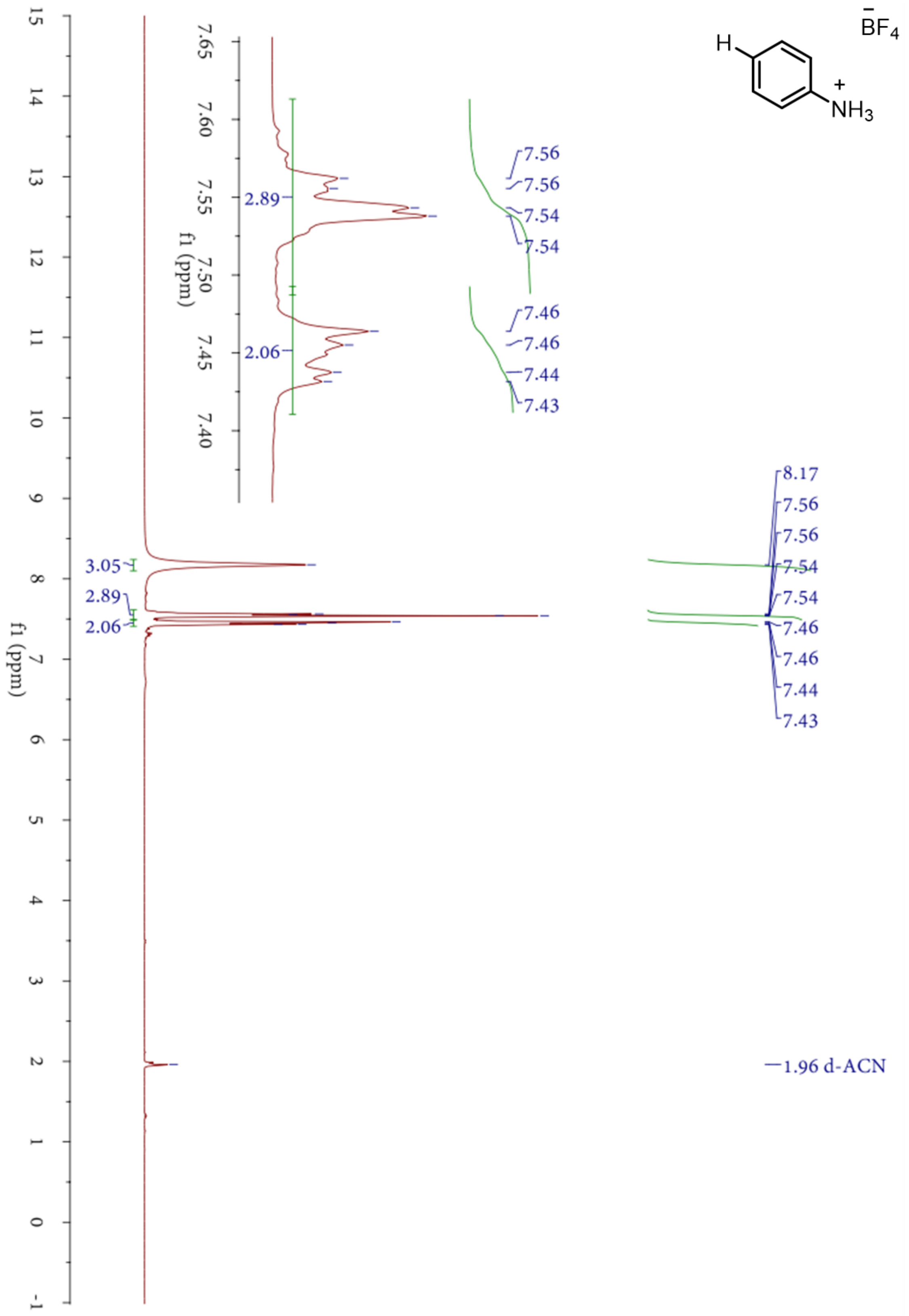

Figure S49. The ^{1}H-NMR of anilinium tetrafluoroborate.

^{1}H-NMR of *p-tert*-butylanilinium tetrafluoroborate

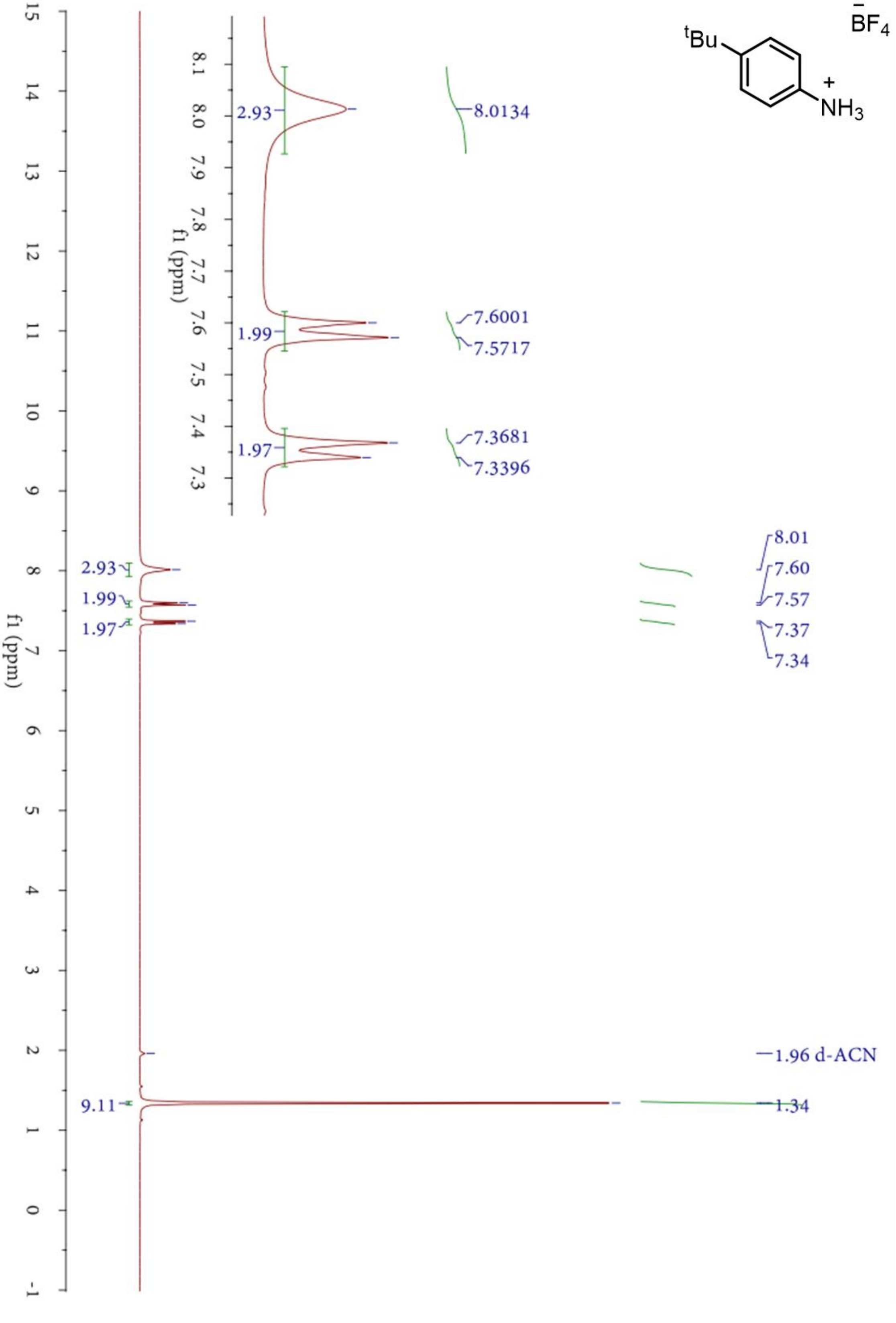

Figure S50. The ^{1}H-NMR of *p-tert*-butylanilinium tetrafluoroborate.

^{1}H-NMR of *p*-methoxyanilinium tetrafluoroborate

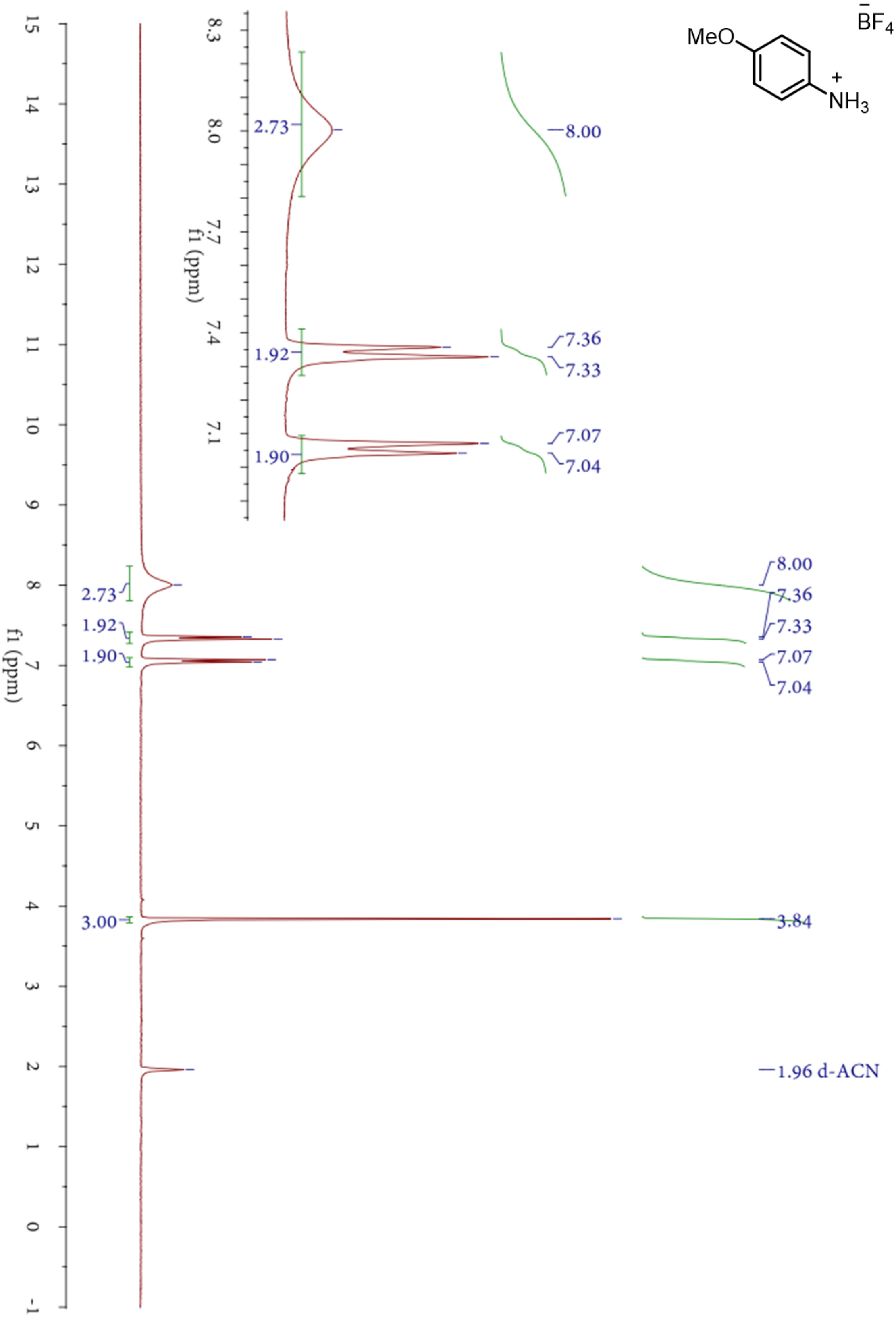

Figure S51. The ^{1}H-NMR of *p*-methoxyanilinium tetrafluoroborate.

References

1. Pegis, M. L.; Roberts, J. A. S.; Wasylenko, D. J.; Mader, E. A.; Appel, A. M.; Mayer, J. M. Standard Reduction Potentials for Oxygen and Carbon Dioxide Couples in Acetonitrile and *N,N*-Dimethylformamide. *Inorg. Chem.* **2015**, *54*, 11883-11888

2. Roberts, J. A. S.; Bullock, R. M. Direct Determination of Equilibrium Potentials for Hydrogen Oxidation/Production by Open Circuit Potential Measurements in Acetonitrile. *Inorg. Chem.* **2013**, *52*, 3823-3835

3. Rountree, E. S.; McCarthy, B. D.; Eisenhart, T. T.; Dempsey, J. L. Evaluation of Homogeneous Electrocatalysts by Cyclic Voltammetry. *Inorg. Chem.* **2014**, *53*, 9983-10002

4. Saveant, J. M.; Vianello, E. Potential-sweep chronoamperometry: Kinetic currents for first-order chemical reaction parallel to electron-transfer process (catalytic currents). *Electrochimica Acta* **1965**, *10*, 905-920

5. Gospodinova, N.; Terlemezyan, L. Conducting polymers prepared by oxidative polymerization: polyaniline. *Prog. Polym. Sci.* **1998**, *23*, 1443-1484

6. Lee, K. J.; Gruninger, C. T.; Lodaya, K. M.; Qadeer, S.; Griffith, B. E.; Dempsey, J. L. Analysis of multi-electron, multi-step homogeneous catalysis by rotating disc electrode voltammetry: theory, application, and obstacles. *Analyst* **2020**, *145*, 1258-1278

7. Wang, V. C. C. Beyond the Active Site: Mechanistic Investigations of the Role of the Secondary Coordination Sphere and Beyond in Multi-electron Electrocatalytic Reactions. *ACS Catal.* **2021**, *11*, 8292-8303

8. Wang, V. C. C.; Johnson, B. A. Interpreting the Electrocatalytic Voltammetry of Homogeneous Catalysts by the Foot of the Wave Analysis and Its Wider Implications.

ACS Catal. **2019**, *9*, 7109-7123

9. Savéant, J.-M.; Costentin, C., Coupling of Electrode Electron Transfers with Homogeneous Chemical Reactions. In *Elements of Molecular and Biomolecular Electrochemistry*, 2nd ed.; John Wiley & Sons: 2019; pp 81-181.

10. Pegis, M. L.; Wise, C. F.; Martin, D. J.; Mayer, J. M. Oxygen Reduction by Homogeneous Molecular Catalysts and Electrocatalysts. *Chem. Rev.* **2018**, *118*, 2340-2391

11. Dolomanov, O. V.; Bourhis, L. J.; Gildea, R. J.; Howard, J. A. K.; Puschmann, H. OLEX2: a complete structure solution, refinement and analysis program. *J. Appl. Cryst.* **2009**, *42*, 339-341

12. Sheldricka, G. M. SHELXT - Integrated space-group and crystal-structure determination. *Acta Crystallographica Section A* **2015**, *71*, 3-8

13. Sheldrick, G. M. A short history of SHELX. *Acta Cryst* **2008**, *64*, 112-122
